# Cost-effectiveness and affordability of community mobilisation through women’s groups and quality improvement in health facilities (MaiKhanda trial) in Malawi

**DOI:** 10.1186/s12962-014-0028-2

**Published:** 2015-01-10

**Authors:** Tim Colbourn, Anni-Maria Pulkki-Brännström, Bejoy Nambiar, Sungwook Kim, Austin Bondo, Lumbani Banda, Charles Makwenda, Neha Batura, Hassan Haghparast-Bidgoli, Rachael Hunter, Anthony Costello, Gianluca Baio, Jolene Skordis-Worrall

**Affiliations:** UCL Institute for Global Health, 30 Guilford Street, London, WC1N 1EH UK; Parent and Child Health Initiative (PACHI), Amina House, Western Wing – Second Floor, Capital City, P.O. Box 31686, Lilongwe 3, Malawi; Research Department of Primary Care & Population Health, UCL Priment Clinical Trials Unit, Royal Free Campus, London, NW3 2PF UK; Department of Statistical Science, University College London, 1-19 Torrington Place, London, WC1E 6BT UK; Epidemiology and Global Health, Umeå University, 901 87 Umeå, Sweden

**Keywords:** Cost-effectiveness, Affordability, Community mobilisation, Women’s groups, Quality improvement, MaiKhanda, Scale-up, Future scenarios, Malawi

## Abstract

**Background:**

Understanding the cost-effectiveness and affordability of interventions to reduce maternal and newborn deaths is critical to persuading policymakers and donors to implement at scale. The effectiveness of community mobilisation through women’s groups and health facility quality improvement, both aiming to reduce maternal and neonatal mortality, was assessed by a cluster randomised controlled trial conducted in rural Malawi in 2008–2010. In this paper, we calculate intervention cost-effectiveness and model the affordability of the interventions at scale.

**Methods:**

Bayesian methods are used to estimate the incremental cost-effectiveness of the community and facility interventions on their own (CI, FI), and together (FICI), compared to current practice in rural Malawi. Effects are estimated with Monte Carlo simulation using the combined full probability distributions of intervention effects on stillbirths, neonatal deaths and maternal deaths. Cost data was collected prospectively from a provider perspective using an ingredients approach and disaggregated at the intervention (not cluster or individual) level. Expected Incremental Benefit, Cost-effectiveness Acceptability Curves and Expected Value of Information (EVI) were calculated using a threshold of $780 per disability-adjusted life-year (DALY) averted, the per capita gross domestic product of Malawi in 2013 international $.

**Results:**

The incremental cost-effectiveness of CI, FI, and combined FICI was $79, $281, and $146 per DALY averted respectively, compared to current practice. FI is dominated by CI and FICI. Taking into account uncertainty, both CI and combined FICI are highly likely to be cost effective (probability 98% and 93%, EVI $210,423 and $598,177 respectively). Combined FICI is incrementally cost effective compared to either intervention individually (probability 60%, ICER $292, EIB $9,334,580 compared to CI). Future scenarios also found FICI to be the optimal decision. Scaling-up to the whole of Malawi, CI is of greatest value for money, potentially averting 13.0% of remaining annual DALYs from stillbirths, neonatal and maternal deaths for the equivalent of 6.8% of current annual expenditure on maternal and neonatal health in Malawi.

**Conclusions:**

Community mobilisation through women’s groups is a highly cost-effective and affordable strategy to reduce maternal and neonatal mortality in Malawi. Combining community mobilisation with health facility quality improvement is more effective, more costly, but also highly cost-effective and potentially affordable in this context.

**Electronic supplementary material:**

The online version of this article (doi:10.1186/s12962-014-0028-2) contains supplementary material, which is available to authorized users.

## Background

Maternal [1], perinatal and neonatal mortality in Malawi remain high [2]. Community and health facility-based interventions are needed to reduce these deaths [3] in Malawi and other high-burden countries in order to achieve millennium development goals four and five [4]. The MaiKhanda cluster randomised controlled trial (cRCT) in Malawi showed that combining community mobilisation through women’s groups (CI) and health facility-based quality improvement (FI) reduced neonatal mortality by 22% (95% CI: −40%, +1%; p = 0.057) [5]. The community intervention on its own reduced perinatal mortality by 16% (95% CI: −28%, −3%; p = 0.020); no significant effects of FI on its own were observed on neonatal, perinatal or maternal mortality or of CI and FICI combined on maternal mortality [5]. Given resource constraints in Malawi and other low-income settings where these interventions might be appropriate, policymakers need to know their cost-effectiveness and affordability relative to available alternative interventions. In this article we determine the cost-effectiveness and affordability of the community and facility-based interventions, both on their own (CI, FI), and in combination (FICI), as implemented by MaiKhanda in three central region districts of Malawi between 2007 and 2010.

We used a similar approach to costing as in other, similar trials with women’s groups as one of the interventions [6-9]. In evaluating the benefits, we focus on the primary outcomes of the interventions [10], extending the analysis reported in the trial paper [5] by taking into account all of the cRCT-measured effects on stillbirths, neonatal deaths and maternal deaths, irrespective of statistical significance. We determine the probability that each intervention is cost-effective at a range of Willingness-to-pay (WTP) threshold values ($ per DALY averted), including the per capita gross domestic product (GDP) of Malawi, and calculate the Expected Value of Perfect Information [11] to determine whether reducing uncertainty in the cRCT effect parameters is worthwhile. We complement our cost-effectiveness analysis with analyses of affordability [12] in relation to the number of potential beneficiaries in Malawi and available government and donor budgets for the interventions.

## Methods

In any cost-effectiveness analysis it is important to consider the alternatives being compared, perspective, time-horizon and discounting, component parts of the measures of the costs and effects of the intervention, and how all of these could vary [10]. Each of these is detailed below, along with details of how uncertainty surrounding measures was assessed. This section finishes with details of the mathematical and statistical analyses undertaken and outcome measures reported. Our reporting conforms to the CHEERS statement [13].

### Study setting

Malawi is a low-income, predominantly rural country in the rift valley of south-eastern Africa. It had an estimated population of 16.4 million people in 2013 [14]. Gross Domestic Product (GDP) per capita was estimated at $779.8 in purchasing-power-parity adjusted international dollars for 2013 [14]. Female literacy was estimated at 67.6% in 2010 [2] and female life expectancy at birth at 54.8 in 2012 [14]. The MaiKhanda trial was located in three central region districts of Malawi: Lilongwe, Kasungu and Salima, and involved the randomisation of health facility catchment populations (clusters) to either, both or none of the interventions, in a two-by-two factorial design [5,15].

### Alternatives compared

Each intervention is described in detail elsewhere [5,15]. Briefly, the community mobilisation intervention (CI) involves participatory women’s groups mobilising communities around maternal and neonatal health, using volunteer facilitators supported by programme staff. The groups follow an ‘action cycle’, adapted from previous studies [16-19], to identify and prioritise maternal and neonatal health problems, decide upon locally appropriate solutions, advocate for, implement and evaluate the solutions. 729 groups were set up, facilitated by 81 volunteers (who received bicycles, monthly bicycle maintenance allowances and supervision from 9 salaried MaiKhanda staff). Each group met monthly for an average of 16 times and strategies adopted included bicycle ambulances, vegetable gardens, health education, village savings and loans, and bednets.

The health facility quality improvement intervention (FI) involves ‘breakthrough series collaboratives’ [20]. A quality improvement team was set up in each health centre in order to share quality improvement ideas within and across facilities and implement them within their facilities in action periods. The intervention included training staff in quality improvement techniques, implementing change packages [21] focused on obstetric and newborn care, conducting death reviews [22], leadership training and specific additional protocol-based *in situ* clinical trainings. No new staff were recruited and no additional financial resources were provided to the facilities as part of the intervention evaluated in the trial.

The interventions were implemented in addition to current Government of Malawi Ministry of Health (MoH) practice in maternal and neonatal health. The incremental cost-effectiveness of the CI, FI and FICI interventions, were therefore calculated relative to ‘current practice’, which was assumed to be equal in intervention and control areas.

### Perspective

Cost effectiveness is calculated from the provider perspective. In any future scale-up of the intervention throughout Malawi, this will likely be the MoH. Given that the MoH relies significantly on donor funding, we have taken account of potential future trajectories for donor funding in our assessment of the affordability of the interventions.

### Costs

Women’s groups are a community intervention for which resource use can only be meaningfully identified at the community level. Therefore economic costing guidelines, which have been developed for individual-level variation in resource use, need to be adapted [6]. In our case, both interventions were implemented for each intervention arm as a whole, not cluster-by-cluster, meaning that it was not possible to identify cluster-level variation in resource use.

The main source of economic cost data were the financial accounts of MaiKhanda, the Malawian non-governmental organisation (NGO) implementing the interventions. Costs were also incurred by overseas implementing partners: the US-based Institute for Healthcare Improvement (IHI) and the UK-based Women and Children First (WCF). Key informant interviews and project documents were used to assist in the conversion of accounting costs to economic costs. We were unable to cost health worker’s time, therefore our costing of the FI is not a full economic-costing. For reasons stated in the discussion we believe it still reflects the replicable costs of implementation. Research costs, which were collected via a separate accounting system and therefore easily separable, are excluded for the purposes of this analysis. Project accounts were used to identify staff, material and capital costs directly associated with setting up and implementing the CI and FI interventions. We also identified joint (programme) costs, which could not be directly allocated to either intervention or to the other trial activities (stakeholder engagement, monitoring and evaluation, process evaluation, and research). A proportion of joint costs each year was allocated to the CI and FI interventions using joint cost allocation rules, which were arrived at through discussion with key informants [6]. Capital costs were annualized over the expected lifespan assuming constant linear depreciation.

Costs were converted to 2013 values using the Malawi consumer price index and to international dollars using the implied 2013 purchasing-power parity conversion factor for GDP (1 INT$ = 105.8 Malawi Kwacha) [14]. Costs incurred in Pounds Sterling (GBP; £) and United States Dollars (USD, $) were also converted into 2013 international dollars.

Not all external staff costs were available from accounting systems. Key informants were interviewed to estimate the costs of external experts using either their annual salary when applicable, or using the daily rate (averaged over the study period) of each named expert involved with the trial, the number of stays, and duration of each visit. External costs estimated in this way amounted to 607 days and are reported separately due to the different degree of accuracy compared to staff costs for which programme accounts data was available.

As intervention costs could not be disaggregated by trial cluster or arm, the cost of the combined FICI arm had to be estimated from the costs of the FI and CI interventions, both implemented across two arms. We assumed conservatively no economies of scope, in the sense that the cost of FI in the FI only arm was equal to the cost of FI in the combined FICI arm; and similarly for CI. In practice, when FICI costs are doubled to pertain to the same size population as FI and CI (Table 1), this is equivalent to assuming that the cost of the combined intervention is the sum of the two. In the trial analysis this is likely to be an accurate reflection of reality, because the CI and FI teams worked across trial arms. Our implementation design did not allow us to investigate possible cost savings that may arise from complementarity when implementing both interventions outside a trial setting.

**Table 1:** **Costs and effects of community, facility and combined interventions**

**Parameter**	**Community intervention (CI; 2 arms)**	**Facilities intervention (FI; 2 arms)**	**Combined FICI** ^**d**^		**Source**
			**(1 arm)**	**(2 arms)**	
Total population (babies and mothers)	1,200,000 (108,000)	1,200,000 (108,000)	600,000 (54,000)	1,200,000 (108,000)	
Comparisons data used for:	CI vs. current practice; CI vs. FI vs. FICI	FI vs. current practice; CI vs. FI vs. FICI	FICI vs. current practice	CI vs. FI vs. FICI	
Start-up cost, beginning ($)	362,083	362,860	362,472	724,943	MaiKhanda accounts
Maintenance costs, annual, on-going ($ per year)	27,250	25,328	26,289	52,578	
Annual implementation costs ($ per year)	2,068,997	2,026,811	2,047,904	4,095,808	
External costs ($ per year)	120,067	272,017	196,042	392,084	Estimated^a^
Total cost (27-month trial period)	5,348,791	5,592,212	5,470,501	10,941,002	Start-up, recurring and external costs
Baby mortality (OR, 95% CI)	0.873 (0.774, 0.982)	0.954 (0.846, 1.070)	0.841 (0.707, 0.992)	0.841 (0.707, 0.992)	model of cRCT data (Additional file 1)
Mother mortality (OR, 95% CI)	0.957 (0.494, 1.657)	1.228 (0.652, 2.135)	1.287 (0.488, 2.839)	1.287 (0.488, 2.839)	
Babies saved^b^ (mean, 95% CI)	772 (109, 1384)	291 (−439, 974)	475 (23, 880)	951 (46, 1760)	
Mothers saved^b^ (mean, 95% CI)	18 (−270, 210)	−97 (−480, 148)	−62 (−397, 112)	−124 (−793, 223)	
DALYs averted^c^ (mean, 95% CI)	67361 (8808, 121508)	19901 (−44769, 80586)	37590 (−4642, 74618)	75180 (−9284, 149236)	

$ constant 2013 international dollars; OR = Odds Ratio (mean); 95% CI = 95% Credibility Interval (2.5th centile, 97.5 centile); DALY = Disability Adjusted Life Year.

^a^From specific expatriate staff grades, percentage full-time and length of time working on the project, travel and hotel costs.

^b^Out of 108,000 for CI and FI, which is the estimated total population of babies and mothers in the relevant groups of two trial arms (CI, no CI, FI, no FI) in 27 months (2.25 years). This is the estimated total population of 1,200,000 (based on average size of health centre catchment area (cluster) [5]) multiplied by an estimated Crude Birth Rate (CBR) of 0.04 per person per year [2] for 2.25 years. For combined FICI it is out of 54,000, the estimated total population of babies and mothers in the relevant groups of one trial arm (FICI and control [5]).

^c^86.0 DALY averted per baby saved and 53.27 DALY averted per mother saved, see text for explanation.

^d^Note that the estimated costs and effects for FICI (first FICI column) were doubled (second FICI column) in order to compare the cost-effectiveness of FICI with the cost-effectiveness of CI and FI (Figure 4).

### Effects

The benefits of the interventions were assessed over the duration of the trial period: 1st October 2008 to 31st December 2010. The effects of the CI, FI and FICI interventions on maternal, neonatal and perinatal mortality have been previously reported as odds ratios (OR) [5]. The trial did not collect data on individual-level covariates except mortality. In this paper we use cluster-level totals of births, stillbirths, neonatal deaths and maternal deaths, along with cluster-level covariates (district and urban/rural cluster stratification and intervention designation of the clusters (CI, FI, FICI, control) to determine overall effects on stillbirths and neonatal deaths combined (baby mortality), and maternal deaths (mother mortality) [5]. These were modelled in separate logistic regression equations and combined to give an overall measure of effect.

Due to the two-by-two factorial nature of the trial [5], we first modelled the effects of two-arm CI vs. two-arm ‘no CI’ and two-arm FI vs. two-arm ‘no FI’, and then modelled the effects of one-arm FICI vs. one-arm control. The results of each model were saved in order to compare CI, FI and FICI in one cost-effectiveness analysis.

We converted the OR of CI, FI and FICI effects on baby and mother mortality obtained from the models to ‘deaths averted’ using the control area mortality rate and the number of live births, assumed to be 54,000 in each of the four arms. The number of births is an approximation because the population under surveillance was about 10-15% of the total population living in the intervention area.

The number of deaths averted was then converted to Disability Adjusted Life Years (DALYs) averted. The number of stillbirths and neonatal deaths averted was multiplied by 86.0, the standard life expectancy at birth used in the Global Burden of Disease 2010 study [23]. The number of maternal deaths was multiplied by 53.27, the remaining standard life expectancy of females aged 30 [24], the median age of maternal death in Malawi in 2010 [2]^Table 16.3, page 222^. As a sensitivity analysis, and as recommended by Polinder *et al.* [25], we also calculated ‘localised-DALYs’ using remaining healthy life expectancies specific to Malawi: 45.0 years, the healthy life expectancy at birth in Malawi in 2010 [26] for stillbirths and neonatal deaths averted, and 28.1 years, the healthy life expectancy at the median age of maternal death (30 years) in Malawi in 2010 [2]^Table 16.3, page 222^ [26,27] for maternal deaths averted. Consistent with the Global Burden of Disease 2010 study, no age weights or discounting were used in the calculation of DALYs [23]. We do apply discount rates to effects and costs in a modelling exercise. Note that, as measured in the trial [5], we assume all stillbirths are viable, and therefore apply the same DALYs averted as for a neonatal death [28].

### Analyses and outcome measures

We developed a Bayesian model to estimate the combined effects of each intervention as described above. We used Bayesian methods to take account of all of the available information, particularly the information on mortality averted, and to allow subsequent efficient and direct calculation of cost-effectiveness estimates and related parameters for decision-makers [11]. Given a lack of prior information on the effectiveness of the interventions or the covariates in the model we set priors for all model parameters to be zero (the log of an odds ratio of 1, which denotes the null hypothesis of no effect), with large uncertainty (variance of 10000 on the log scale). Equivalent parameters for the baby and mother mortality equations were modelled as multivariate normal distributions to improve the efficiency of estimation [11].

We modelled the effects with JAGS [29] in R [30]. For each model, 100,000 simulations were saved from two chains of 510,000 simulations, with a burn-in of 10,000 that were discarded and thinning of only every 10th simulation being saved to reduce autocorrelation [11]. The R package BCEA (Bayesian Cost Effectiveness Analysis) [31] was used with the estimate of total intervention costs to produce the following four key aspects of the results [11]. Additional file 1 contains the statistical models, R code including all details related to priors and initial values, and cluster-level data, used and should enable replication of our results.

1. Incremental cost-effectiveness ratios (ICERs) were determined for CI, FI and FICI interventions, using the following formula:

1 $$ \mathrm{ICER}\left(\$\ \mathrm{per}\ \mathrm{DALY}\right)=\frac{C_1-{C}_0}{E_1-{E}_0}=\frac{C_1}{E_1} $$

where *C*_1_ is the cost of the intervention in 2013 international dollars ($), *E*_1_ the effect of the intervention in DALY averted, and *C*_0_ and *E*_0_ respectively the costs and effects of the base case ‘current practice’, both zero because the ‘current practice’ costs and effects in the intervention and control areas are assumed to be equal and to therefore cancel each other out. For each comparison, the 100,000 simulations of *E*_1_ against the fixed *C*_1_ were plotted on the cost-effectiveness plane and the ICER calculated. E_1_ in equation (1) represents the mean effects of the intervention from all 100,000 simulations. In a final three-way comparison of CI, FI and FICI, ICER of FICI (*C*_1_, *E*_1_) and FI (*C*_1_, *E*_1_) were each calculated relative to CI (*C*_0_, *E*_0_).

2. Expected Incremental Benefit (EIB) is the monetary value of the net benefit of the intervention and was determined by multiplying the number of DALYs averted by the cost-effectiveness threshold (*k*) – the maximum cost per DALY averted that the provider might be willing to pay – and subtracting the difference in costs:

2 $$ \mathrm{E}\mathrm{I}\mathrm{B}\left(\$\right)=k\left({E}_1-{E}_0\right)-\left({C}_1-{C}_0\right)=k{E}_1-{C}_1 $$

3. Cost-effectiveness Acceptability Curves (CEAC) were calculated to show the probability that each intervention is cost-effective in comparison to current practice or each other, given the data (following our use of the Bayesian framework) and specified alternative *k*. Following others [11,12,32]^chapters 4 and 5^, probabilities of cost-effectiveness were calculated by determining the proportion of the simulations in which the reference intervention was more cost-effective than the comparator at a given $-per-DALY threshold, repeating at a range of threshold $-per-DALY values relevant to current and potential government health spending in Malawi, and plotting the results.
4. Expected Value of Information (EVI) was calculated to quantify the monetary ($) value of reducing uncertainty in the model parameters through additional research. It is calculated by comparing the EIB of the current decision with the probable EIB given additional information on the model parameters. EVI can be compared with the EIB (both at specified values of *k*) to determine if spending additional money on research to reduce parameter uncertainty might be worthwhile.

### Trial-based cost-effectiveness

Firstly, we report ICER, EIB, CEAC and EVI using the trial data. We assume the costs to be point estimates for the whole intervention prospectively collected during start-up and implementation (56 months) and the effects as distributions, estimated from the cRCT during the 27-month intervention period, as detailed above, and no discounting of costs or effects.

### Scale-up modelling exercise: Time-horizon and discounting

Secondly, we assess affordability and undertake a modelling exercise of cost-effectiveness and affordability based on future scenarios with differing available budgets, discount rates, and time-horizons. Costs were collected over a period of 4.6 years from May 2006 to December 2010. Activities in the two years before the start of the trial period in October 2008, included hiring and training research and implementation staff, developing and piloting the interventions, and introducing the interventions into village communities through the traditional leadership hierarchy. All costs incurred in 2006 were classified as start-up costs. When scaling up the intervention many of the activities conducted during this period, such as intervention design, would not be replicated. As such, only those start-up activities relevant to the intervention implementation, such as the purchase of vehicles and equipment and the hiring and training of staff to run the intervention, are included in the scale-up model. In 2007 50% of costs were classified as start-up because intervention implementation and the trial baseline period began in July 2007. Between July 2007 and December 2010 recruitment and training costs were classified as maintenance costs, with all other intervention-associated costs classified as implementation costs. The economic costs for all start up, maintenance and implementation activities required to replicate the intervention at scale, have been included in this model.

As recommended by the WHO, we go on to model cost-effectiveness over a 10-year time horizon [33]. Start-up costs from 2006 and 2007 are included in year one costs of the model. Maintenance costs for activities such as the regular recruitment and (re)training that would be required throughout an intervention, were annualised and included for each of years one to ten. Implementation costs for the 2007–2010 implementation period were annualised by dividing their total by the length of the trial period – the period when the effects were measured (2.25 years), and included for each of years one to ten. The trial period length was used to align costs with effects. Effects were also annualised by assuming the same effect per year as estimated over the 2.25 years of the trial period. Annualisation of costs and effects enables us to model cost-effectiveness over time horizons different to the observed trial period. We then applied the 3% annual discount rate recommended by the WHO [33], to the total costs for years two to ten. In sensitivity analysis, the time horizon was varied from 5 to 20 years, to reflect short-term and longer-term decision-making time frames, and to explicitly model the effect of intervention duration on the spread of setup and capital expenditure (among other fixed costs). The discount rate on costs was varied between 0% and 10% to reflect a plausible range of scenarios. Future effects (health benefits) were discounted at 2% and varied from 0% to 3% in different sensitivity analyses. It is likely that the discount rate for health benefits would be lower than the discount rate for costs [34,35], especially if the ($ per DALY) cost-effectiveness threshold increases in future, in-line with expected per-capita GDP growth [36]. Consistent with best practise and the Gates Reference Case [37], we also include the 3% discount rate for effects to show results of scenarios with equal discount rates.

### Affordability

Given the fixed costs of the interventions (no uncertainty in the costs due to no individual- or cluster-level costs being available) the affordability of the interventions in a hypothetical scale-up to the whole of Malawi was estimated deterministically. This was done using the following estimates of the available budget for maternal and neonatal health (MNH) in Malawi. In 2010, annual per capita expenditure on health in Malawi was $228.7 in constant 2013 international dollars, and the total population was 15,013,694 [14]. The percentage of total health expenditure spent on maternal and neonatal health was estimated to be 11.95% in 2010 [38]. Multiplying these figures yields an estimate of $410,354,347 for the total expenditure on MNH in Malawi in 2010. This estimate frames the available budget for affordability calculations.

To estimate the cost of scaling-up each intervention to the whole of Malawi we multiplied the annualised cost by a ratio of the total population of mothers and babies (estimated from the total population and the crude birth rate) to the population of mothers and babies covered in the trial, for that intervention. We then present affordability as a percentage of total annual MNH expenditure. 2010 was used for these analyses due to data availability, especially for estimation of DALYs averted from current practice interventions. We used the latter to compare percentage expenditure with expected percentages of remaining DALYs averted by the interventions in scale-up scenarios across Malawi (Additional file 2, and discussion).

## Results

Table 1 summarises the costs and effects for each of the three interventions. The community intervention cost a total of $5,348,791 of which $362,083 were start-up costs, $2,068,997 annual implementation costs, $27,250 annual maintenance costs and $120,067 were annual external (overseas) staff costs. The facility intervention had similar start-up costs, higher external costs, but slightly lower annual implementation costs, resulting in slightly higher total costs for the trial period, of $5,592,212.

The mean newborn deaths (DALYs) averted by the community, facility and combined interventions were respectively estimated as 772 (66,409), 291 (25,048) and 951 (81,772). The mean effect of CI on maternal mortality was 18 deaths averted, while the mean effect of FI and combined FICI was negative (97 and 124 extra maternal deaths respectively), though the 95% credible intervals were very wide. The combined effect was 67,361, 19,901 and 75,180 DALYs averted, with only the 2.5th centile of the posterior distribution of the effect of the community intervention being above zero, denoting a significant effect with 95% credibility. Additional file 1 contains full results for all model parameters and model diagnostic statistics.

**Figure 1:**
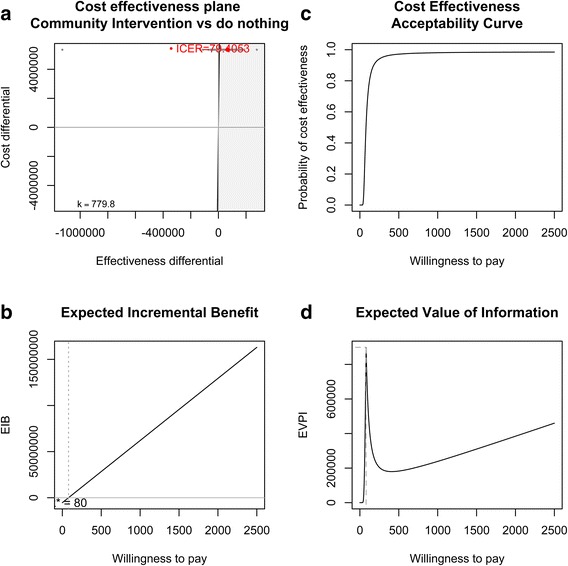
**Community Intervention (CI) vs. current practice: a) cost effectiveness plane and Incremental Cost-Effectiveness Ratio (ICER), b) Expected Incremental Benefit (EIB), c) Cost-effectiveness Acceptability Curve (CEAC), d) Expected Value of Information.**

Figure 1 plots the ICER, EIB, CEAC and EVI results of the community intervention compared to current practice. Figure 1a shows the results of each of the 100,000 saved Monte Carlo simulations on the cost-effectiveness plane (note the constant costs but uncertain effects) and shows the ICER of the community intervention in comparison to current practice to be $79 per DALY averted. Figure 1b shows a linear increase in EIB as WTP increases above the break-even point of the ICER. At a WTP of $780 per DALY (approximately the WHO-recommended threshold for ‘highly cost-effective’ interventions – the per capita GDP [33]) the EIB is $47,192,509. Figure 1c shows the CEAC. At WTP thresholds above the ICER of $79 per DALY averted the probability of cost-effectiveness increases past 50%, reaching 98% at a WTP of $780 per DALY. Figure 1d shows the EVI reaches a maximum of around $900,000 when the WTP threshold equals the ICER of $79 per DALY, the point where we are most uncertain of the cost-effectiveness. At the WTP threshold of $780 per DALY the EVI is even lower at $210,423.

**Figure 2:**
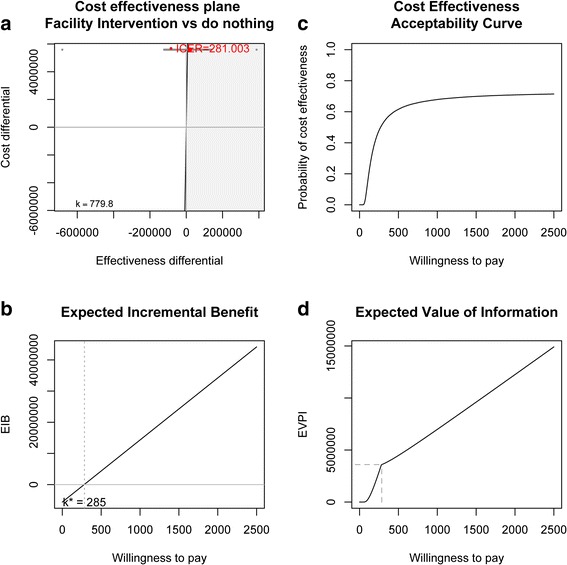
**Facility Intervention (FI) vs. current practice: a) cost effectiveness plane and Incremental Cost-Effectiveness Ratio (ICER), b) Expected Incremental Benefit (EIB), c) Cost-effectiveness Acceptability Curve (CEAC), d) Expected Value of Information.**

Figure 2 shows the results of the facility intervention compared to current practice. The ICER is $281 per DALY averted and the probability of cost-effectiveness only increases to 66% at the per capita GDP WTP threshold of $780 per DALY averted and does not reach 70% even at thresholds of $2500 per DALY. At $780 per DALY averted the EIB is $9,930,509 and the EVI is $5,851,010.

**Figure 3:**
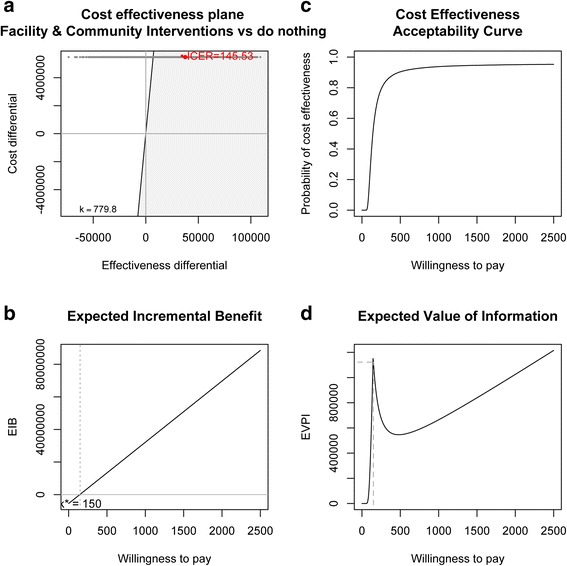
**Facility and Community Interventions combined (FICI) vs. current practice: a) cost effectiveness plane and Incremental Cost-Effectiveness Ratio (ICER), b) Expected Incremental Benefit (EIB), c) Cost-effectiveness Acceptability Curve (CEAC), d) Expected Value of Information.**

Figure 3 compares the facility and community interventions combined, with current practice. As expected, the ICER is between that of the individual community and facility interventions, at $146 per DALY averted. At a threshold of $780 per DALY averted the probability of cost-effectiveness is 93%, the EIB is $23,849,857 (note the population covered by the combined intervention is half of that covered by each of the individual interventions) and the EVI is $598,177.

**Figure 4:**
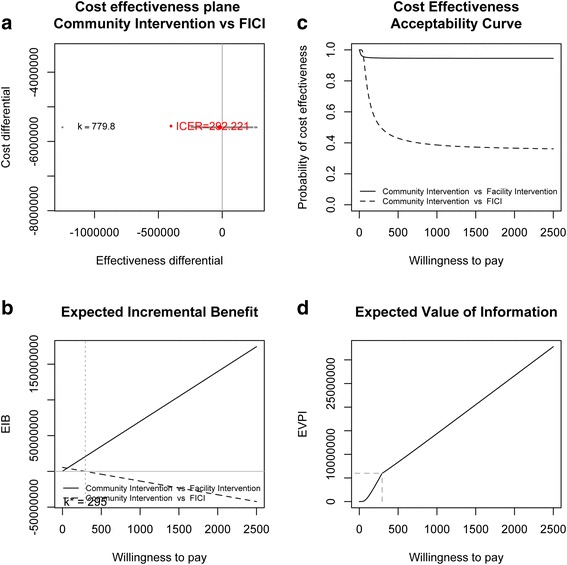
**Community Intervention (CI) vs. Facility Intervention (FI) vs. Facility and Community Interventions combined (FICI): a) cost effectiveness plane and Incremental Cost-Effectiveness Ratio (ICER), b) Expected Incremental Benefit (EIB), c) Cost-effectiveness Acceptability Curve (CEAC), d) Expected Value of Information.**

Figure 4 shows the results of comparing the community intervention with the facility intervention, and the facility and community interventions combined. Comparing the community intervention to the facility intervention, the ICER is -$3 (Additional file 1). This means that the community intervention strictly dominates the facility intervention, as it is both more effective and less costly than the facility intervention. At a WTP of $780 per DALY, it is 95% probable that the community intervention is cost-effective compared with the facility intervention.

Figure 4a shows the ICER for the community intervention compared with the combined intervention, the next most cost-effective of the three interventions. The incremental cost of FICI compared to CI is $5,592,212 (i.e. the cost of FI), the mean expected incremental effect 11,967 DALYs averted, and the ICER is $292 per DALY averted, suggesting that at WTP thresholds above this, the combined intervention is the optimal decision. Indeed at the threshold of $780 per DALY it is 60% probable the combined intervention is incrementally cost-effective compared to the community intervention. At $780 per DALY the EIB for the combined intervention is $9,334,580 more than that for the community intervention, and the EVI is large at $11,708,726.

Using local life expectancy to calculate DALYs averted supports the same decisions at key threshold values. Though the ICERs were larger and probabilities of cost-effectiveness and EIB lower, all mean ICERs were below the threshold of $780 per DALY and in the same order, with CI having the lowest ICER when compared to current practice, and FICI being the optimal intervention when comparing all three at the $780 threshold (Additional file 3).

Scaling-up to cover all 1,201,906 estimated mothers and babies each year in Malawi would cost an estimated $27,729,225 and $29,078,474 per year for the community and facility interventions respectively, and $56,807,699 for both combined (Table 2). These totals represent 6.8%, 7.1% and 13.8% of the estimated $410,354,347 total annual expenditure on MNH in Malawi in 2010 (Table 2).

**Table 2:** **Scale-up and Affordability of the interventions in 2010**

	**Trial (per year)**	**Nationwide scale-up (per year)**	**ratio**
Population (mothers and babies^a^)	96,000^b^	1,201,096^c^	12.51
CI annual implementation cost^d^ ($)	2,216,315	27,729,225	12.51^e^
FI annual implementation cost^d^ ($)	2,324,156	29,078,474	12.51^e^
FICI annual implementation cost^d^ ($)	4,540,471^f^	56,807,699	12.51^e^
CI cost per person^g^ ($)	23.09	23.09	1
FI cost per person^g^ ($)	24.21	24.21	1
FICI cost per person^g^ ($)	47.30	47.30	1
Total MNH expenditure in Malawi ($)		410,354,347	
per person expenditure on MNH in Malawi ($)		341.65	
proportion of MNH budget spent on CI		6.8%	
proportion of MNH budget spent on FI		7.1%	
proportion of MNH budget spent on FICI		13.8%	

$ constant 2013 international dollars; CI = Community Intervention; FI = Facility Intervention; FICI = Facility and Community Interventions combined; MNH = Maternal and Neonatal Health.

^a^Only one baby per mother per year assumed on average.

^b^This is the estimated total population of 1,200,000 (based on average size of health centre catchment area (cluster) [5]) multiplied by an estimated Crude Birth Rate (CBR) of 0.04 per person per year [2], multiplied by two to reflect the mother and the baby separately.

^c^Total population of Malawi in 2010: 15,013,694 [14] multiplied by CBR of 0.04 multiplied by two.

^d^Recurring plus external costs (see Table 1).

^e^Ratio of trial to national population applied to trial costs to estimate nationwide costs per year.

^f^This is the recurring plus external costs of FICI from the trial multiplied by two to reflect what the cost of FICI would have been if in the same area (two arms) of the trial as CI or FI (so that the trial to scale-up population ratio is still valid).

^g^Mother and baby counted separately.

With discount rates of 3% for costs and 2% for effects and a 10-year time horizon of a proportional scale-up of the annual intervention costs and effects from the trial to the whole of Malawi, comparing CI, FI and FICI at a cost-effectiveness threshold of $780 per DALY, FICI is the optimal intervention, with an EIB of $603,598,757 and probability of cost-effectiveness of 83.9% compared to CI (Additional file 4). Sensitivity analyses varying the time horizon between 5, 10 and 20 years, the annual cost discount rate between 0%, 3% and 10%, and the annual effects discount rate between 0%, 2% and 3% found, at the $780 per DALY threshold, FICI to have the highest probability of cost-effectiveness in all scenarios (Additional file 4). This varied from 73.8% probability of cost effectiveness (EIB: $286,587,031; 5 years, 0%, 3%) to 94.9% probability of cost effectiveness (EIB: $1,654,218,349; 20 years, 10%, 0%; Additional file 4). The ICER of FICI compared to CI (the threshold value at which FICI becomes the optimal decision) ranged from $103 to $322 across the 27 scale-up scenarios (Additional file 4). FI was dominated in all scenarios.

## Discussion

This analysis measured and compared the cost-effectiveness of the MaiKhanda interventions, relative to current practice and to one another. We used novel methods to consider the whole of the estimated distributions of the effects of the interventions on maternal mortality, neonatal mortality and stillbirths from the trial [5]. We estimate the community intervention to be most cost-effective, with an ICER of $79 per DALY averted compared to current practice alone. This is followed by the combined FICI intervention with an ICER of $146 per DALY averted compared to current practice, and the facility intervention with an ICER of $281. Comparing all three, the community intervention is the optimal decision at WTP thresholds below $292 per DALY averted. Above this, including at the per capita GDP per DALY averted ‘highly cost-effective’ threshold [33] of $780 the FICI intervention is cost-effective.

At $56,807,699 per year for the whole of Malawi, FICI is considerably more expensive than CI at $27,729,225. Either amount, at 13.8% and 6.8% of the current MNH budget (Table 2), respectively represents significant expenditure for Malawi. Given the continuing high burden of maternal and perinatal mortality and morbidity in Malawi and the observed cost-effectiveness of interventions such as CI and FICI an increase in the total MNH budget is clearly required. This could be achieved either via increased government funding following increased economic growth or more efficient tax collection in Malawi, or via expanded donor interest and funding. If additional funds are not forthcoming reallocation of MNH funding away from current activities likely to be less cost-effective than CI or FICI should also be considered.

Our EVI analyses suggest further research is worthwhile to resolve remaining uncertainties surrounding the CI or FICI decision. At the $780 per DALY WTP threshold, the EVI of $11,708,726 is not significantly different from the cost of the trial at $13,538,490 (note this includes the research costs of $2,597,487). It is clear that the CI (in addition to current practice) is cost effective compared with current practice only. Therefore, unless the WTP threshold is very low, there is no need to invest in additional research to resolve the remaining uncertainties surrounding this decision. Delays in implementing CI at this stage are more likely to result in benefits foregone, in particular neonatal deaths and stillbirths. The facilities intervention was dominated by the community intervention and the combined intervention, suggesting money would be better spent first on scaling up the community intervention and then the combined intervention.

Using natural history counterfactuals, as recommended by WHO for estimating the cost-effectiveness of current interventions [33], we estimate the cost-effectiveness of ‘current practice’ interventions to be roughly $51 per DALY averted (see Additional file 2 for assumptions and calculations). This is less than our estimates (looking at the same three mortality outcomes only) of the cost-effectiveness of the community intervention ($79 per DALY) and substantially less than the combined intervention ($146 per DALY) or the facility intervention ($281 per DALY).

However, the most cost-effective interventions are likely to have already been funded to bring down the mortality rates to their 2010 level, and more intensive and expensive interventions are usually required to reduce mortality rates further. Therefore the community intervention in particular can be said to be comparatively cost-effective and indeed fares well when compared to the interventions currently included in the Essential Health Package (EHP) in Malawi (Additional file 5) [39,40] and recommended to improve maternal and neonatal health in Africa [41]. Also, assuming, based on the estimated mortality rates in 2010 that there were 2,892,134 DALYs from maternal deaths, neonatal deaths and stillbirths remaining, it can be deduced that the CI, FI and FICI interventions could have prevented 13.0%, 3.8% and 14.5% of these remaining DALYs if scaled-up throughout the whole of Malawi (see Additional file 2 for sources and calculations). Compared to the additional 6.8%, 7.1% and 13.8% respective additional expenditures on MNH the CI, FI and FICI interventions could entail (Table 2) this represents a particularly good investment for the CI.

Whether fiscal space is available to invest in the MaiKhanda interventions depends on competing budget priorities and projections. It will also depend on donor priorities given that 60% of health expenditure in Malawi was donor-funded between 2002–2006 [42] and an estimated 89% in 2013–2014 [43]. Our future projections for scale-up to the whole of Malawi indicated that at the $780 per DALY cost-effectiveness threshold FICI is likely to be the optimal decision, and at thresholds lower than $103, CI is likely to be optimal (Additional file 4).

We hope that including analyses of affordability based on recent expenditure on MNH as well as cost-effectiveness with comparison to the EHP will make our study useful for policy makers in Malawi and help them to decide whether investment in and scale-up of the MaiKhanda interventions are worthwhile. To aid decision-makers we have determined cost-effectiveness in relation to a range of $-per-DALY thresholds, with a focus on the per capita GDP of Malawi, recommended by WHO to represent ‘highly cost-effective’ interventions [33]. Although there are alternative methods of threshold setting [44], we believe this threshold to be valid and perhaps more reflective of the current EHP than the more arbitrarily-set threshold of $150-per-DALY (Additional file 5) [44]. We also hope our study will be useful for researchers, particularly because we have been transparent with our data, methods and assumptions to enable replication and use of our results.

Our study has three important limitations. Firstly, we were not able to capture cluster-level variation in resource use because the interventions were not delivered cluster-wise but taking all randomised clusters as one intervention area. Therefore we were not able to take account of the correlation between costs and effects at the cluster level. We are aware of recent recommendations for cost-effectiveness analysis of cRCTs [45,46] that draw attention to the clustering of costs. However, these recommendations are of limited relevance to us because the recipient of the MaiKhanda interventions was the community or health facility, not the individual mother or child. Identifying individual-level resource use would not be conceptually meaningful (nor possible) in our situation in which many members of the study population never participated in a women’s group nor had contact with a health facility.

Secondly, our cost analysis adopted a limited intervention provider perspective. We did not include related health sector costs, such as the costs of increased care-seeking that may result from a combined ‘pull’ of the supply side intervention (FI) and ‘push’ of the demand side intervention (CI). However, the trial found no significant differences in utilisation of health services for childbirth across arms [5] ^supplementary figure^; data on antenatal or postnatal care was not collected. Providing cover for staff attending the Quality Improvement (QI) workshops was not considered. Given chronic staff shortages in Malawi [47] it is very unlikely that absent staff would be covered. Importantly, the effect on mortality of staff brought in to cover absent staff would also remain unknown. Neither did we include potential cost savings that may arise in particular in the FI intervention as a result of a reduction in staff attrition. It is possible that the time health workers spent on the quality improvement activities of FI whilst at work in their health facilities could be spent on other, more effective, activities. This is an area for further research and we are not aware of any existing economic evaluations of similar interventions with time use data that explored this question. Donations and volunteer inputs were also not included. However, these were minimal, such as limited drug and equipment donations to a few facilities. The community intervention relied on volunteer women’s group facilitators as an integral part of the intervention and defining characteristic of the MaiKhanda trial. Given costs were prospectively collected, the only major source of uncertainty in our cost estimates was the allocation of joint costs to each intervention. Based on the agreement of the donor and the partner organisations implementing each intervention, we chose an equal allocation of joint costs for FI and CI based on the equal – proportional to implementing staff number – allocation of office space and equal use of administrative staff and joint programme resources. Joint costs were 46% of CI and 53% of FI costs, therefore introducing, say, +/− 5 percentage points uncertainty in the allocation rule would result in the total cost estimates for each intervention varying by +/−2.3% and +/−2.7% respectively. Assuming symmetrical uncertainty and that each intervention would have an equal (proportional to need) joint cost allocation as per the implementing programme partner’s agreement, the ICERs for each intervention – which as a ratio of the expected value of two uncertain parameters have no uncertainty themselves - would remain unchanged.

Thirdly, our analysis is likely to present an underestimate of the full effects of the interventions. In particular, estimating the effects on maternal and neonatal morbidity was beyond the scope of this study. Moreover, the community intervention could empower women and other members of the community, and improve a variety of areas of quality of life including those related to physical and psychological health, social relationships and the environment [48]. The facility intervention could improve staff morale, motivation and empowerment and reduce staff attrition. Both interventions could also improve links between communities and health facilities. Measurement of all of these potential benefits were also beyond the scope of the current study due to its resource constraints, although small sample sub-studies on many of these measures were undertaken [15,48].

We also made a number of assumptions within our analyses. The international travel and salary costs of the external technical experts that contributed to the community and facility interventions have been included as recurring costs throughout the modelled time horizons. From the perspective of the MoH these costs may be determined less necessary if local experts could be used instead at lower cost. However, as per the trial, local experts may not be available, and even if available they might steer the interventions in a different direction, which could result in different effects as well as lower costs. We have also assumed that the costs and effects of each of the interventions increase proportionally with any scale-up of the interventions. Further research is needed on how the costs (and effects) of the interventions may change at scale. For the costs, economies-of-scale are possible via a number of mechanisms including decreases in unit prices when increased quantities of equipment and materials are purchased, and cost-sharing resulting from the MoH or other NGOs sharing activities in any scale-up. However, given that the interventions are mainly reliant on human resources (55% and 49% of the community and facility intervention cost, respectively), such economies-of-scale may not be significant. How the process of scaling-up affects staff deployment should nevertheless be investigated. Variations in prices by region of the country may also be important when estimating scale-up costs. Health effects may not be independent of scale, especially if the population coverage and density of the interventions is not kept at the same level as the cRCT in the scale-up [9]. Complex interventions such as the MaiKhanda community and facility interventions are also unlikely to be replicated in exactly the same way. Although processes and function should be standardised to some extent, exact content will depend on local context [15,49], meaning costs and effects of any scale-up are likely to be different to the trial-based estimates we have used.

As with any cost-effectiveness results our results should be considered along with other health system goals such as equity and acceptability to stakeholders [50]. Information on equity of distribution and acceptability of the interventions to different stakeholders remains unavailable, although an on-going study [51] is investigating the equity of the distribution of the effects of the women’s group intervention [52] in Malawi. Future research to refine our assumptions and address some of the limitations of our study would be worthwhile. A cost-effectiveness analysis of all women’s group trials and potential scale-up as reported in Prost *et al.* [9] is in progress.

## Conclusions

Community mobilisation through women’s groups is, according to these analyses, both cost-effective and affordable, compares well with current interventions in Malawi and could avert a large proportion of DALYs caused by stillbirths, neonatal and maternal deaths considering the proportion of the budget it would require. When combined with facility quality improvement it is highly cost-effective and affordable, and at a per capita GDP threshold the combined intervention warrants scale-up throughout Malawi. Additional research could reduce the remaining uncertainty surrounding this decision and assist decision-makers in similar settings that could also benefit from these interventions.

## Additional files

Additional file 1:
**Models, cluster-level mortality data, R source code and output.**
Additional file 2:
**Calculation of MNH DALYs averted in Malawi in 2010 with ‘current practice’ interventions, cost-effectiveness of ‘current practice’ interventions, and proportion of total remaining DALYs the MaiKhanda interventions could avert.** [1,2,14,23,24,38,53-56]. Additional file 3:
**Comparison of results with standard and local life expectancies.** The number of deaths averted (the effects of the interventions) were converted to Disability Adjusted Life Years (DALYs) averted. As the base-case, ‘standard’ life expectancy was used to calculate DALYs. The number of stillbirths and neonatal deaths averted was multiplied by 86.0, the standard life expectancy at birth used in the Global Burden of Disease 2010 study [23]. The number of maternal deaths was multiplied by 53.27, the remaining standard life expectancy of females aged 30 [24], the median age of maternal death in Malawi in 2010 [2]^Table 16.3, page 222^. As a sensitivity analysis, and as recommended by Polinder *et al* [25], we also calculated ‘DALYs’ using ‘local’ life expectancy: remaining healthy life expectancies specific to Malawi: 45.0 years, the healthy life expectancy at birth in Malawi in 2010 [26] for stillbirths and neonatal deaths averted, and 28.1 years, the healthy life expectancy at the median age of maternal death (30 years) in Malawi in 2010 [2]^Table 16.3, page 222^ [26,27] for maternal deaths averted. The results of both analyses are shown side-by-side in the table to aid comparison. Additional file 4:
**Future Scale-up scenarios.** Scale-up to the whole population of Malawi, using the trial population to total population ratio explained in Table 2 of the paper. Annual maintenance, implementation and external costs were used from Table 1 of the paper, and were discounted using the costs discount rate for the scenario and added to the scaled-up beginning start-up costs. Effects were annualised by dividing the estimates from the 27-month trial by 2.25, and discounted according to the effects discount rate of the scenario. Note that all models were run with only 20000 saved Monte Carlo simulations from 2 chains of 21000 simulations with a burn-in of 1000 and thinning of 2 (as compared to the 100,000 saved simulations from the main analysis), but adequate convergence of these models was achieved (Additional file 1). Additional file 5:
**How the cost Effectiveness of the MaiKhanda interventions compares to the Essential Health Package currently used in Malawi.**
