# Supplementary material for: Cost-effectiveness and affordability of community mobilisation through women’s groups and quality improvement in health facilities (MaiKhanda trial) in Malawi
Source: Cost Eff Resour Alloc. 2015 Jan 10;13:1. doi: 10.1186/s12962-014-0028-2 (PMC4299571; doi:10.1186/s12962-014-0028-2)
Supplement: Additional file 1: — Models, cluster-level mortality data, R source code and output. [file 12962_2014_28_MOESM1_ESM.pdf]

Additional file 1: Models, Cluster-Level Mortality Data, R Source Code and Output

This additional file contains the following:

Pages 2-3 and 4-5: the two statistical models (MKmodel_final and MKmodelFC_final) for modelling the effects of the community and facility interventions and community and facility interventions combined – see the lines they are referred to in the R Source Code.

Page 6 and Page 7: the two cluster-level mortality datasets, the first, MK.csv, for the community and facility interventions, the two-by-two arm trial [hf denotes cluster (health facility) number; fi = facility intervention; ci = community intervention; urb = urban (1) or rural (0); llw and sal are variables for the two districts the sample was also stratified by; mvital = maternal deaths; bvital = baby deaths (stillbirth or neonatal death); obs = the total number of observations in each cluster]; the second, MKFC.csv, for the combined intervention, the one-arm trial [variables as before; fici = combined facility and community intervention] – see the lines they are referred to in the R Source Code.

Pages 8-10 and 11-21: R Source Code for the main analysis, and the future scenario analysis, respectively.

Pages 22-27, 28-33 and 34-48: R Output for the main analysis, the local life expectancy sensitivity analysis [note that for this analysis the only difference in the R Source file from the main analysis – see pages 8-10 - is that 86.0 and 53.27 are replaced by 45 and 28.1, respectively], and the future scale-up scenarios analysis, respectively.

```
# MKmodel_final

model {
                 for (i in 1:length(hf)) {
                    # combined baby deaths
                 bdeath[i] ~ dbin(b[i],obs[i])
                 logit(b[i]) <- alpha0_b + fac[1]*fi[i] + com[1]*ci[i]  + strata2[1]*llw[i] +
strata3[1]*sal[i] + urban[1]*urb[i]
                    # Maternal deaths
                 mvital[i] ~ dbin(m[i],obs[i])
                 logit(m[i]) <- alpha0_m + fac[2]*fi[i] + com[2]*ci[i]  + strata2[2]*llw[i] +
strata3[2]*sal[i] + urban[2]*urb[i]
                 }
        #Blocking of coefficients (Priors)
        fac[1:2] ~ dmnorm(mu[1:2],P[1:2,1:2])
        com[1:2] ~ dmnorm(mu[1:2],P[1:2,1:2])
        strata2[1:2] ~ dmnorm(mu[1:2],P[1:2,1:2])
        strata3[1:2] ~ dmnorm(mu[1:2],P[1:2,1:2])
        urban[1:2] ~ dmnorm(mu[1:2],P[1:2,1:2])

        # Priors
        alpha0_b ~ dnorm(0.0, 1.0E-6)
        alpha0_m ~ dnorm(0.0, 1.0E-6)


#cost-effectiveness

pop <- 108000   # population of babies and mothers in 2 arms (CI, No CI, FI, no FI) in 2.25
years (1.2m * CBR of 0.04 * 27/12)

bmr_nc <- 0.059443785   # baby mortality rate in No CI arm: 219 SB + 232 NND out of 7587 births
bmr_nf <- 0.061760305   # baby mortality rate in No FI arm: 237 SB + 217 NND out of 7351 births
mmr_nc <- 0.0038223277   # mother mortality rate in No CI arm: 29 MD out of 7587 births
mmr_nf <- 0.0039450415   # mother mortality rate in No FI arm: 29 MD out of 7351 births

bm_nc ~ dbin(bmr_nc,pop)   # baby mortality in No CI arm
bm_nf ~ dbin(bmr_nf,pop)   # baby mortality in No FI arm
mm_nc ~ dbin(mmr_nc,pop)   # mother mortality in No CI arm
mm_nf ~ dbin(mmr_nf,pop)   # mother mortality in No FI arm

bs_nc <-  pop - bm_nc   # baby survivors in No CI arm
bs_nf <-  pop - bm_nf   # baby survivors in No FI arm
ms_nc <-  pop - mm_nc   # mother survivors in No CI arm
ms_nf <-  pop - mm_nf   # mother survivors in No CI arm

ob_nc  <- bm_nc / bs_nc   # odds of baby death in No CI areas
ob_nf  <- bm_nf / bs_nf   # odds of baby death in No FI areas
om_nc  <- mm_nc / ms_nc   # odds of mother death in No CI areas
om_nf  <- mm_nf / ms_nf   # odds of mother death in No FI areas

orb_c <- exp(com[1])   # Odds Ratio of CI on BMR
orb_f <- exp(fac[1])   # Odds Ratio of FI on BMR
orm_c <- exp(com[2])   # Odds Ratio of CI on MMR
orm_f <- exp(fac[2])   # Odds Ratio of FI on MMR

ob_c <- ob_nc * orb_c  # odds of baby death in CI arm (odds in No CI arms * Odds Ratio of CI on
BMR)
ob_f <- ob_nf * orb_f  # odds of baby death in FI arm (odds in No FI arms * Odds Ratio of FI on
BMR)
om_c <- om_nc * orm_c  # odds of mother death in CI arm (odds in No CI * Odds Ratio of CI on
MMR)
om_f <- om_nf * orm_f  # odds of mother death in FI arm (odds in No FI * Odds Ratio of FI on
MMR)

ob_c1 <- ob_c + 1   # odds of baby death in CI areas + 1 (for calculation of survivors in
intervention arm)
ob_f1 <- ob_f + 1   # odds of baby death in FI areas + 1 (for calculation of survivors in
intervention arm)
om_c1 <- om_c + 1   # odds of mother death in CI areas + 1 (for calculation of survivors in
intervention arm)
om_f1 <- om_f + 1   # odds of mother death in FI areas + 1 (for calculation of survivors in
intervention arm)

bs_c <- pop / ob_c1   # baby survivors in CI arm
bs_f <- pop / ob_f1   # baby survivors in FI arm
ms_c <- pop / om_c1   # mother survivors in CI arm
```

```
ms_f <- pop / om_f1   # mother survivors in FI arm

bm_c <- pop - bs_c   # baby deaths in CI arm
bm_f <- pop - bs_f   # baby deaths in FI arm
mm_c <- pop - ms_c   # mother deaths in CI arm
mm_f <- pop - ms_f   # mother deaths in FI arm

daly_b <- 86.0   # Disability Adjusted Life Years (DALYs) per baby death
daly_m <- 53.27   # Disability Adjusted Life Years (DALYs) per mother death

lb_c <-  bm_nc - bm_c   # lives of babies saved (deaths averted) by CI (difference in mortality
between No CI and CI arms)
db_c <- lb_c * daly_b   # DALYs of babies averted by CI

lm_c <-  mm_nc - mm_c   # lives of mothers saved (deaths averted) by CI (difference in
mortality between No CI and CI arms)
dm_c <- lm_c * daly_m   # DALYs of mothers averted by CI
d_c <- db_c + dm_c   # total DALYs (mothers and babies) averted by CI

lb_f <-  bm_nf - bm_f   # lives of babies saved (deaths averted) by FI (difference in mortality
between No FI and FI arms)
db_f <- lb_f * daly_b   # DALYs of babies averted by FI

lm_f <-  mm_nf - mm_f   # lives of mothers saved (deaths averted) by FI (difference in
mortality between No FI and FI arms)
dm_f <- lm_f * daly_m   # DALYs of mothers averted by FI
d_f <- db_f + dm_f   # total DALYs (mothers and babies) averted by FI

        }
```

```
# MKmodelFC_final

model {
                for (i in 1:length(hf)) {
                   # combined baby deaths
                bdeath[i] ~ dbin(b[i],obs[i])
                logit(b[i]) <- alpha0_b + fc[1]*fici[i]  + strata2[1]*llw[i] +
strata3[1]*sal[i] + urban[1]*urb[i]
                   # Maternal deaths
                mvital[i] ~ dbin(m[i],obs[i])
                logit(m[i]) <- alpha0_m + fc[2]*fici[i]  + strata2[2]*llw[i] +
strata3[2]*sal[i] + urban[2]*urb[i]
                }
        #Blocking of coefficients
        fc[1:2] ~ dmnorm(mu[1:2],P[1:2,1:2])
        strata2[1:2] ~ dmnorm(mu[1:2],P[1:2,1:2])
        strata3[1:2] ~ dmnorm(mu[1:2],P[1:2,1:2])
        urban[1:2] ~ dmnorm(mu[1:2],P[1:2,1:2])

        # Priors
        alpha0_b ~ dnorm(0.0, 1.0E-6)
        alpha0_m ~ dnorm(0.0, 1.0E-6)


#cost-effectiveness

pop <- 54000   # population of babies and mothers in 1 control arm in 2.25 years (600,000 * CBR
of 0.04 * 27/12)

bmr_con <- 0.05809575   # baby mortality rate in control arm: 105 SB + 111 NND out of 3718
births
mmr_con <- 0.0040344271   # mother mortality rate in control arm: 15 MD out of 3718 births

bm_con ~ dbin(bmr_con,pop)   # baby mortality in control arm
mm_con ~ dbin(mmr_con,pop)   # mother mortality in control arm

bs_con <-  pop - bm_con   # baby survivors in control arm
ms_con <-  pop - mm_con   # mother survivors in control arm

ob_con  <- bm_con / bs_con   # odds of baby death in control arm
om_con  <- mm_con / ms_con   # odds of mother death in control arm

orb_fc <- exp(fc[1])   # Odds Ratio of FICI on BMR
orm_fc <- exp(fc[2])   # Odds Ratio of FICI on MMR

ob_fc <- ob_con * orb_fc  # odds of baby death in FICI arm (odds in control arm * Odds Ratio of
FICI on BMR)
om_fc <- om_con * orm_fc  # odds of mother death in FICI arm (odds in control arm * Odds Ratio
of FICI on MMR)

ob_fc1 <- ob_fc + 1   # odds of baby death in FICI arm + 1 (for calculation of survivors in
intervention arm)
om_fc1 <- om_fc + 1   # odds of mother death in FICI arm + 1 (for calculation of survivors in
intervention arm)

bs_fc <- pop / ob_fc1   # baby survivors in FICI arm
ms_fc <- pop / om_fc1   # mother survivors in FICI arm

bm_fc <- pop - bs_fc   # baby deaths in FICI arm
mm_fc <- pop - ms_fc   # mother deaths in FICI arm

daly_b <- 86.0   # Disability Adjusted Life Years (DALYs) per baby death
daly_m <- 53.27   # Disability Adjusted Life Years (DALYs) per mother death

lb_fc <-  bm_con - bm_fc   # lives of babies saved (deaths averted) by FICI (difference in
mortality between control and FICI arms)
db_fc <- lb_fc * daly_b   # DALYs of babies averted by FICI

lm_fc <-  mm_con - mm_fc   # lives of mothers saved (deaths averted) by FICI (difference in
mortality between control and FICI arms)
dm_fc <- lm_fc * daly_m   # DALYs of mothers averted by FICI
d_fc <- db_fc + dm_fc   # total DALYs (mothers and babies) averted by FICI

#For Table 1 credible intervals for two-arm FICI:
lb_fc_ <-  lb_fc * 2
db_fc_ <- db_fc * 2
```

```
lm_fc_ <-  lm_fc * 2
dm_fc_ <- dm_fc * 2
d_fc_ <- d_fc * 2
        }
```

MK.csv

hf	fi	ci	urb	llw	sal	mvital	bdeath	obs
1	1	1	0	0	1	2	24	335
2	0	0	1	1	0	1	8	131
3	1	0	1	1	0	0	8	224
4	1	0	0	0	0	2	20	302
5	1	0	0	1	0	0	7	159
6	0	0	0	1	0	0	18	258
7	1	1	0	1	0	0	13	315
8	0	0	0	1	0	2	4	77
9	1	0	0	0	1	1	29	463
10	1	1	0	0	1	1	33	524
11	1	0	0	0	1	1	24	373
12	1	0	0	1	0	1	34	359
13	0	1	0	1	0	1	28	387
14	1	1	0	1	0	2	24	425
15	0	0	0	0	0	0	32	397
16	0	0	0	1	0	1	18	215
17	0	1	0	1	0	1	22	407
18	0	0	0	1	0	0	11	123
19	1	0	0	0	0	3	24	526
20	0	0	0	0	0	0	16	226
21	1	0	0	0	0	0	36	456
22	0	1	0	0	1	1	43	691
23	0	0	0	1	0	0	12	309
24	0	0	1	1	0	0	5	132
25	1	1	0	0	0	0	29	352
26	0	1	0	0	0	0	14	200
27	1	1	0	0	1	1	8	268
28	1	1	0	1	0	1	25	577
29	1	0	0	0	0	3	17	503
30	1	0	0	1	0	0	34	474
31	0	0	0	0	1	3	31	582
32	0	0	0	0	1	1	34	526
33	1	1	0	1	0	1	14	215
34	1	0	0	1	0	0	26	421
35	1	0	0	1	0	1	8	262
36	1	1	0	1	0	0	6	188
37	0	1	0	1	0	0	8	249
38	0	1	0	1	0	1	23	407
39	1	1	0	1	0	0	14	204
40	0	1	0	1	0	0	10	187
41	0	1	0	0	1	1	32	515
42	0	0	0	1	0	0	20	268
43	0	0	0	0	0	0	19	221
44	0	1	0	0	0	2	11	284
45	0	0	0	1	0	0	16	371
46	1	1	0	0	0	3	19	389
47	1	1	0	0	0	0	20	358
48	0	0	0	1	0	0	20	323
49	0	1	0	1	0	0	7	213
50	1	1	0	1	0	0	19	423
51	1	0	0	0	1	2	25	490
52	1	1	0	1	0	0	20	332
53	0	1	0	0	0	3	24	529
54	0	1	0	1	0	0	10	148
55	0	1	0	1	0	0	7	235
56	1	1	0	0	0	0	12	345
57	0	0	0	0	1	1	7	146
58	0	1	0	0	0	1	24	298
59	0	1	0	0	1	0	17	335
60	1	0	0	1	0	1	24	327
61	0	0	0	0	0	1	37	610

MKFC.csv

strata	arm	hf	fici	urb	llw	sal	mvital	bdeath	obs
3	3	1	1	0	0	1	2	24	335
2	0	2	0	1	1	0	1	8	131
2	0	3	0	0	1	0	0	18	258
2	3	4	1	0	1	0	0	13	315
2	0	5	0	0	1	0	2	4	77
3	3	6	1	0	0	1	1	33	524
2	3	7	1	0	1	0	2	24	425
1	0	8	0	0	0	0	0	32	397
2	0	9	0	0	1	0	1	18	215
2	0	10	0	0	1	0	0	11	123
1	0	11	0	0	0	0	0	16	226
2	0	12	0	0	1	0	0	12	309
2	0	13	0	1	1	0	0	5	132
1	3	14	1	0	0	0	0	29	352
3	3	15	1	0	0	1	1	8	268
2	3	16	1	0	1	0	1	25	577
3	0	17	0	0	0	1	3	31	582
3	0	18	0	0	0	1	1	34	526
2	3	19	1	0	1	0	1	14	215
2	3	20	1	0	1	0	0	6	188
2	3	21	1	0	1	0	0	14	204
2	0	22	0	0	1	0	0	20	268
1	0	23	0	0	0	0	0	19	221
2	0	24	0	0	1	0	0	16	371
1	3	25	1	0	0	0	3	19	389
1	3	26	1	0	0	0	0	20	358
2	0	27	0	0	1	0	0	20	323
2	3	28	1	0	1	0	0	19	423
2	3	29	1	0	1	0	0	20	332
1	3	30	1	0	0	0	0	12	345
3	0	31	0	0	0	1	1	7	146
1	0	32	0	0	0	0	1	37	610

```
setwd("/Users/timothycolbourn/Documents/Papers/MaiKhanda CEA paper/JAGS revised")  #set working directory for
 saving outputs
sink(file="MaiKhanda_final_log_.txt",split=TRUE)
# MaiKhanda Economic Evaluation v4 in R and JAGS Tim Colbourn 2nd Dec 2014
install.packages("R2jags")
library(R2jags)
install.packages("BCEA")
library(BCEA)


    # Community Intervention and Facility Intervention model

mk <- read.csv('MK.csv')  # data exported from Stata using 'MK_WinBUGS_final_cluster.do' and also stored in
 'MKcluster.txt'

h <- 100
mu <- c(0,0)
P <- (1/h^2)*diag(2)

data <- list(mu=mu,P=P,hf=mk$hf,fi=mk$fi,ci=mk$ci,urb=mk$urb,llw=mk$llw,sal=mk$sal,mvital=mk$mvital,bdeath=mk
 $bdeath,obs=mk$obs)

filein <- "/Users/timothycolbourn/Documents/Papers/MaiKhanda CEA paper/JAGS revised/MKmodel_final.txt"  #filepath
 to text file containing model

inits <- function(){
    list(alpha0_b=rnorm(1), alpha0_m=rnorm(1), strata2=rnorm(2,0,1), strata3=rnorm(2,0,1), urban=rnorm(2,0,1),
 fac=rnorm(2,0,1), com=rnorm(2,0,1))
    }  # initial values of paramters

params <-
 c("bs_nc","bs_c","ms_nc","ms_c","bs_nf","bs_f","ms_nf","ms_f","orb_c","orm_c","orb_f","orm_f","lb_c","db_c","lm_c"
 ,"dm_c","lb_f","db_f","lm_f","dm_f","d_c","d_f")  #paramters to monitor

model <- jags(data,inits,params,model.file=filein,n.chains=2,n.iter=510000,n.burnin=10000,n.thin=10,DIC=TRUE)

print(model,digits=3,intervals=c(0.025,0.500,0.975))

m <- as.mcmc(model)  #converts the object model to an MCMC structure, for plotting and diagnostics
raftery.diag(m)  #Raftery-Lewis diagnostic estimates of (for each chain[[1]],[[2]]..) number of iterations
 required to estimate 2.5th quantile (=q) of posterior dist, with accuracy (tolerance) r of +/-0.005 and
 probability s of 0.95 (defaults)
summary(m)  #MCMC error and other indications of convergence. MCMC error is how close posterior SD is to Naive SE.
 Time-series SE adjusts Naive SE for autocorrelation; lower values = better convergence

        #Health Economic analysis, including EVPPI (p.145-151) - via BCEA package loaded earlier
attach.jags(model)
            # Community Intervention (CI) vs Do Nothing
ec <- cc <- matrix(NA,model$BUGSoutput$n.sims,2)
ec <- matrix(c((bs_nc*86.0 + ms_nc*53.27),(bs_c*86.0 + ms_c*53.27)),model$BUGSoutput$n.sims,2)   # effects = baby
 (times 86.0 DALYs each) survivors + mother survivors (times 53.27 DALYs each) in No CI, baby (times 86.0 DALYs
 each) survivors + mother survivors (times 53.27 DALYs each) in CI
cc <- matrix(c(rep(0,model$BUGSoutput$n.sims),rep(5348791,model$BUGSoutput$n.sims)),model$BUGSoutput$n.sims,2)   #
 costs = cost of do nothing, cost of community intervention
treatsc <- c("do nothing","Community Intervention")
ci <- bcea(e=ec,c=cc,ref=2,interventions=treatsc,Kmax=2500) # Max cost-effectivness threshold is over 3 times
 GDPpc per DALY averted
layout(matrix(1:4,2,2)) #so the four health economic analysis plots from the following commands are on one page
ceplane.plot(ci,comparison=1,wtp=779.8)   # Cost-effectiveness plane - Willingness to Pay (WTP) threshold is
 $779.8 GDPpc (per DALY averted)
eib.plot(ci)    # Expected Incremental Benefit
ceac.plot(ci)   # Cost-effectiveness Acceptability Curve (prob cost-effective by WTP)
evi.plot(ci)   # Expected value of information by willingness to pay
quartz.save("Figure1.pdf", type="pdf")
summary(ci,wtp=780) # Increments of 5 are only allowed in this command therefore 779.8 is not allowed
            # Facility Intervention (FI) vs Do Nothing
ef <- cf <- matrix(NA,model$BUGSoutput$n.sims,2)
ef <- matrix(c((bs_nf*86.0 + ms_nf*53.27),(bs_f*86.0 + ms_f*53.27)),model$BUGSoutput$n.sims,2)   # effects = baby
 (times 86.0 DALYs each) survivors + mother survivors (times 53.27 DALYs each) in No FI, baby (times 86.0 DALYs
 each) survivors + mother survivors (times 53.27 DALYs each) in FI
cf <- matrix(c(rep(0,model$BUGSoutput$n.sims),rep(5592212,model$BUGSoutput$n.sims)),model$BUGSoutput$n.sims,2)   #
 costs = cost of do nothing, cost of community intervention
treatsf <- c("do nothing","Facility Intervention")
fi <- bcea(e=ef,c=cf,ref=2,interventions=treatsf,Kmax=2500)
layout(matrix(1:4,2,2)) #so the four health economic analysis plots from the following commands are on one page
ceplane.plot(fi,comparison=1,wtp=779.8)   # Cost-effectiveness plane
eib.plot(fi)    # Expected Incremental Benefit
ceac.plot(fi)   # Cost-effectiveness Acceptability Curve (prob cost-effective by WTP)
evi.plot(fi)   # Expected value of information by willingness to pay
```

```
quartz.save("Figure2.pdf", type="pdf")
summary(fi,wtp=780) # Increments of 5 are only allowed in this command therefore 779.8 is not allowed
            # Community Intervention (CI) vs Facility Intervention (FI)
e2 <- c2 <- matrix(NA,model$BUGSoutput$n.sims,2)
e2 <- matrix(c((bs_c*86.0 + ms_c*53.27),(bs_f*86.0 + ms_f*53.27)),model$BUGSoutput$n.sims,2)   # effects = baby
 (times 86.0 DALYs each) survivors + mother survivors (times 53.27 DALYs each) in CI, baby (times 86.0 DALYs each)
 survivors + mother survivors (times 53.27 DALYs each) in FI
c2 <- matrix(c(rep(5348791,model$BUGSoutput$n.sims),rep(5592212,model$BUGSoutput$n.sims)),model$BUGSoutput$n.sims,
 2)   # costs = cost of community intervention, cost of facility intervention
treatscvf <- c("Community Intervention","Facility Intervention")
cvf <- bcea(e=e2,c=c2,ref=1,interventions=treatscvf,Kmax=2500)
layout(matrix(1:4,2,2)) #so the four health economic analysis plots from the following commands are on one page
ceplane.plot(cvf,comparison=1,wtp=779.8)   # Cost-effectiveness plane
eib.plot(cvf)    # Expected Incremental Benefit
ceac.plot(cvf)   # Cost-effectiveness Acceptability Curve (prob cost-effective by WTP)
evi.plot(cvf)   # Expected value of information by willingness to pay
quartz.save("plots_CvF.pdf", type="pdf")
summary(cvf,wtp=780) # Increments of 5 are only allowed in this command therefore 779.8 is not allowed


    # combined Facility Intervention & Community Intervention (FICI) model
mkfc <- read.csv('MKFC.csv')  # data exported from Stata using 'MK_WinBUGS_final_cluster_FC.do' and also stored in
 'MKclusterFC.txt'

data2 <- list(mu=mu,P=P,hf=mkfc$hf,fici=mkfc$fici,urb=mkfc$urb,llw=mkfc$llw,sal=mkfc$sal,mvital=mkfc
 $mvital,bdeath=mkfc$bdeath,obs=mkfc$obs)

filein2 <- "/Users/timothycolbourn/Documents/Papers/MaiKhanda CEA paper/JAGS revised/MKmodelFC_final.txt"
 #filepath to text file containing model

inits2 <- function(){
        list(alpha0_b=rnorm(1), alpha0_m=rnorm(1), strata2=rnorm(2,0,1), strata3=rnorm(2,0,1), urban=rnorm(2,0,1),
 fc=rnorm(2,0,1))
    }  # initial values of paramters

params2 <-
 c("bs_con","bs_fc","ms_con","ms_fc","orb_fc","orm_fc","lb_fc","db_fc","lm_fc","dm_fc","d_fc","lb_fc_","db_fc_","lm
 _fc_","dm_fc_","d_fc_")  #paramters to monitor

model2 <- jags(data2,inits2,params2,model.file=filein2,n.chains=2,n.iter=510000,n.burnin=10000,n.thin=10,DIC=TRUE)

print(model2,digits=3,intervals=c(0.025,0.500,0.975))  #produces customised output

m2 <- as.mcmc(model2)  #converts the object model to an MCMC structure, for plotting and diagnostics
raftery.diag(m2)  #Raftery-Lewis diagnostic estimates of (for each chain[[1]],[[2]]..) number of iterations
 required to estimate 2.5th quantile (=q) of posterior dist, with accuracy (tolerance) r of +/-0.005 and
 probability s of 0.95 (defaults)
summary(m2)  #MCMC error and other indications of convergence. MCMC error is how close posterior SD is to Naive
 SE. Time-series SE adjusts Naive SE for autocorrelation; lower values = better convergence

    #Health Economic analysis, including EVPPI (p.145-151) - via BCEA package loaded earlier

        # combined Facility Intervention & Community Intervention (FICI) vs Do Nothing
s_c <- bs_c*86.0 + ms_c*53.27   # need these estimates from the above model (for the CI v FI v FICI comparison
 below) before we attach the estimates from the second model (see line 121 below). Note the number of baby and
 mother survivors are each already multiplied by their respective DALYs averted here
s_f <- bs_f*86.0 + ms_f*53.27

attach.jags(model2)
efc <- cfc <- matrix(NA,model2$BUGSoutput$n.sims,2)
efc <- matrix(c((bs_con*86.0 + ms_con*53.27),(bs_fc*86.0 + ms_fc*53.27)),model2$BUGSoutput$n.sims,2)   # effects =
 baby (times 86.0 DALYs each) survivors + mother survivors (times 53.27 DALYs each) in control, baby (times 86.0
 DALYs each) survivors + mother survivors (times 53.27 DALYs each) in FICI
cfc <- matrix(c(rep(0,model2$BUGSoutput$n.sims),rep(5470501,model2$BUGSoutput$n.sims)),model$BUGSoutput$n.sims,2)
 # costs = cost of do nothing, cost of combined facility and community intervention
treatsfc <- c("do nothing","Facility & Community Interventions")
fc <- bcea(e=efc,c=cfc,ref=2,interventions=treatsfc,Kmax=2500)
layout(matrix(1:4,2,2)) #so the four health economic analysis plots from the following commands are on one page
ceplane.plot(fc,comparison=1,wtp=779.8)   # Cost-effectiveness plane
eib.plot(fc)    # Expected Incremental Benefit
ceac.plot(fc)   # Cost-effectiveness Acceptability Curve (prob cost-effective by WTP)
evi.plot(fc)   # Expected value of information by willingness to pay
quartz.save("Figure3.pdf", type="pdf")
summary(fc,wtp=780) # Increments of 5 are only allowed in this command therefore 779.8 is not allowed
        # Community Intervention (CI) vs Facility Intervention (FI) vs. combined FICI
e3 <- c3 <- matrix(NA,model$BUGSoutput$n.sims,3)
e3 <- matrix(c(s_c,s_f,(bs_fc*86.0*2 + ms_fc*53.27*2)),model$BUGSoutput$n.sims,3)   # effects = baby+mother
 survivors (*DALYs, see line 103) in CI, baby+mother survivors (*DALYs, see line 104) in FI, baby+mother survivors
 in FICI (*DALYs) multiplied by two so the population size is the same as CI, and FI
```

```
c3 <- matrix(c(rep(5348791,model$BUGSoutput$n.sims),rep(5592212,model$BUGSoutput$n.sims),rep(5470501*2,model
$BUGSoutput$n.sims)),model$BUGSoutput$n.sims,3)   # costs = cost of community intervention, cost of facility
 intervention, cost of FICI multiplied by two so the population size is the same as CI, and FI
treats3 <- c("Community Intervention","Facility Intervention","FICI")
cvfvfc <- bcea(e=e3,c=c3,ref=1,interventions=treats3,Kmax=2500)
layout(matrix(1:4,2,2)) #so the four health economic analysis plots from the following commands are on one page
ceplane.plot(cvfvfc,comparison=2,wtp=779.8)   # Cost-effectiveness plane
eib.plot(cvfvfc)    # Expected Incremental Benefit
ceac.plot(cvfvfc)   # Cost-effectiveness Acceptability Curve (prob cost-effective by WTP)
evi.plot(cvfvfc)   # Expected value of information by willingness to pay
quartz.save("Figure4.pdf", type="pdf")
summary(cvfvfc,wtp=780) # Increments of 5 are only allowed in this command therefore 779.8 is not allowed
            # Facility Intervention (FI) vs Community Intervention (CI) vs combined FICI
fvcvfc <- bcea(e=e3,c=c3,ref=2,interventions=treats3,Kmax=2500)
layout(matrix(1:4,2,2)) #so the four health economic analysis plots from the following commands are on one page
ceplane.plot(fvcvfc,comparison=2,wtp=779.8)   # Cost-effectiveness plane
eib.plot(fvcvfc)    # Expected Incremental Benefit
ceac.plot(fvcvfc)   # Cost-effectiveness Acceptability Curve (prob cost-effective by WTP)
evi.plot(fvcvfc)   # Expected value of information by willingness to pay
quartz.save("plots_FvCvFC.pdf", type="pdf")
summary(fvcvfc,wtp=780) # Increments of 5 are only allowed in this command therefore 779.8 is not allowed
            # combined FICI vs Facility Intervention (FI) vs Community Intervention (CI)
fcvfvc <- bcea(e=e3,c=c3,ref=3,interventions=treats3,Kmax=2500)
layout(matrix(1:4,2,2)) #so the four health economic analysis plots from the following commands are on one page
ceplane.plot(fcvfvc,comparison=1,wtp=779.8)   # Cost-effectiveness plane
eib.plot(fcvfvc)    # Expected Incremental Benefit
ceac.plot(fcvfvc)   # Cost-effectiveness Acceptability Curve (prob cost-effective by WTP)
evi.plot(fcvfvc)   # Expected value of information by willingness to pay
quartz.save("plots_FCvFvC.pdf", type="pdf")
summary(fcvfvc,wtp=780) # Increments of 5 are only allowed in this command therefore 779.8 is not allowed

sink()
```

```r
setwd("/Users/timothycolbourn/Documents/MaiKhanda CEA paper/JAGS")  #set working directory for saving outputs
sink(file="MaiKhanda_future_final_log.txt",split=TRUE)
# MaiKhanda Economic Evaluation v4 in R and JAGS Tim Colbourn 2nd Dec 2014
#install.packages("R2jags")
library(R2jags)
#install.packages("BCEA")
library(BCEA)


    # Community Intervention and Facility Intervention model

mk <- read.csv('MK.csv')  # data exported from Stata using 'MK_WinBUGS_final_cluster.do' and also stored in
 'MKcluster.txt'

h <- 100
mu <- c(0,0)
P <- (1/h^2)*diag(2)

data <- list(mu=mu,P=P,hf=mk$hf,fi=mk$fi,ci=mk$ci,urb=mk$urb,llw=mk$llw,sal=mk$sal,mvital=mk$mvital,bdeath=mk
 $bdeath,obs=mk$obs)

filein <- "/Users/timothycolbourn/Documents/MaiKhanda CEA paper/JAGS/MKmodel.txt"  #filepath to text file
 containing model

inits <- function(){
    list(alpha0_b=rnorm(1), alpha0_m=rnorm(1), strata2=rnorm(2,0,1), strata3=rnorm(2,0,1), urban=rnorm(2,0,1),
 fac=rnorm(2,0,1), com=rnorm(2,0,1))
    }  # initial values of paramters

params <-
 c("bs_nc","bs_c","ms_nc","ms_c","bs_nf","bs_f","ms_nf","ms_f","orb_c","orm_c","orb_f","orm_f","lb_c","db_c","lm_c"
 ,"dm_c","lb_f","db_f","lm_f","dm_f","d_c","d_f")  #paramters to monitor

model <- jags(data,inits,params,model.file=filein,n.chains=2,n.iter=21000,n.burnin=1000,n.thin=2,DIC=TRUE)

print(model,digits=3,intervals=c(0.025,0.500,0.975))

m <- as.mcmc(model)  #converts the object model to an MCMC structure, for plotting and diagnostics
raftery.diag(m)  #Raftery-Lewis diagnostic estimates of (for each chain[[1]],[[2]]..) number of iterations
 required to estimate 2.5th quantile (=q) of posterior dist, with accuracy (tolerance) r of +/-0.005 and
 probability s of 0.95 (defaults)
summary(m)  #MCMC error and other indications of convergence. MCMC error is how close posterior SD is to Naive SE.
 Time-series SE adjusts Naive SE for autocorrelation; lower values = better convergence

        #Health Economic analysis, including EVPPI (p.145-151) - via BCEA package loaded earlier
attach.jags(model)
            # Community Intervention (CI) vs Do Nothing
ec <- cc <- matrix(NA,model$BUGSoutput$n.sims,2)
ec <- matrix(c((bs_nc*86.0 + ms_nc*53.27),(bs_c*86.0 + ms_c*53.27)),model$BUGSoutput$n.sims,2)   # effects = baby
 (times 86.0 DALYs each) survivors + mother survivors (times 53.27 DALYs each) in No CI, baby (times 86.0 DALYs
 each) survivors + mother survivors (times 53.27 DALYs each) in CI
cc <- matrix(c(rep(0,model$BUGSoutput$n.sims),rep(5348791,model$BUGSoutput$n.sims)),model$BUGSoutput$n.sims,2)   #
 costs = cost of do nothing, cost of community intervention
treatsc <- c("do nothing","Community Intervention")
ci <- bcea(e=ec,c=cc,ref=2,interventions=treatsc,Kmax=2500) # Max cost-effectivness threshold is over 3 times
 GDPpc per DALY averted
layout(matrix(1:4,2,2)) #so the four health economic analysis plots from the following commands are on one page
ceplane.plot(ci,comparison=1,wtp=779.8)   # Cost-effectiveness plane - Willingness to Pay (WTP) threshold is
 $779.8 GDPpc (per DALY averted)
eib.plot(ci)    # Expected Incremental Benefit
ceac.plot(ci)   # Cost-effectiveness Acceptability Curve (prob cost-effective by WTP)
evi.plot(ci)   # Expected value of information by willingness to pay
quartz.save("plots_C_.pdf", type="pdf")
summary(ci,wtp=780) # Increments of 5 are only allowed in this command therefore 779.8 is not allowed
            # Facility Intervention (FI) vs Do Nothing
ef <- cf <- matrix(NA,model$BUGSoutput$n.sims,2)
ef <- matrix(c((bs_nf*86.0 + ms_nf*53.27),(bs_f*86.0 + ms_f*53.27)),model$BUGSoutput$n.sims,2)   # effects = baby
 (times 86.0 DALYs each) survivors + mother survivors (times 53.27 DALYs each) in No FI, baby (times 86.0 DALYs
 each) survivors + mother survivors (times 53.27 DALYs each) in FI
cf <- matrix(c(rep(0,model$BUGSoutput$n.sims),rep(5592212,model$BUGSoutput$n.sims)),model$BUGSoutput$n.sims,2)   #
 costs = cost of do nothing, cost of community intervention
treatsf <- c("do nothing","Facility Intervention")
fi <- bcea(e=ef,c=cf,ref=2,interventions=treatsf,Kmax=2500)
layout(matrix(1:4,2,2)) #so the four health economic analysis plots from the following commands are on one page
ceplane.plot(fi,comparison=1,wtp=779.8)   # Cost-effectiveness plane
eib.plot(fi)    # Expected Incremental Benefit
ceac.plot(fi)   # Cost-effectiveness Acceptability Curve (prob cost-effective by WTP)
evi.plot(fi)   # Expected value of information by willingness to pay
quartz.save("plots_F_.pdf", type="pdf")
```

```
summary(fi,wtp=780) # Increments of 5 are only allowed in this command therefore 779.8 is not allowed
            # Community Intervention (CI) vs Facility Intervention (FI)
e2 <- c2 <- matrix(NA,model$BUGSoutput$n.sims,2)
e2 <- matrix(c((bs_c*86.0 + ms_c*53.27),(bs_f*86.0 + ms_f*53.27)),model$BUGSoutput$n.sims,2)   # effects = baby
 (times 86.0 DALYs each) survivors + mother survivors (times 53.27 DALYs each) in CI, baby (times 86.0 DALYs each)
 survivors + mother survivors (times 53.27 DALYs each) in FI
c2 <- matrix(c(rep(5348791,model$BUGSoutput$n.sims),rep(5592212,model$BUGSoutput$n.sims)),model$BUGSoutput$n.sims,
 2)   # costs = cost of community intervention, cost of facility intervention
treatscvf <- c("Community Intervention","Facility Intervention")
cvf <- bcea(e=e2,c=c2,ref=1,interventions=treatscvf,Kmax=2500)
layout(matrix(1:4,2,2)) #so the four health economic analysis plots from the following commands are on one page
ceplane.plot(cvf,comparison=1,wtp=779.8)   # Cost-effectiveness plane
eib.plot(cvf)    # Expected Incremental Benefit
ceac.plot(cvf)   # Cost-effectiveness Acceptability Curve (prob cost-effective by WTP)
evi.plot(cvf)   # Expected value of information by willingness to pay
quartz.save("plots_CvF_.pdf", type="pdf")
summary(cvf,wtp=780) # Increments of 5 are only allowed in this command therefore 779.8 is not allowed


    # combined Facility Intervention & Community Intervention (FICI) model
mkfc <- read.csv('MKFC.csv')  # data exported from Stata using 'MK_WinBUGS_final_cluster_FC.do' and also stored in
 'MKclusterFC.txt'

data2 <- list(mu=mu,P=P,hf=mkfc$hf,fici=mkfc$fici,urb=mkfc$urb,llw=mkfc$llw,sal=mkfc$sal,mvital=mkfc
 $mvital,bdeath=mkfc$bdeath,obs=mkfc$obs)

filein2 <- "/Users/timothycolbourn/Documents/MaiKhanda CEA paper/JAGS/MKmodelFC.txt"  #filepath to text file
 containing model

inits2 <- function(){
        list(alpha0_b=rnorm(1), alpha0_m=rnorm(1), strata2=rnorm(2,0,1), strata3=rnorm(2,0,1), urban=rnorm(2,0,1),
 fc=rnorm(2,0,1))
    }  # initial values of paramters

params2 <- c("bs_con","bs_fc","ms_con","ms_fc","orb_fc","orm_fc","lb_fc","db_fc","lm_fc","dm_fc","d_fc")
 #paramters to monitor

model2 <- jags(data2,inits2,params2,model.file=filein2,n.chains=2,n.iter=21000,n.burnin=1000,n.thin=2,DIC=TRUE)

print(model2,digits=3,intervals=c(0.025,0.500,0.975))  #produces customised output

m2 <- as.mcmc(model2)  #converts the object model to an MCMC structure, for plotting and diagnostics
raftery.diag(m2)  #Raftery-Lewis diagnostic estimates of (for each chain[[1]],[[2]]..) number of iterations
 required to estimate 2.5th quantile (=q) of posterior dist, with accuracy (tolerance) r of +/-0.005 and
 probability s of 0.95 (defaults)
summary(m2)  #MCMC error and other indications of convergence. MCMC error is how close posterior SD is to Naive
 SE. Time-series SE adjusts Naive SE for autocorrelation; lower values = better convergence

    #Health Economic analysis, including EVPPI (p.145-151) - via BCEA package loaded earlier

        # combined Facility Intervention & Community Intervention (FICI) vs Do Nothing
s_c <- bs_c*86.0 + ms_c*53.27   # need these estimates from the above model (for the CI v FI v FICI comparison
 below) before we attach the estimates from the second model (see line 121 below). Note the number of baby and
 mother survivors are each already multiplied by their respective DALYs averted here
s_f <- bs_f*86.0 + ms_f*53.27

attach.jags(model2)
efc <- cfc <- matrix(NA,model2$BUGSoutput$n.sims,2)
efc <- matrix(c((bs_con*86.0 + ms_con*53.27),(bs_fc*86.0 + ms_fc*53.27)),model2$BUGSoutput$n.sims,2)   # effects =
 baby (times 86.0 DALYs each) survivors + mother survivors (times 53.27 DALYs each) in control, baby (times 86.0
 DALYs each) survivors + mother survivors (times 53.27 DALYs each) in FICI
cfc <- matrix(c(rep(0,model2$BUGSoutput$n.sims),rep(5470501,model2$BUGSoutput$n.sims)),model$BUGSoutput$n.sims,2)
 # costs = cost of do nothing, cost of combined facility and community intervention
treatsfc <- c("do nothing","Facility & Community Interventions")
fc <- bcea(e=efc,c=cfc,ref=2,interventions=treatsfc,Kmax=2500)
layout(matrix(1:4,2,2)) #so the four health economic analysis plots from the following commands are on one page
ceplane.plot(fc,comparison=1,wtp=779.8)   # Cost-effectiveness plane
eib.plot(fc)    # Expected Incremental Benefit
ceac.plot(fc)   # Cost-effectiveness Acceptability Curve (prob cost-effective by WTP)
evi.plot(fc)   # Expected value of information by willingness to pay
quartz.save("plots_FC_.pdf", type="pdf")
summary(fc,wtp=780) # Increments of 5 are only allowed in this command therefore 779.8 is not allowed
        # Community Intervention (CI) vs Facility Intervention (FI) vs. combined FICI
e3 <- c3 <- matrix(NA,model$BUGSoutput$n.sims,3)
e3 <- matrix(c(s_c,s_f,(bs_fc*86.0*2 + ms_fc*53.27*2)),model$BUGSoutput$n.sims,3)   # effects = baby+mother
 survivors (*DALYs, see line 103) in CI, baby+mother survivors (*DALYs, see line 104) in FI, baby+mother survivors
 in FICI (*DALYs) multiplied by two so the population size is the same as CI, and FI
c3 <- matrix(c(rep(5348791,model$BUGSoutput$n.sims),rep(5592212,model$BUGSoutput$n.sims),rep(5470501*2,model
 $BUGSoutput$n.sims)),model$BUGSoutput$n.sims,3)   # costs = cost of community intervention, cost of facility
```

```
 intervention, cost of FICI multiplied by two so the population size is the same as CI, and FI
treats3 <- c("Community Intervention","Facility Intervention","FICI")
cvfvfc <- bcea(e=e3,c=c3,ref=1,interventions=treats3,Kmax=2500)
layout(matrix(1:4,2,2)) #so the four health economic analysis plots from the following commands are on one page
ceplane.plot(cvfvfc,comparison=2,wtp=779.8)   # Cost-effectiveness plane
eib.plot(cvfvfc)    # Expected Incremental Benefit
ceac.plot(cvfvfc)   # Cost-effectiveness Acceptability Curve (prob cost-effective by WTP)
evi.plot(cvfvfc)   # Expected value of information by willingness to pay
quartz.save("plots_CvFvFC_.pdf", type="pdf")
summary(cvfvfc,wtp=780) # Increments of 5 are only allowed in this command therefore 779.8 is not allowed


    #Future scale-up 1
years <- 5   # Time Horizon: Years of scale-up projection
cdr <- 0.03   # Cost Discount Rate (per year)
edr <- 0.00  # Effect Discount Rate (per year)
cycle <- c(1:years)
scale <- (15013694*0.04*2)/(1200000*0.04*2)  # scale-up conversion factor - see Table 2 of paper - ratio of trial
 population to total population of Malawi (assume budget for intervention scales proportionally with increased
 population so that the total beneficiaries per year and total cost per year (not discounted) remains the same)

s_cm <- (s_c/2.25)*scale   # DALYs averted per year bythe Community Intervention if scaled-up to the whole of
 Malawi
fut_ec_=NA*c(1:(length(s_cm)*length(cycle)))
fut_ec__ <- fut_ec_5 <- fut_ec_4 <- fut_ec_3 <- fut_ec_2 <- fut_ec_1 <- NULL
for (a in 1:length(s_cm)) {
    for (i in 1:length(cycle)) fut_ec__[i] <-c(s_cm[a]/((1+edr)^i))
    fut_ec_5[a] <- fut_ec__[i]
}
for (a in 1:length(s_cm)) {
    for (i in 1:length(cycle)) fut_ec__[i] <-c(s_cm[a]/((1+edr)^i))
    fut_ec_4[a] <- fut_ec__[i-1]
}
for (a in 1:length(s_cm)) {
    for (i in 1:length(cycle)) fut_ec__[i] <-c(s_cm[a]/((1+edr)^i))
    fut_ec_3[a] <- fut_ec__[i-2]
}
for (a in 1:length(s_cm)) {
    for (i in 1:length(cycle)) fut_ec__[i] <-c(s_cm[a]/((1+edr)^i))
    fut_ec_2[a] <- fut_ec__[i-3]
}
for (a in 1:length(s_cm)) {
    for (i in 1:length(cycle)) fut_ec__[i] <-c(s_cm[a]/((1+edr)^i))
    fut_ec_1[a] <- fut_ec__[i-4]
}
fut_ec_ <- c(fut_ec_1,fut_ec_2,fut_ec_3,fut_ec_4,fut_ec_5)
fut_ec <- tapply( fut_ec_, (seq_along(fut_ec_)-1) %/% years, sum)   # Future effects of CI for all years in time
 horizon added together (for each of the simulations)
start_c <- 362083*scale   # Start-up costs of Community Intervention for the whole of Malawi
annual_c <- (27250+2068997)*scale   # Annual running costs of Community Intervention for the whole of Malawi
annualext_c  <- 120067*scale   # Annual external costs of Community Intervention  for the whole of Malawi
fut_cc_ <- NULL
for (i in 1:length(cycle)) fut_cc_[i] <-c((annual_c + annualext_c)/((1+cdr)^i))    # Future costs of CI for each
 year in time horizon
fut_cc <- start_c + tapply( fut_cc_, (seq_along(fut_cc_)-1) %/% years, sum)   # Future costs of CI for all years
 in time horizon added together (this is constant so needs to be replicated by the number of simulations in the
 BCEA)

s_fm <- (s_f/2.25)*scale   # DALYs averted per year by the Facility Intervention if scaled-up to the whole of
 Malawi
fut_ef_=NA*c(1:(length(s_fm)*length(cycle)))
fut_ef__ <- fut_ef_5 <- fut_ef_4 <- fut_ef_3 <- fut_ef_2 <- fut_ef_1 <- NULL
for (a in 1:length(s_fm)) {
    for (i in 1:length(cycle)) fut_ef__[i] <-c(s_fm[a]/((1+edr)^i))
    fut_ef_5[a] <- fut_ef__[i]
}
for (a in 1:length(s_fm)) {
    for (i in 1:length(cycle)) fut_ef__[i] <-c(s_fm[a]/((1+edr)^i))
    fut_ef_4[a] <- fut_ef__[i-1]
}
for (a in 1:length(s_fm)) {
    for (i in 1:length(cycle)) fut_ef__[i] <-c(s_fm[a]/((1+edr)^i))
    fut_ef_3[a] <- fut_ef__[i-2]
}
for (a in 1:length(s_fm)) {
    for (i in 1:length(cycle)) fut_ef__[i] <-c(s_fm[a]/((1+edr)^i))
    fut_ef_2[a] <- fut_ef__[i-3]
}
for (a in 1:length(s_fm)) {
```

```
    for (i in 1:length(cycle)) fut_ef__[i] <-c(s_fm[a]/((1+edr)^i))
    fut_ef_1[a] <- fut_ef__[i-4]
}
fut_ef_ <- c(fut_ef_1,fut_ef_2,fut_ef_3,fut_ef_4,fut_ef_5)
fut_ef <- tapply( fut_ef_, (seq_along(fut_ef_)-1) %/% years, sum)   # Future effects of FI for all years in time
 horizon added together, for each of the simulations
start_f <- 362860*scale   # Start-up costs of Facility Intervention for the whole of Malawi
annual_f <- (25328+2026811)*scale   # Annual running costs of Facility Intervention for the whole of Malawi
annualext_f  <- 272017*scale   # Annual external costs of Facility Intervention for the whole of Malawi
fut_cf_ <- NULL
for (i in 1:length(cycle)) fut_cf_[i] <-c((annual_f + annualext_f)/((1+cdr)^i))    # Future costs of FI for each
 year in time horizon
fut_cf <- start_f + tapply( fut_cf_, (seq_along(fut_cf_)-1) %/% years, sum)   # Future costs of FI for all years
 in time horizon added together, for each of the simulations

s_fc <- (bs_fc*86.0*2 + ms_fc*53.27*2)/2.25   #DALYs averted per year by FICI, doubled so the same as CI or FI
 two-arm trial
s_fcm <- s_fc*scale   # DALYs saved by the combined Facility and Community Intervention if scaled-up to the whole
 of Malawi
fut_efc_=NA*c(1:(length(s_fcm)*length(cycle)))
fut_efc__ <- fut_efc_5 <- fut_efc_4 <- fut_efc_3 <- fut_efc_2 <- fut_efc_1 <- NULL
for (a in 1:length(s_fcm)) {
    for (i in 1:length(cycle)) fut_efc__[i] <-c(s_fcm[a]/((1+edr)^i))
    fut_efc_5[a] <- fut_efc__[i]
}
for (a in 1:length(s_fcm)) {
    for (i in 1:length(cycle)) fut_efc__[i] <-c(s_fcm[a]/((1+edr)^i))
    fut_efc_4[a] <- fut_efc__[i-1]
}
for (a in 1:length(s_fcm)) {
    for (i in 1:length(cycle)) fut_efc__[i] <-c(s_fcm[a]/((1+edr)^i))
    fut_efc_3[a] <- fut_efc__[i-2]
}
for (a in 1:length(s_fcm)) {
    for (i in 1:length(cycle)) fut_efc__[i] <-c(s_fcm[a]/((1+edr)^i))
    fut_efc_2[a] <- fut_efc__[i-3]
}
for (a in 1:length(s_fcm)) {
    for (i in 1:length(cycle)) fut_efc__[i] <-c(s_fcm[a]/((1+edr)^i))
    fut_efc_1[a] <- fut_efc__[i-4]
}
fut_efc_ <- c(fut_efc_1,fut_efc_2,fut_efc_3,fut_efc_4,fut_efc_5)
fut_efc <- tapply( fut_efc_, (seq_along(fut_efc_)-1) %/% years, sum)   # Future effects of FICI for all years in
 time horizon added together, for each of the simulations
start_fc <- 362472*2*scale   # Start-up costs of the combined Facility and Community Intervention for the whole of
 Malawi
annual_fc <- (26289+2047904)*2*scale   # Annual running costs of  the combined Facility and Community Intervention
 for the whole of Malawi
annualext_fc  <- 196042*2*scale   # Annual external costs of FICI for the whole of Malawi
fut_cfc_ <- NULL
for (i in 1:length(cycle)) fut_cfc_[i] <-c((annual_fc + annualext_fc)/((1+cdr)^i))    # Future costs of FI for
 each year in time horizon
fut_cfc <- start_fc + tapply( fut_cfc_, (seq_along(fut_cfc_)-1) %/% years, sum)   # Future costs of FICI for all
 years in time horizon added together, for each of the simulations

        # Future scale-up: Community Intervention (CI) vs Facility Intervention (FI) vs. combined FICI
e4 <- c4 <- matrix(NA,model$BUGSoutput$n.sims,3)
e4 <- matrix(c(fut_ec,fut_ef,fut_efc),model$BUGSoutput$n.sims,3)   # effects - see lines 142, 151 and 161 above
c4 <- matrix(c(rep(fut_cc,model$BUGSoutput$n.sims),rep(fut_cf,model$BUGSoutput$n.sims),rep(fut_cfc,model
 $BUGSoutput$n.sims)),model$BUGSoutput$n.sims,3)   # costs - see lines 147, 156 and 166 above
future <- bcea(e=e4,c=c4,ref=1,interventions=treats3,Kmax=2500)
layout(matrix(1:4,2,2)) #so the four health economic analysis plots from the following commands are on one page
ceplane.plot(future,comparison=2,wtp=779.8)   # Cost-effectiveness plane
eib.plot(future)   # Expected Incremental Benefit
ceac.plot(future)   # Cost-effectiveness Acceptability Curve (prob cost-effective by WTP)
evi.plot(future)  # Expected value of information by willingness to pay
quartz.save("plots_future_5_003_000.pdf", type="pdf")
print("time horizon in years")
years
print("cost discount rate")
cdr
print("effects discount rate")
edr
summary(future,wtp=780)


# .... Future scale-up scenarios 2-9 omitted to save space, the code is identifcal to that for Future scale-up
 scenario 1 above, except for changing the years cdr and edr inputs in the first 3 lines and the
 quartz.save("plots_future_X_XXX_XXX.pdf" command eight lines from the end  ....
```

```
    #Future scale-up 10
years <- 10   # Time Horizon: Years of scale-up projection
cdr <- 0.03   # Cost Discount Rate (per year)
edr <- 0.00  # Effect Discount Rate (per year)
cycle <- c(1:years)
scale <- (15013694*0.04*2)/(1200000*0.04*2)  # scale-up conversion factor - see Table 2 of paper - ratio of trial
 population to total population of Malawi (assume budget for intervention scales proportionally with increased
 population so that the total beneficiaries per year and total cost per year (not discounted) remains the same)

s_cm <- (s_c/2.25)*scale   # DALYs averted per year by the Community Intervention if scaled-up to the whole of
 Malawi
fut_ec_=NA*c(1:(length(s_cm)*length(cycle)))
fut_ec__ <- fut_ec_10 <- fut_ec_9 <- fut_ec_8 <- fut_ec_7 <- fut_ec_6 <-  fut_ec_5 <- fut_ec_4 <- fut_ec_3 <-
 fut_ec_2 <- fut_ec_1 <- NULL
for (a in 1:length(s_cm)) {
    for (i in 1:length(cycle)) fut_ec__[i] <-c(s_cm[a]/((1+edr)^i))
    fut_ec_10[a] <- fut_ec__[i]
}
for (a in 1:length(s_cm)) {
    for (i in 1:length(cycle)) fut_ec__[i] <-c(s_cm[a]/((1+edr)^i))
    fut_ec_9[a] <- fut_ec__[i-1]
}
for (a in 1:length(s_cm)) {
    for (i in 1:length(cycle)) fut_ec__[i] <-c(s_cm[a]/((1+edr)^i))
    fut_ec_8[a] <- fut_ec__[i-2]
}
for (a in 1:length(s_cm)) {
    for (i in 1:length(cycle)) fut_ec__[i] <-c(s_cm[a]/((1+edr)^i))
    fut_ec_7[a] <- fut_ec__[i-3]
}
for (a in 1:length(s_cm)) {
    for (i in 1:length(cycle)) fut_ec__[i] <-c(s_cm[a]/((1+edr)^i))
    fut_ec_6[a] <- fut_ec__[i-4]
}
for (a in 1:length(s_cm)) {
    for (i in 1:length(cycle)) fut_ec__[i] <-c(s_cm[a]/((1+edr)^i))
    fut_ec_5[a] <- fut_ec__[i-5]
}
for (a in 1:length(s_cm)) {
    for (i in 1:length(cycle)) fut_ec__[i] <-c(s_cm[a]/((1+edr)^i))
    fut_ec_4[a] <- fut_ec__[i-6]
}
for (a in 1:length(s_cm)) {
    for (i in 1:length(cycle)) fut_ec__[i] <-c(s_cm[a]/((1+edr)^i))
    fut_ec_3[a] <- fut_ec__[i-7]
}
for (a in 1:length(s_cm)) {
    for (i in 1:length(cycle)) fut_ec__[i] <-c(s_cm[a]/((1+edr)^i))
    fut_ec_2[a] <- fut_ec__[i-8]
}
for (a in 1:length(s_cm)) {
    for (i in 1:length(cycle)) fut_ec__[i] <-c(s_cm[a]/((1+edr)^i))
    fut_ec_1[a] <- fut_ec__[i-9]
}
fut_ec_ <- c(fut_ec_1,fut_ec_2,fut_ec_3,fut_ec_4,fut_ec_5,fut_ec_6,fut_ec_7,fut_ec_8,fut_ec_9,fut_ec_10)
fut_ec <- tapply( fut_ec_, (seq_along(fut_ec_)-1) %/% years, sum)   # Future effects of CI for all years in time
 horizon added together (for each of the simulations)
start_c <- 362083*scale   # Start-up costs of Community Intervention for the whole of Malawi
annual_c <- (27250+2068997)*scale   # Annual running costs of Community Intervention for the whole of Malawi
annualext_c  <- 120067*scale   # Annual external costs of Community Intervention  for the whole of Malawi
fut_cc_ <- NULL
for (i in 1:length(cycle)) fut_cc_[i] <-c((annual_c + annualext_c)/((1+cdr)^i))    # Future costs of CI for each
 year in time horizon
fut_cc <- start_c + tapply( fut_cc_, (seq_along(fut_cc_)-1) %/% years, sum)   # Future costs of CI for all years
 in time horizon added together (this is constant so needs to be replicated by the number of simulations in the
 BCEA)

s_fm <- (s_f/2.25)*scale   # DALYs averted per year by the Facility Intervention if scaled-up to the whole of
 Malawi
fut_ef_=NA*c(1:(length(s_fm)*length(cycle)))
fut_ef__ <- fut_ef_10 <- fut_ef_9 <- fut_ef_8 <- fut_ef_7 <- fut_ef_6 <-  fut_ef_5 <- fut_ef_4 <- fut_ef_3 <-
 fut_ef_2 <- fut_ef_1 <- NULL
for (a in 1:length(s_fm)) {
    for (i in 1:length(cycle)) fut_ef__[i] <-c(s_fm[a]/((1+edr)^i))
    fut_ef_10[a] <- fut_ef__[i]
}
for (a in 1:length(s_fm)) {
```

```
    for (i in 1:length(cycle)) fut_ef__[i] <-c(s_fm[a]/((1+edr)^i))
    fut_ef_9[a] <- fut_ef__[i-1]
}
for (a in 1:length(s_fm)) {
    for (i in 1:length(cycle)) fut_ef__[i] <-c(s_fm[a]/((1+edr)^i))
    fut_ef_8[a] <- fut_ef__[i-2]
}
for (a in 1:length(s_fm)) {
    for (i in 1:length(cycle)) fut_ef__[i] <-c(s_fm[a]/((1+edr)^i))
    fut_ef_7[a] <- fut_ef__[i-3]
}
for (a in 1:length(s_fm)) {
    for (i in 1:length(cycle)) fut_ef__[i] <-c(s_fm[a]/((1+edr)^i))
    fut_ef_6[a] <- fut_ef__[i-4]
}
for (a in 1:length(s_fm)) {
    for (i in 1:length(cycle)) fut_ef__[i] <-c(s_fm[a]/((1+edr)^i))
    fut_ef_5[a] <- fut_ef__[i-5]
}
for (a in 1:length(s_fm)) {
    for (i in 1:length(cycle)) fut_ef__[i] <-c(s_fm[a]/((1+edr)^i))
    fut_ef_4[a] <- fut_ef__[i-6]
}
for (a in 1:length(s_fm)) {
    for (i in 1:length(cycle)) fut_ef__[i] <-c(s_fm[a]/((1+edr)^i))
    fut_ef_3[a] <- fut_ef__[i-7]
}
for (a in 1:length(s_fm)) {
    for (i in 1:length(cycle)) fut_ef__[i] <-c(s_fm[a]/((1+edr)^i))
    fut_ef_2[a] <- fut_ef__[i-8]
}
for (a in 1:length(s_fm)) {
    for (i in 1:length(cycle)) fut_ef__[i] <-c(s_fm[a]/((1+edr)^i))
    fut_ef_1[a] <- fut_ef__[i-9]
}
fut_ef_ <- c(fut_ef_1,fut_ef_2,fut_ef_3,fut_ef_4,fut_ef_5,fut_ef_6,fut_ef_7,fut_ef_8,fut_ef_9,fut_ef_10)
fut_ef <- tapply( fut_ef_, (seq_along(fut_ef_)-1) %/% years, sum)   # Future effects of CI for all years in time
 horizon added together (for each of the simulations)
start_f <- 362860*scale   # Start-up costs of Facility Intervention for the whole of Malawi
annual_f <- (25328+2026811)*scale   # Annual running costs of Facility Intervention for the whole of Malawi
annualext_f  <- 272017*scale   # Annual external costs of Facility Intervention for the whole of Malawi
fut_cf_ <- NULL
for (i in 1:length(cycle)) fut_cf_[i] <-c((annual_f + annualext_f)/((1+cdr)^i))    # Future costs of FI for each
 year in time horizon
fut_cf <- start_f + tapply( fut_cf_, (seq_along(fut_cf_)-1) %/% years, sum)   # Future costs of FI for all years
 in time horizon added together, for each of the simulations

s_fc <- (bs_fc*86.0*2 + ms_fc*53.27*2)/2.25   #DALYs averted per year by FICI, doubled so the same as CI or FI
 two-arm trial
s_fcm <- s_fc*scale   # DALYs saved by the combined Facility and Community Intervention if scaled-up to the whole
 of Malawi
fut_efc_=NA*c(1:(length(s_fcm)*length(cycle)))
fut_efc__ <- fut_efc_10 <- fut_efc_9 <- fut_efc_8 <- fut_efc_7 <- fut_efc_6 <-  fut_efc_5 <- fut_efc_4 <-
 fut_efc_3 <- fut_efc_2 <- fut_efc_1 <- NULL
for (a in 1:length(s_fcm)) {
    for (i in 1:length(cycle)) fut_efc__[i] <-c(s_fcm[a]/((1+edr)^i))
    fut_efc_10[a] <- fut_efc__[i]
}
for (a in 1:length(s_fcm)) {
    for (i in 1:length(cycle)) fut_efc__[i] <-c(s_fcm[a]/((1+edr)^i))
    fut_efc_9[a] <- fut_efc__[i-1]
}
for (a in 1:length(s_fcm)) {
    for (i in 1:length(cycle)) fut_efc__[i] <-c(s_fcm[a]/((1+edr)^i))
    fut_efc_8[a] <- fut_efc__[i-2]
}
for (a in 1:length(s_fcm)) {
    for (i in 1:length(cycle)) fut_efc__[i] <-c(s_fcm[a]/((1+edr)^i))
    fut_efc_7[a] <- fut_efc__[i-3]
}
for (a in 1:length(s_fcm)) {
    for (i in 1:length(cycle)) fut_efc__[i] <-c(s_fcm[a]/((1+edr)^i))
    fut_efc_6[a] <- fut_efc__[i-4]
}
for (a in 1:length(s_fcm)) {
    for (i in 1:length(cycle)) fut_efc__[i] <-c(s_fcm[a]/((1+edr)^i))
    fut_efc_5[a] <- fut_efc__[i-5]
}
for (a in 1:length(s_fcm)) {
```

```
    for (i in 1:length(cycle)) fut_efc__[i] <-c(s_fcm[a]/((1+edr)^i))
    fut_efc_4[a] <- fut_efc__[i-6]
}
for (a in 1:length(s_fcm)) {
    for (i in 1:length(cycle)) fut_efc__[i] <-c(s_fcm[a]/((1+edr)^i))
    fut_efc_3[a] <- fut_efc__[i-7]
}
for (a in 1:length(s_fcm)) {
    for (i in 1:length(cycle)) fut_efc__[i] <-c(s_fcm[a]/((1+edr)^i))
    fut_efc_2[a] <- fut_efc__[i-8]
}
for (a in 1:length(s_fcm)) {
    for (i in 1:length(cycle)) fut_efc__[i] <-c(s_fcm[a]/((1+edr)^i))
    fut_efc_1[a] <- fut_efc__[i-9]
}
fut_efc_ <-
 c(fut_efc_1,fut_efc_2,fut_efc_3,fut_efc_4,fut_efc_5,fut_efc_6,fut_efc_7,fut_efc_8,fut_efc_9,fut_efc_10)
fut_efc <- tapply( fut_efc_, (seq_along(fut_efc_)-1) %/% years, sum)   # Future effects of FICI for all years in
 time horizon added together, for each of the simulations
start_fc <- 362472*2*scale   # Start-up costs of the combined Facility and Community Intervention for the whole of
 Malawi
annual_fc <- (26289+2047904)*2*scale   # Annual running costs of  the combined Facility and Community Intervention
 for the whole of Malawi
annualext_fc  <- 196042*2*scale   # Annual external costs of FICI for the whole of Malawi
fut_cfc_ <- NULL
for (i in 1:length(cycle)) fut_cfc_[i] <-c((annual_fc + annualext_fc)/((1+cdr)^i))    # Future costs of FI for
 each year in time horizon
fut_cfc <- start_fc + tapply( fut_cfc_, (seq_along(fut_cfc_)-1) %/% years, sum)   # Future costs of FICI for all
 years in time horizon added together, for each of the simulations

        # Future scale-up: Community Intervention (CI) vs Facility Intervention (FI) vs. combined FICI
e4 <- c4 <- matrix(NA,model$BUGSoutput$n.sims,3)
e4 <- matrix(c(fut_ec,fut_ef,fut_efc),model$BUGSoutput$n.sims,3)   # effects - see lines 142, 151 and 161 above
c4 <- matrix(c(rep(fut_cc,model$BUGSoutput$n.sims),rep(fut_cf,model$BUGSoutput$n.sims),rep(fut_cfc,model
 $BUGSoutput$n.sims)),model$BUGSoutput$n.sims,3)   # costs - see lines 147, 156 and 166 above
future <- bcea(e=e4,c=c4,ref=1,interventions=treats3,Kmax=2500)
layout(matrix(1:4,2,2)) #so the four health economic analysis plots from the following commands are on one page
ceplane.plot(future,comparison=2,wtp=779.8)   # Cost-effectiveness plane
eib.plot(future)    # Expected Incremental Benefit
ceac.plot(future)   # Cost-effectiveness Acceptability Curve (prob cost-effective by WTP)
evi.plot(future)   # Expected value of information by willingness to pay
quartz.save("plots_future_10_003_000.pdf", type="pdf")
print("time horizon in years")
years
print("cost discount rate")
cdr
print("effects discount rate")
edr
summary(future,wtp=780)


# .... Future scale-up scenarios 11-18 omitted to save space, the code is identifcal to that for Future scale-up
 scenario 10 above, except for changing the years cdr and edr inputs in the first 3 lines and the
 quartz.save("plots_future_X_XXX_XXX.pdf" command eight lines from the end  ....

    #Future scale-up 19
years <- 20   # Time Horizon: Years of scale-up projection
cdr <- 0.03   # Cost Discount Rate (per year)
edr <- 0.00  # Effect Discount Rate (per year)
cycle <- c(1:years)
scale <- (15013694*0.04*2)/(1200000*0.04*2)  # scale-up conversion factor - see Table 2 of paper - ratio of trial
 population to total population of Malawi (assume budget for intervention scales proportionally with increased
 population so that the total beneficiaries per year and total cost per year (not discounted) remains the same)

s_cm <- (s_c/2.25)*scale   # DALYs averted per year by the Community Intervention if scaled-up to the whole of
 Malawi
fut_ec_=NA*c(1:(length(s_cm)*length(cycle)))
fut_ec__ <- fut_ec_20 <- fut_ec_19 <- fut_ec_18 <- fut_ec_17 <- fut_ec_16 <- fut_ec_15 <- fut_ec_14 <- fut_ec_13
 <- fut_ec_12 <- fut_ec_11 <- fut_ec_10 <- fut_ec_9 <- fut_ec_8 <- fut_ec_7 <- fut_ec_6 <-  fut_ec_5 <- fut_ec_4 <-
 fut_ec_3 <- fut_ec_2 <- fut_ec_1 <- NULL
for (a in 1:length(s_cm)) {
    for (i in 1:length(cycle)) fut_ec__[i] <-c(s_cm[a]/((1+edr)^i))
    fut_ec_20[a] <- fut_ec__[i]
}
for (a in 1:length(s_cm)) {
    for (i in 1:length(cycle)) fut_ec__[i] <-c(s_cm[a]/((1+edr)^i))
    fut_ec_19[a] <- fut_ec__[i-1]
}
for (a in 1:length(s_cm)) {
```

```
    for (i in 1:length(cycle)) fut_ec__[i] <-c(s_cm[a]/((1+edr)^i))
    fut_ec_18[a] <- fut_ec__[i-2]
}
for (a in 1:length(s_cm)) {
    for (i in 1:length(cycle)) fut_ec__[i] <-c(s_cm[a]/((1+edr)^i))
    fut_ec_17[a] <- fut_ec__[i-3]
}
for (a in 1:length(s_cm)) {
    for (i in 1:length(cycle)) fut_ec__[i] <-c(s_cm[a]/((1+edr)^i))
    fut_ec_16[a] <- fut_ec__[i-4]
}
for (a in 1:length(s_cm)) {
    for (i in 1:length(cycle)) fut_ec__[i] <-c(s_cm[a]/((1+edr)^i))
    fut_ec_15[a] <- fut_ec__[i-5]
}
for (a in 1:length(s_cm)) {
    for (i in 1:length(cycle)) fut_ec__[i] <-c(s_cm[a]/((1+edr)^i))
    fut_ec_14[a] <- fut_ec__[i-6]
}
for (a in 1:length(s_cm)) {
    for (i in 1:length(cycle)) fut_ec__[i] <-c(s_cm[a]/((1+edr)^i))
    fut_ec_13[a] <- fut_ec__[i-7]
}
for (a in 1:length(s_cm)) {
    for (i in 1:length(cycle)) fut_ec__[i] <-c(s_cm[a]/((1+edr)^i))
    fut_ec_12[a] <- fut_ec__[i-8]
}
for (a in 1:length(s_cm)) {
    for (i in 1:length(cycle)) fut_ec__[i] <-c(s_cm[a]/((1+edr)^i))
    fut_ec_11[a] <- fut_ec__[i-9]
}
for (a in 1:length(s_cm)) {
    for (i in 1:length(cycle)) fut_ec__[i] <-c(s_cm[a]/((1+edr)^i))
    fut_ec_10[a] <- fut_ec__[i-10]
}
for (a in 1:length(s_cm)) {
    for (i in 1:length(cycle)) fut_ec__[i] <-c(s_cm[a]/((1+edr)^i))
    fut_ec_9[a] <- fut_ec__[i-11]
}
for (a in 1:length(s_cm)) {
    for (i in 1:length(cycle)) fut_ec__[i] <-c(s_cm[a]/((1+edr)^i))
    fut_ec_8[a] <- fut_ec__[i-12]
}
for (a in 1:length(s_cm)) {
    for (i in 1:length(cycle)) fut_ec__[i] <-c(s_cm[a]/((1+edr)^i))
    fut_ec_7[a] <- fut_ec__[i-13]
}
for (a in 1:length(s_cm)) {
    for (i in 1:length(cycle)) fut_ec__[i] <-c(s_cm[a]/((1+edr)^i))
    fut_ec_6[a] <- fut_ec__[i-14]
}
for (a in 1:length(s_cm)) {
    for (i in 1:length(cycle)) fut_ec__[i] <-c(s_cm[a]/((1+edr)^i))
    fut_ec_5[a] <- fut_ec__[i-15]
}
for (a in 1:length(s_cm)) {
    for (i in 1:length(cycle)) fut_ec__[i] <-c(s_cm[a]/((1+edr)^i))
    fut_ec_4[a] <- fut_ec__[i-16]
}
for (a in 1:length(s_cm)) {
    for (i in 1:length(cycle)) fut_ec__[i] <-c(s_cm[a]/((1+edr)^i))
    fut_ec_3[a] <- fut_ec__[i-17]
}
for (a in 1:length(s_cm)) {
    for (i in 1:length(cycle)) fut_ec__[i] <-c(s_cm[a]/((1+edr)^i))
    fut_ec_2[a] <- fut_ec__[i-18]
}
for (a in 1:length(s_cm)) {
    for (i in 1:length(cycle)) fut_ec__[i] <-c(s_cm[a]/((1+edr)^i))
    fut_ec_1[a] <- fut_ec__[i-19]
}
fut_ec_ <-
 c(fut_ec_1,fut_ec_2,fut_ec_3,fut_ec_4,fut_ec_5,fut_ec_6,fut_ec_7,fut_ec_8,fut_ec_9,fut_ec_10,fut_ec_11,fut_ec_12,f
 ut_ec_13,fut_ec_14,fut_ec_15,fut_ec_16,fut_ec_17,fut_ec_18,fut_ec_19,fut_ec_20)
fut_ec <- tapply( fut_ec_, (seq_along(fut_ec_)-1) %/% years, sum)   # Future effects of CI for all years in time
 horizon added together (for each of the simulations)
start_c <- 362083*scale   # Start-up costs of Community Intervention for the whole of Malawi
annual_c <- (27250+2068997)*scale   # Annual running costs of Community Intervention for the whole of Malawi
annualext_c  <- 120067*scale   # Annual external costs of Community Intervention  for the whole of Malawi
```

```
fut_cc_ <- NULL
for (i in 1:length(cycle)) fut_cc_[i] <-c((annual_c + annualext_c)/((1+cdr)^i))    # Future costs of CI for each
 year in time horizon
fut_cc <- start_c + tapply( fut_cc_, (seq_along(fut_cc_)-1) %/% years, sum)   # Future costs of CI for all years
 in time horizon added together (this is constant so needs to be replicated by the number of simulations in the
 BCEA)

s_fm <- (s_f/2.25)*scale   # DALYs averted per year by the Facility Intervention if scaled-up to the whole of
 Malawi
fut_ef_=NA*c(1:(length(s_fm)*length(cycle)))
fut_ef__ <- fut_ef_20 <- fut_ef_19 <- fut_ef_18 <- fut_ef_17 <- fut_ef_16 <- fut_ef_15 <- fut_ef_14 <- fut_ef_13
 <- fut_ef_12 <- fut_ef_11 <- fut_ef_10 <- fut_ef_9 <- fut_ef_8 <- fut_ef_7 <- fut_ef_6 <-  fut_ef_5 <- fut_ef_4 <-
 fut_ef_3 <- fut_ef_2 <- fut_ef_1 <- NULL
for (a in 1:length(s_fm)) {
    for (i in 1:length(cycle)) fut_ef__[i] <-c(s_fm[a]/((1+edr)^i))
    fut_ef_20[a] <- fut_ef__[i]
}
for (a in 1:length(s_fm)) {
    for (i in 1:length(cycle)) fut_ef__[i] <-c(s_fm[a]/((1+edr)^i))
    fut_ef_19[a] <- fut_ef__[i-1]
}
for (a in 1:length(s_fm)) {
    for (i in 1:length(cycle)) fut_ef__[i] <-c(s_fm[a]/((1+edr)^i))
    fut_ef_18[a] <- fut_ef__[i-2]
}
for (a in 1:length(s_fm)) {
    for (i in 1:length(cycle)) fut_ef__[i] <-c(s_fm[a]/((1+edr)^i))
    fut_ef_17[a] <- fut_ef__[i-3]
}
for (a in 1:length(s_fm)) {
    for (i in 1:length(cycle)) fut_ef__[i] <-c(s_fm[a]/((1+edr)^i))
    fut_ef_16[a] <- fut_ef__[i-4]
}
for (a in 1:length(s_fm)) {
    for (i in 1:length(cycle)) fut_ef__[i] <-c(s_fm[a]/((1+edr)^i))
    fut_ef_15[a] <- fut_ef__[i-5]
}
for (a in 1:length(s_fm)) {
    for (i in 1:length(cycle)) fut_ef__[i] <-c(s_fm[a]/((1+edr)^i))
    fut_ef_14[a] <- fut_ef__[i-6]
}
for (a in 1:length(s_fm)) {
    for (i in 1:length(cycle)) fut_ef__[i] <-c(s_fm[a]/((1+edr)^i))
    fut_ef_13[a] <- fut_ef__[i-7]
}
for (a in 1:length(s_fm)) {
    for (i in 1:length(cycle)) fut_ef__[i] <-c(s_fm[a]/((1+edr)^i))
    fut_ef_12[a] <- fut_ef__[i-8]
}
for (a in 1:length(s_fm)) {
    for (i in 1:length(cycle)) fut_ef__[i] <-c(s_fm[a]/((1+edr)^i))
    fut_ef_11[a] <- fut_ef__[i-9]
}
for (a in 1:length(s_fm)) {
    for (i in 1:length(cycle)) fut_ef__[i] <-c(s_fm[a]/((1+edr)^i))
    fut_ef_10[a] <- fut_ef__[i-10]
}
for (a in 1:length(s_fm)) {
    for (i in 1:length(cycle)) fut_ef__[i] <-c(s_fm[a]/((1+edr)^i))
    fut_ef_9[a] <- fut_ef__[i-11]
}
for (a in 1:length(s_fm)) {
    for (i in 1:length(cycle)) fut_ef__[i] <-c(s_fm[a]/((1+edr)^i))
    fut_ef_8[a] <- fut_ef__[i-12]
}
for (a in 1:length(s_fm)) {
    for (i in 1:length(cycle)) fut_ef__[i] <-c(s_fm[a]/((1+edr)^i))
    fut_ef_7[a] <- fut_ef__[i-13]
}
for (a in 1:length(s_fm)) {
    for (i in 1:length(cycle)) fut_ef__[i] <-c(s_fm[a]/((1+edr)^i))
    fut_ef_6[a] <- fut_ef__[i-14]
}
for (a in 1:length(s_fm)) {
    for (i in 1:length(cycle)) fut_ef__[i] <-c(s_fm[a]/((1+edr)^i))
    fut_ef_5[a] <- fut_ef__[i-15]
}
for (a in 1:length(s_fm)) {
    for (i in 1:length(cycle)) fut_ef__[i] <-c(s_fm[a]/((1+edr)^i))
```

```
    fut_ef_4[a] <- fut_ef__[i-16]
}
for (a in 1:length(s_fm)) {
    for (i in 1:length(cycle)) fut_ef__[i] <-c(s_fm[a]/((1+edr)^i))
    fut_ef_3[a] <- fut_ef__[i-17]
}
for (a in 1:length(s_fm)) {
    for (i in 1:length(cycle)) fut_ef__[i] <-c(s_fm[a]/((1+edr)^i))
    fut_ef_2[a] <- fut_ef__[i-18]
}
for (a in 1:length(s_fm)) {
    for (i in 1:length(cycle)) fut_ef__[i] <-c(s_fm[a]/((1+edr)^i))
    fut_ef_1[a] <- fut_ef__[i-19]
}
fut_ef_ <-
 c(fut_ef_1,fut_ef_2,fut_ef_3,fut_ef_4,fut_ef_5,fut_ef_6,fut_ef_7,fut_ef_8,fut_ef_9,fut_ef_10,fut_ef_11,fut_ef_12,f
 ut_ef_13,fut_ef_14,fut_ef_15,fut_ef_16,fut_ef_17,fut_ef_18,fut_ef_19,fut_ef_20)
fut_ef <- tapply( fut_ef_, (seq_along(fut_ef_)-1) %/% years, sum)   # Future effects of CI for all years in time
 horizon added together (for each of the simulations)
start_f <- 362860*scale   # Start-up costs of Facility Intervention for the whole of Malawi
annual_f <- (25328+2026811)*scale   # Annual running costs of Facility Intervention for the whole of Malawi
annualext_f  <- 272017*scale   # Annual external costs of Facility Intervention for the whole of Malawi
fut_cf_ <- NULL
for (i in 1:length(cycle)) fut_cf_[i] <-c((annual_f + annualext_f)/((1+cdr)^i))    # Future costs of FI for each
 year in time horizon
fut_cf <- start_f + tapply( fut_cf_, (seq_along(fut_cf_)-1) %/% years, sum)   # Future costs of FI for all years
 in time horizon added together, for each of the simulations

s_fc <- (bs_fc*86.0*2 + ms_fc*53.27*2)/2.25   #DALYs averted per year by FICI, doubled so the same as CI or FI
 two-arm trial
s_fcm <- s_fc*scale   # DALYs saved by the combined Facility and Community Intervention if scaled-up to the whole
 of Malawi
fut_efc_=NA*c(1:(length(s_fcm)*length(cycle)))
fut_efc__ <- fut_efc_20 <- fut_efc_19 <- fut_efc_18 <- fut_efc_17 <- fut_efc_16 <- fut_efc_15 <- fut_efc_14 <-
 fut_efc_13 <- fut_efc_12 <- fut_efc_11 <- fut_efc_10 <- fut_efc_9 <- fut_efc_8 <- fut_efc_7 <- fut_efc_6 <-
 fut_efc_5 <- fut_efc_4 <- fut_efc_3 <- fut_efc_2 <- fut_efc_1 <- NULL
for (a in 1:length(s_fcm)) {
    for (i in 1:length(cycle)) fut_efc__[i] <-c(s_fcm[a]/((1+edr)^i))
    fut_efc_20[a] <- fut_efc__[i]
}
for (a in 1:length(s_fcm)) {
    for (i in 1:length(cycle)) fut_efc__[i] <-c(s_fcm[a]/((1+edr)^i))
    fut_efc_19[a] <- fut_efc__[i-1]
}
for (a in 1:length(s_fcm)) {
    for (i in 1:length(cycle)) fut_efc__[i] <-c(s_fcm[a]/((1+edr)^i))
    fut_efc_18[a] <- fut_efc__[i-2]
}
for (a in 1:length(s_fcm)) {
    for (i in 1:length(cycle)) fut_efc__[i] <-c(s_fcm[a]/((1+edr)^i))
    fut_efc_17[a] <- fut_efc__[i-3]
}
for (a in 1:length(s_fcm)) {
    for (i in 1:length(cycle)) fut_efc__[i] <-c(s_fcm[a]/((1+edr)^i))
    fut_efc_16[a] <- fut_efc__[i-4]
}
for (a in 1:length(s_fcm)) {
    for (i in 1:length(cycle)) fut_efc__[i] <-c(s_fcm[a]/((1+edr)^i))
    fut_efc_15[a] <- fut_efc__[i-5]
}
for (a in 1:length(s_fcm)) {
    for (i in 1:length(cycle)) fut_efc__[i] <-c(s_fcm[a]/((1+edr)^i))
    fut_efc_14[a] <- fut_efc__[i-6]
}
for (a in 1:length(s_fcm)) {
    for (i in 1:length(cycle)) fut_efc__[i] <-c(s_fcm[a]/((1+edr)^i))
    fut_efc_13[a] <- fut_efc__[i-7]
}
for (a in 1:length(s_fcm)) {
    for (i in 1:length(cycle)) fut_efc__[i] <-c(s_fcm[a]/((1+edr)^i))
    fut_efc_12[a] <- fut_efc__[i-8]
}
for (a in 1:length(s_fcm)) {
    for (i in 1:length(cycle)) fut_efc__[i] <-c(s_fcm[a]/((1+edr)^i))
    fut_efc_11[a] <- fut_efc__[i-9]
}
for (a in 1:length(s_fcm)) {
    for (i in 1:length(cycle)) fut_efc__[i] <-c(s_fcm[a]/((1+edr)^i))
    fut_efc_10[a] <- fut_efc__[i-10]
```

```
}
for (a in 1:length(s_fcm)) {
    for (i in 1:length(cycle)) fut_efc__[i] <-c(s_fcm[a]/((1+edr)^i))
    fut_efc_9[a] <- fut_efc__[i-11]
}
for (a in 1:length(s_fcm)) {
    for (i in 1:length(cycle)) fut_efc__[i] <-c(s_fcm[a]/((1+edr)^i))
    fut_efc_8[a] <- fut_efc__[i-12]
}
for (a in 1:length(s_fcm)) {
    for (i in 1:length(cycle)) fut_efc__[i] <-c(s_fcm[a]/((1+edr)^i))
    fut_efc_7[a] <- fut_efc__[i-13]
}
for (a in 1:length(s_fcm)) {
    for (i in 1:length(cycle)) fut_efc__[i] <-c(s_fcm[a]/((1+edr)^i))
    fut_efc_6[a] <- fut_efc__[i-14]
}
for (a in 1:length(s_fcm)) {
    for (i in 1:length(cycle)) fut_efc__[i] <-c(s_fcm[a]/((1+edr)^i))
    fut_efc_5[a] <- fut_efc__[i-15]
}
for (a in 1:length(s_fcm)) {
    for (i in 1:length(cycle)) fut_efc__[i] <-c(s_fcm[a]/((1+edr)^i))
    fut_efc_4[a] <- fut_efc__[i-16]
}
for (a in 1:length(s_fcm)) {
    for (i in 1:length(cycle)) fut_efc__[i] <-c(s_fcm[a]/((1+edr)^i))
    fut_efc_3[a] <- fut_efc__[i-17]
}
for (a in 1:length(s_fcm)) {
    for (i in 1:length(cycle)) fut_efc__[i] <-c(s_fcm[a]/((1+edr)^i))
    fut_efc_2[a] <- fut_efc__[i-18]
}
for (a in 1:length(s_fcm)) {
    for (i in 1:length(cycle)) fut_efc__[i] <-c(s_fcm[a]/((1+edr)^i))
    fut_efc_1[a] <- fut_efc__[i-19]
}
fut_efc_ <-
 c(fut_efc_1,fut_efc_2,fut_efc_3,fut_efc_4,fut_efc_5,fut_efc_6,fut_efc_7,fut_efc_8,fut_efc_9,fut_efc_10,fut_efc_11,
 fut_efc_12,fut_efc_13,fut_efc_14,fut_efc_15,fut_efc_16,fut_efc_17,fut_efc_18,fut_efc_19,fut_efc_20)
fut_efc <- tapply( fut_efc_, (seq_along(fut_efc_)-1) %/% years, sum)   # Future effects of FICI for all years in
 time horizon added together, for each of the simulations
start_fc <- 362472*2*scale   # Start-up costs of the combined Facility and Community Intervention for the whole of
 Malawi
annual_fc <- (26289+2047904)*2*scale   # Annual running costs of  the combined Facility and Community Intervention
 for the whole of Malawi
annualext_fc  <- 196042*2*scale   # Annual external costs of FICI for the whole of Malawi
fut_cfc_ <- NULL
for (i in 1:length(cycle)) fut_cfc_[i] <-c((annual_fc + annualext_fc)/((1+cdr)^i))    # Future costs of FI for
 each year in time horizon
fut_cfc <- start_fc + tapply( fut_cfc_, (seq_along(fut_cfc_)-1) %/% years, sum)   # Future costs of FICI for all
 years in time horizon added together, for each of the simulations

        # Future scale-up: Community Intervention (CI) vs Facility Intervention (FI) vs. combined FICI
e4 <- c4 <- matrix(NA,model$BUGSoutput$n.sims,3)
e4 <- matrix(c(fut_ec,fut_ef,fut_efc),model$BUGSoutput$n.sims,3)   # effects - see lines 142, 151 and 161 above
c4 <- matrix(c(rep(fut_cc,model$BUGSoutput$n.sims),rep(fut_cf,model$BUGSoutput$n.sims),rep(fut_cfc,model
 $BUGSoutput$n.sims)),model$BUGSoutput$n.sims,3)   # costs - see lines 147, 156 and 166 above
future <- bcea(e=e4,c=c4,ref=1,interventions=treats3,Kmax=2500)
layout(matrix(1:4,2,2)) #so the four health economic analysis plots from the following commands are on one page
ceplane.plot(future,comparison=2,wtp=779.8)   # Cost-effectiveness plane
eib.plot(future)    # Expected Incremental Benefit
ceac.plot(future)   # Cost-effectiveness Acceptability Curve (prob cost-effective by WTP)
evi.plot(future)   # Expected value of information by willingness to pay
quartz.save("plots_future_20_003_000.pdf", type="pdf")
print("time horizon in years")
years
print("cost discount rate")
cdr
print("effects discount rate")
edr
summary(future,wtp=780)

# .... Future scale-up scenarios 20-27 omitted to save space, the code is identifcal to that for Future scale-up
 scenario 19 above, except for changing the years cdr and edr inputs in the first 3 lines and the
 quartz.save("plots_future_X_XXX_XXX.pdf" command eight lines from the end

sink()
```

```
# MaiKhanda_final_log_.txt output from MaiKhanda_final_submit.R   3rd Dec 10:12

The downloaded binary packages are in
        /var/folders/ps/63xh8zd90wbdcl2v8l10_npc0000gq/T//RtmpavJqyt/downloaded_packages

The downloaded binary packages are in
        /var/folders/ps/63xh8zd90wbdcl2v8l10_npc0000gq/T//RtmpavJqyt/downloaded_packages
Compiling model graph
   Resolving undeclared variables
   Allocating nodes
   Graph Size: 702

Initializing model

Inference for Bugs model at "/Users/timothycolbourn/Documents/Papers/MaiKhanda CEA paper/JAGS
revised/MKmodel_final.txt", fit using jags,
 2 chains, each with 510000 iterations (first 10000 discarded), n.thin = 10
 n.sims = 100000 iterations saved
            mu.vect   sd.vect       2.5%        50%      97.5%  Rhat  n.eff
bs_c     102352.276   336.338 101673.738 102361.079 102978.330 1.001 100000
bs_f     101621.174   368.950 100873.777 101630.223 102316.649 1.001  33000
bs_nc    101580.075    77.652 101427.000 101580.000 101731.000 1.001  76000
bs_nf    101329.914    79.146 101175.000 101330.000 101485.000 1.001 100000
d_c       67360.641 29006.136   8807.651  68124.694 121507.556 1.001 100000
d_f       19900.924 32103.889 -44768.651  20638.361  80586.481 1.001  21000
db_c      66409.268 28324.616   9415.513  67146.479 119034.719 1.001 100000
db_f      25048.424 31049.592 -37739.441  25770.955  83762.741 1.001  34000
dm_c        951.373  6523.040 -14369.912   1835.218  11169.105 1.001  57000
dm_f      -5147.499  8559.492 -25540.628  -3935.924   7883.364 1.004    480
lb_c        772.201   329.356    109.483    780.773   1384.125 1.001 100000
lb_f        291.261   361.042   -438.831    299.662    973.985 1.001  34000
lm_c         17.859   122.452   -269.756     34.451    209.670 1.001  57000
lm_f        -96.630   160.681   -479.456    -73.886    147.989 1.004    480
ms_c     107605.037   123.931 107313.273 107622.046 107797.303 1.001  64000
ms_f     107477.274   162.687 107089.672 107500.481 107724.933 1.004    500
ms_nc    107587.178    20.335 107547.000 107587.000 107626.000 1.001 100000
ms_nf    107573.905    20.467 107533.000 107574.000 107614.000 1.001  37000
orb_c         0.873     0.054      0.774      0.872      0.982 1.001 100000
orb_f         0.954     0.057      0.846      0.952      1.070 1.001  33000
orm_c         0.957     0.298      0.494      0.916      1.657 1.001  30000
orm_f         1.228     0.379      0.652      1.175      2.135 1.004    430
deviance    509.790   274.274    500.653    507.739    519.946 1.019   6700

For each parameter, n.eff is a crude measure of effective sample size,
and Rhat is the potential scale reduction factor (at convergence, Rhat=1).

DIC info (using the rule, pD = var(deviance)/2)
pD = 37613.2 and DIC = 38123.0
DIC is an estimate of expected predictive error (lower deviance is better).
[[1]]

Quantile (q) = 0.025
Accuracy (r) = +/- 0.005
Probability (s) = 0.95

          Burn-in  Total   Lower bound  Dependence
          (M)      (N)     (Nmin)       factor (I)
 bs_c     60       90720   3746          24.2
 bs_f     60       86860   3746          23.2
 bs_nc    20       38160   3746          10.2
 bs_nf    20       37480   3746          10.0
 d_c      60       90920   3746          24.3
 d_f      80       94800   3746          25.3
 db_c     60       87780   3746          23.4
 db_f     80       91840   3746          24.5
 deviance 180      240360  3746          64.2
 dm_c     910      1058070 3746         282.0
 dm_f     1080     1107540 3746         296.0
 lb_c     60       87780   3746          23.4
 lb_f     80       91840   3746          24.5
 lm_c     910      1058070 3746         282.0
 lm_f     1080     1107540 3746         296.0
 ms_c     840      1026480 3746         274.0
 ms_f     990      972090  3746         260.0
 ms_nc    20       41880   3746          11.2
 ms_nf    10       37530   3746          10.0
 orb_c    60       90160   3746          24.1
 orb_f    80       91960   3746          24.5
 orm_c    1430     1476530 3746         394.0
 orm_f    900      944800  3746         252.0


[[2]]
```

Quantile (q) = 0.025
Accuracy (r) = +/- 0.005
Probability (s) = 0.95

	Burn-in (M)	Total (N)	Lower bound (Nmin)	Dependence factor (I)
bs_c	60	86700	3746	23.1
bs_f	60	89360	3746	23.9
bs_nc	10	38720	3746	10.3
bs_nf	20	38510	3746	10.3
d_c	80	98980	3746	26.4
d_f	60	91900	3746	24.5
db_c	60	90760	3746	24.2
db_f	60	96760	3746	25.8
deviance	210	289240	3746	77.2
dm_c	1120	1281840	3746	342.0
dm_f	840	943460	3746	252.0
lb_c	60	90760	3746	24.2
lb_f	60	96760	3746	25.8
lm_c	1120	1281840	3746	342.0
lm_f	840	943460	3746	252.0
ms_c	800	878400	3746	234.0
ms_f	910	987350	3746	264.0
ms_nc	20	39660	3746	10.6
ms_nf	20	38060	3746	10.2
orb_c	60	91020	3746	24.3
orb_f	90	129420	3746	34.5
orm_c	1320	1526400	3746	407.0
orm_f	1080	1112940	3746	297.0

Iterations = 10001:509991
Thinning interval = 10
Number of chains = 2
Sample size per chain = 50000

1. Empirical mean and standard deviation for each variable,
   plus standard error of the mean:

	Mean	SD	Naive SE	Time-series SE
bs_c	1.024e+05	3.363e+02	1.064e+00	2.098e+00
bs_f	1.016e+05	3.690e+02	1.167e+00	2.319e+00
bs_nc	1.016e+05	7.765e+01	2.456e-01	2.456e-01
bs_nf	1.013e+05	7.915e+01	2.503e-01	2.513e-01
d_c	6.736e+04	2.901e+04	9.173e+01	2.288e+02
d_f	1.990e+04	3.210e+04	1.015e+02	3.015e+02
db_c	6.641e+04	2.832e+04	8.957e+01	1.807e+02
db_f	2.505e+04	3.105e+04	9.819e+01	1.970e+02
deviance	5.098e+02	2.743e+02	8.673e-01	1.030e+00
dm_c	9.514e+02	6.523e+03	2.063e+01	2.049e+02
dm_f	-5.147e+03	8.559e+03	2.707e+01	2.643e+02
lb_c	7.722e+02	3.294e+02	1.042e+00	2.101e+00
lb_f	2.913e+02	3.610e+02	1.142e+00	2.291e+00
lm_c	1.786e+01	1.225e+02	3.872e-01	3.847e+00
lm_f	-9.663e+01	1.607e+02	5.081e-01	4.962e+00
ms_c	1.076e+05	1.239e+02	3.919e-01	3.884e+00
ms_f	1.075e+05	1.627e+02	5.145e-01	4.995e+00
ms_nc	1.076e+05	2.033e+01	6.430e-02	6.430e-02
ms_nf	1.076e+05	2.047e+01	6.472e-02	6.472e-02
orb_c	8.733e-01	5.390e-02	1.704e-04	3.430e-04
orb_f	9.538e-01	5.740e-02	1.815e-04	3.640e-04
orm_c	9.570e-01	2.976e-01	9.410e-04	9.251e-03
orm_f	1.228e+00	3.790e-01	1.199e-03	1.176e-02

2. Quantiles for each variable:

	2.5%	25%	50%	75%	97.5%
bs_c	1.017e+05	1.021e+05	1.024e+05	1.026e+05	1.030e+05
bs_f	1.009e+05	1.014e+05	1.016e+05	1.019e+05	1.023e+05
bs_nc	1.014e+05	1.015e+05	1.016e+05	1.016e+05	1.017e+05
bs_nf	1.012e+05	1.013e+05	1.013e+05	1.014e+05	1.015e+05
d_c	8.808e+03	4.842e+04	6.812e+04	8.727e+04	1.215e+05
d_f	-4.477e+04	-1.213e+03	2.064e+04	4.175e+04	8.059e+04
db_c	9.416e+03	4.786e+04	6.715e+04	8.586e+04	1.190e+05
db_f	-3.774e+04	4.530e+03	2.577e+04	4.624e+04	8.376e+04
deviance	5.007e+02	5.048e+02	5.077e+02	5.113e+02	5.199e+02
dm_c	-1.437e+04	-2.689e+03	1.835e+03	5.568e+03	1.117e+04
dm_f	-2.554e+04	-9.837e+03	-3.936e+03	9.889e+02	7.883e+03
lb_c	1.095e+02	5.565e+02	7.808e+02	9.984e+02	1.384e+03
lb_f	-4.388e+02	5.268e+01	2.997e+02	5.376e+02	9.740e+02
lm_c	-2.698e+02	-5.049e+01	3.445e+01	1.045e+02	2.097e+02
lm_f	-4.795e+02	-1.847e+02	-7.389e+01	1.856e+01	1.480e+02
ms_c	1.073e+05	1.075e+05	1.076e+05	1.077e+05	1.078e+05

```
ms_f      1.071e+05  1.074e+05  1.075e+05 1.076e+05 1.077e+05
ms_nc     1.075e+05  1.076e+05  1.076e+05 1.076e+05 1.076e+05
ms_nf     1.075e+05  1.076e+05  1.076e+05 1.076e+05 1.076e+05
orb_c     7.740e-01  8.363e-01  8.717e-01 9.083e-01 9.819e-01
orb_f     8.459e-01  9.145e-01  9.523e-01 9.916e-01 1.070e+00
orm_c     4.943e-01  7.454e-01  9.160e-01 1.123e+00 1.657e+00
orm_f     6.517e-01  9.563e-01  1.175e+00 1.436e+00 2.135e+00

quartz
     2

Cost-effectiveness analysis summary

Reference intervention: Community Intervention
Comparator intervention: do nothing

Optimal decision: choose do nothing for k<80 and Community Intervention for k>=80


Analysis for willingness to pay parameter k = 780

                       Expected utility
do nothing                  11284303221
Community Intervention      11331495730

                                          EIB    CEAC   ICER
Community Intervention vs do nothing 47192509 0.97832 79.405

Optimal intervention (max expected utility) for k=780: Community Intervention

EVPI 210423
quartz
     2

Cost-effectiveness analysis summary

Reference intervention: Facility Intervention
Comparator intervention: do nothing

Optimal decision: choose do nothing for k<285 and Facility Intervention for k>=285


Analysis for willingness to pay parameter k = 780

                      Expected utility
do nothing                 11266970900
Facility Intervention      11276901409

                                        EIB    CEAC ICER
Facility Intervention vs do nothing 9930509 0.66163  281

Optimal intervention (max expected utility) for k=780: Facility Intervention

EVPI 5851010
quartz
     2

Cost-effectiveness analysis summary

Reference intervention: Community Intervention
Comparator intervention: Facility Intervention

Community Intervention dominates for all k in [0 - 2500]


Analysis for willingness to pay parameter k = 780

                       Expected utility
Community Intervention      11331495730
Facility Intervention       11276901409

                                                     EIB    CEAC    ICER
Community Intervention vs Facility Intervention 54594321 0.94599 -3.4934

Optimal intervention (max expected utility) for k=780: Community Intervention

EVPI 793159
Compiling model graph
   Resolving undeclared variables
   Allocating nodes
   Graph Size: 357

Initializing model
```

```
Inference for Bugs model at "/Users/timothycolbourn/Documents/Papers/MaiKhanda CEA paper/JAGS
revised/MKmodelFC_final.txt", fit using jags,
 2 chains, each with 510000 iterations (first 10000 discarded), n.thin = 10
 n.sims = 100000 iterations saved
           mu.vect   sd.vect       2.5%       50%      97.5%  Rhat  n.eff
bs_con   50862.940    54.468  50756.000 50863.000  50969.000 1.001  46000
bs_fc    51338.360   223.893  50873.579 51346.994  51750.893 1.001  21000
d_fc     37590.203 20198.564  -4641.764 38498.852  74617.980 1.001   3100
d_fc_    75180.406 40397.128  -9283.528 76997.703 149235.960 1.001   3100
db_fc    40886.076 18860.691   1982.103 41544.463  75674.721 1.001  29000
db_fc_   81772.153 37721.381   3964.205 83088.927 151349.441 1.001  29000
dm_fc    -3295.873  7298.995 -21134.687 -1728.280   5950.436 1.003    830
dm_fc_   -6591.747 14597.989 -42269.374 -3456.560  11900.872 1.003    830
lb_fc      475.419   219.310     23.048   483.075    879.939 1.001  29000
lb_fc_     950.839   438.621     46.095   966.150   1759.877 1.001  29000
lm_fc      -61.871   137.019   -396.747   -32.444    111.703 1.003    830
lm_fc_    -123.742   274.038   -793.493   -64.888    223.407 1.003    830
ms_con   53782.118    14.783  53753.000 53782.000  53811.000 1.001 100000
ms_fc    53720.247   138.299  53383.775 53749.854  53894.724 1.003    840
orb_fc       0.841     0.073      0.707     0.838      0.992 1.001  28000
orm_fc       1.287     0.635      0.488     1.150      2.839 1.003    600
deviance   258.437    64.142    251.107   257.322    269.176 1.001   8100

For each parameter, n.eff is a crude measure of effective sample size,
and Rhat is the potential scale reduction factor (at convergence, Rhat=1).

DIC info (using the rule, pD = var(deviance)/2)
pD = 2057.1 and DIC = 2315.5
DIC is an estimate of expected predictive error (lower deviance is better).
[[1]]

Quantile (q) = 0.025
Accuracy (r) = +/- 0.005
Probability (s) = 0.95

          Burn-in  Total   Lower bound  Dependence
          (M)      (N)     (Nmin)       factor (I)
 bs_con   20       39230   3746          10.5
 bs_fc    60       89260   3746          23.8
 d_fc     180      267960  3746          71.5
 d_fc_    180      267960  3746          71.5
 db_fc    60       89260   3746          23.8
 db_fc_   60       89260   3746          23.8
 deviance 200      255800  3746          68.3
 dm_fc    990      1039610 3746         278.0
 dm_fc_   990      1039610 3746         278.0
 lb_fc    60       89260   3746          23.8
 lb_fc_   60       89260   3746          23.8
 lm_fc    990      1039610 3746         278.0
 lm_fc_   990      1039610 3746         278.0
 ms_con   10       41710   3746          11.1
 ms_fc    990      968880  3746         259.0
 orb_fc   60       90560   3746          24.2
 orm_fc   960      1050720 3746         280.0


[[2]]

Quantile (q) = 0.025
Accuracy (r) = +/- 0.005
Probability (s) = 0.95

          Burn-in  Total   Lower bound  Dependence
          (M)      (N)     (Nmin)       factor (I)
 bs_con   20       38760   3746          10.3
 bs_fc    80       97620   3746          26.1
 d_fc     120      138270  3746          36.9
 d_fc_    120      138270  3746          36.9
 db_fc    80       97600   3746          26.1
 db_fc_   80       97600   3746          26.1
 deviance 180      261540  3746          69.8
 dm_fc    1200     1454600 3746         388.0
 dm_fc_   1200     1454600 3746         388.0
 lb_fc    80       97600   3746          26.1
 lb_fc_   80       97600   3746          26.1
 lm_fc    1200     1454600 3746         388.0
 lm_fc_   1200     1454600 3746         388.0
 ms_con   10       40440   3746          10.8
 ms_fc    1200     1289400 3746         344.0
 orb_fc   60       91300   3746          24.4
 orm_fc   900      932100  3746         249.0
```

```
Iterations = 10001:509991
Thinning interval = 10
Number of chains = 2
Sample size per chain = 50000

1. Empirical mean and standard deviation for each variable,
   plus standard error of the mean:

               Mean          SD    Naive SE Time-series SE
bs_con   50862.9402    54.46801   0.1722430      0.1744591
bs_fc    51338.3597   223.89290   0.7080115      1.4048950
d_fc     37590.2028 20198.56425  63.8734685    236.9286552
d_fc_    75180.4056 40397.12850 127.7469370    473.8573103
db_fc    40886.0763 18860.69058  59.6427405    119.9372858
db_fc_   81772.1526 37721.38116 119.2854809    239.8745716
deviance   258.4367    64.14191   0.2028345      0.2908747
dm_fc    -3295.8735  7298.99468  23.0814478    241.7167825
dm_fc_   -6591.7470 14597.98936  46.1628956    483.4335650
lb_fc      475.4195   219.31036   0.6935202      1.3946196
lb_fc_     950.8390   438.62071   1.3870405      2.7892392
lm_fc      -61.8711   137.01886   0.4332917      4.5375780
lm_fc_    -123.7422   274.03772   0.8665834      9.0751561
ms_con   53782.1179    14.78296   0.0467478      0.0465820
ms_fc    53720.2468   138.29911   0.4373402      4.5709052
orb_fc       0.8409     0.07287   0.0002304      0.0004641
orm_fc       1.2869     0.63451   0.0020065      0.0215007

2. Quantiles for each variable:

                2.5%         25%       50%        75%       97.5%
bs_con    50756.0000  50826.0000 50863.000  50900.000  50969.0000
bs_fc     50873.5789  51193.7442 51346.994  51494.207  51750.8933
d_fc      -4641.7638  24695.1064 38498.852  51591.503  74617.9801
d_fc_     -9283.5275  49390.2129 76997.703 103183.005 149235.9602
db_fc      1982.1025  28624.4984 41544.463  53944.712  75674.7206
db_fc_     3964.2050  57248.9968 83088.927 107889.423 151349.4412
deviance    251.1071    254.6414   257.322    260.585    269.1764
dm_fc    -21134.6871  -6462.9401 -1728.280   1628.723   5950.4359
dm_fc_   -42269.3742 -12925.8801 -3456.560   3257.446  11900.8718
lb_fc        23.0477    332.8430   483.075    627.264    879.9386
lb_fc_       46.0954    665.6860   966.150   1254.528   1759.8772
lm_fc      -396.7465   -121.3242   -32.444     30.575    111.7033
lm_fc_     -793.4930   -242.6484   -64.888     61.150    223.4066
ms_con    53753.0000  53772.0000 53782.000  53792.000  53811.0000
ms_fc     53383.7752  53660.0125 53749.854  53814.128  53894.7242
orb_fc        0.7073      0.7902     0.838      0.888      0.9922
orm_fc        0.4877      0.8587     1.150      1.564      2.8393

quartz
     2

Cost-effectiveness analysis summary

Reference intervention: Facility & Community Interventions
Comparator intervention: do nothing

Optimal decision: choose do nothing for k<150 and Facility & Community Interventions for k>=150


Analysis for willingness to pay parameter k = 780

                                   Expected utility
do nothing                               5646565298
Facility & Community Interventions       5670415155

                                                       EIB    CEAC   ICER
Facility & Community Interventions vs do nothing 23849857 0.92922 145.53

Optimal intervention (max expected utility) for k=780: Facility & Community Interventions

EVPI 598177
quartz
     2

Cost-effectiveness analysis summary

Reference intervention: Community Intervention
Comparator intervention(s): Facility Intervention
                          : FICI

Optimal decision: choose Community Intervention for k<295 and FICI for k>=295


Analysis for willingness to pay parameter k = 780
```

```
                       Expected utility
Community Intervention      11331495730
Facility Intervention       11276901409
FICI                        11340830310

                                                     EIB    CEAC     ICER
Community Intervention vs Facility Intervention 54594321 0.94599  -3.4934
Community Intervention vs FICI                  -9334580 0.39826 292.2212

Optimal intervention (max expected utility) for k=780: FICI

EVPI 11708726
quartz
     2

Cost-effectiveness analysis summary

Reference intervention: Facility Intervention
Comparator intervention(s): Community Intervention
                          : FICI

Optimal decision: choose Community Intervention for k<295 and FICI for k>=295


Analysis for willingness to pay parameter k = 780

                       Expected utility
Community Intervention      11331495730
Facility Intervention       11276901409
FICI                        11340830310

                                                      EIB    CEAC    ICER
Facility Intervention vs Community Intervention -54594321 0.05401 -3.4934
Facility Intervention vs FICI                   -63928901 0.06219 60.2222

Optimal intervention (max expected utility) for k=780: FICI

EVPI 11708726
quartz
     2

Cost-effectiveness analysis summary

Reference intervention: FICI
Comparator intervention(s): Community Intervention
                          : Facility Intervention

Optimal decision: choose Community Intervention for k<295 and FICI for k>=295


Analysis for willingness to pay parameter k = 780

                       Expected utility
Community Intervention      11331495730
Facility Intervention       11276901409
FICI                        11340830310

                                    EIB    CEAC    ICER
FICI vs Community Intervention  9334580 0.60174 292.221
FICI vs Facility Intervention  63928901 0.93781  60.222

Optimal intervention (max expected utility) for k=780: FICI

EVPI 11708726
```

```
# MaiKhanda_LLE_log_.txt  output from MaiKhanda_final local life expectancy.R   3rd Dec 2014
12:22

The downloaded binary packages are in
        /var/folders/ps/63xh8zd90wbdcl2v8l10_npc0000gq/T//RtmpBaqeiP/downloaded_packages

The downloaded binary packages are in
        /var/folders/ps/63xh8zd90wbdcl2v8l10_npc0000gq/T//RtmpBaqeiP/downloaded_packages
Compiling model graph
   Resolving undeclared variables
   Allocating nodes
   Graph Size: 702

Initializing model

Inference for Bugs model at "/Users/timothycolbourn/Documents/Papers/MaiKhanda CEA paper/JAGS
revised/MKmodel_final_LLE.txt", fit using jags,
 2 chains, each with 510000 iterations (first 10000 discarded), n.thin = 10
 n.sims = 100000 iterations saved
            mu.vect   sd.vect       2.5%        50%      97.5%  Rhat  n.eff
bs_c     102352.341   331.911 101674.022 102362.440 102972.802 1.001  25000
bs_f     101621.975   366.363 100878.279 101631.773 102306.900 1.001  88000
bs_nc    101580.265    77.680 101427.000 101580.000 101733.000 1.001  49000
bs_nf    101330.257    79.035 101175.000 101331.000 101485.000 1.001 100000
d_c       35285.654 15001.749   4752.910  35746.440  63440.101 1.001   4700
d_f       10212.566 16795.611 -24133.017  10662.580  41678.647 1.001  48000
db_c      34743.418 14632.151   4874.324  35195.247  62263.601 1.001  18000
db_f      13127.297 16129.191 -19746.894  13522.160  43374.305 1.001  89000
dm_c        542.237  3363.687  -7339.775   1007.084   5761.899 1.003    940
dm_f      -2914.732  4580.281 -13964.244  -2189.957   3956.138 1.003   1200
lb_c        772.076   325.159    108.318    782.117   1383.636 1.001  18000
lb_f        291.718   358.426   -438.820    300.492    963.873 1.001  89000
lm_c         19.297   119.704   -261.202     35.839    205.050 1.003    940
lm_f       -103.727   162.999   -496.948    -77.934    140.788 1.003   1200
ms_c     107606.522   121.252 107322.105 107623.268 107794.310 1.003    980
ms_f     107470.139   164.833 107072.305 107496.211 107716.739 1.003   1200
ms_nc    107587.225    20.248 107547.000 107587.000 107627.000 1.001 100000
ms_nf    107573.866    20.582 107533.000 107574.000 107614.000 1.001  51000
orb_c         0.873     0.053      0.774      0.871      0.982 1.001  18000
orb_f         0.954     0.057      0.847      0.952      1.071 1.001  94000
orm_c         0.953     0.291      0.504      0.913      1.636 1.003    950
orm_f         1.245     0.385      0.669      1.184      2.178 1.002   1500
deviance    509.134   103.082    500.590    507.722    520.363 1.001  65000

For each parameter, n.eff is a crude measure of effective sample size,
and Rhat is the potential scale reduction factor (at convergence, Rhat=1).

DIC info (using the rule, pD = var(deviance)/2)
pD = 5313.0 and DIC = 5822.1
DIC is an estimate of expected predictive error (lower deviance is better).
[[1]]

Quantile (q) = 0.025
Accuracy (r) = +/- 0.005
Probability (s) = 0.95

          Burn-in  Total   Lower bound  Dependence
          (M)      (N)     (Nmin)       factor (I)
 bs_c     60       90360   3746          24.1
 bs_f     80       95060   3746          25.4
 bs_nc    20       37790   3746          10.1
 bs_nf    20       38550   3746          10.3
 d_c      90       130140  3746          34.7
 d_f      80       98720   3746          26.4
 db_c     60       91540   3746          24.4
 db_f     80       97460   3746          26.0
 deviance 180      282120  3746          75.3
 dm_c     1100     1105390 3746         295.0
 dm_f     1200     1298040 3746         347.0
 lb_c     60       91540   3746          24.4
 lb_f     80       97460   3746          26.0
 lm_c     1100     1105390 3746         295.0
 lm_f     1200     1298040 3746         347.0
 ms_c     1200     1396560 3746         373.0
 ms_f     1080     1221840 3746         326.0
 ms_nc    20       41140   3746          11.0
 ms_nf    20       37820   3746          10.1
 orb_c    60       91300   3746          24.4
 orb_f    60       87880   3746          23.5
 orm_c    1210     1363340 3746         364.0
 orm_f    880      1016730 3746         271.0


[[2]]
```

Quantile (q) = 0.025
Accuracy (r) = +/- 0.005
Probability (s) = 0.95

	Burn-in (M)	Total (N)	Lower bound (Nmin)	Dependence factor (I)
bs_c	60	86760	3746	23.2
bs_f	80	93280	3746	24.9
bs_nc	10	37930	3746	10.1
bs_nf	10	38120	3746	10.2
d_c	80	90520	3746	24.2
d_f	80	98180	3746	26.2
db_c	60	88860	3746	23.7
db_f	80	94400	3746	25.2
deviance	240	296100	3746	79.0
dm_c	1120	1194200	3746	319.0
dm_f	990	1028520	3746	275.0
lb_c	60	88860	3746	23.7
lb_f	80	94400	3746	25.2
lm_c	1120	1194200	3746	319.0
lm_f	990	1028520	3746	275.0
ms_c	910	959530	3746	256.0
ms_f	990	1084680	3746	290.0
ms_nc	20	40290	3746	10.8
ms_nf	20	40510	3746	10.8
orb_c	60	84420	3746	22.5
orb_f	60	89700	3746	23.9
orm_c	1080	1168320	3746	312.0
orm_f	1040	1110850	3746	297.0

Iterations = 10001:509991
Thinning interval = 10
Number of chains = 2
Sample size per chain = 50000

1. Empirical mean and standard deviation for each variable,
   plus standard error of the mean:

	Mean	SD	Naive SE	Time-series SE
bs_c	1.024e+05	3.319e+02	1.050e+00	2.090e+00
bs_f	1.016e+05	3.664e+02	1.159e+00	2.295e+00
bs_nc	1.016e+05	7.768e+01	2.456e-01	2.465e-01
bs_nf	1.013e+05	7.904e+01	2.499e-01	2.489e-01
d_c	3.529e+04	1.500e+04	4.744e+01	1.209e+02
d_f	1.021e+04	1.680e+04	5.311e+01	1.562e+02
db_c	3.474e+04	1.463e+04	4.627e+01	9.357e+01
db_f	1.313e+04	1.613e+04	5.100e+01	1.029e+02
deviance	5.091e+02	1.031e+02	3.260e-01	4.700e-01
dm_c	5.422e+02	3.364e+03	1.064e+01	1.053e+02
dm_f	-2.915e+03	4.580e+03	1.448e+01	1.446e+02
lb_c	7.721e+02	3.252e+02	1.028e+00	2.079e+00
lb_f	2.917e+02	3.584e+02	1.133e+00	2.287e+00
lm_c	1.930e+01	1.197e+02	3.785e-01	3.748e+00
lm_f	-1.037e+02	1.630e+02	5.154e-01	5.144e+00
ms_c	1.076e+05	1.213e+02	3.834e-01	3.685e+00
ms_f	1.075e+05	1.648e+02	5.212e-01	5.148e+00
ms_nc	1.076e+05	2.025e+01	6.403e-02	6.380e-02
ms_nf	1.076e+05	2.058e+01	6.509e-02	6.509e-02
orb_c	8.733e-01	5.304e-02	1.677e-04	3.394e-04
orb_f	9.537e-01	5.698e-02	1.802e-04	3.637e-04
orm_c	9.534e-01	2.911e-01	9.204e-04	8.926e-03
orm_f	1.245e+00	3.848e-01	1.217e-03	1.210e-02

2. Quantiles for each variable:

	2.5%	25%	50%	75%	97.5%
bs_c	1.017e+05	1.021e+05	1.024e+05	1.026e+05	1.030e+05
bs_f	1.009e+05	1.014e+05	1.016e+05	1.019e+05	1.023e+05
bs_nc	1.014e+05	1.015e+05	1.016e+05	1.016e+05	1.017e+05
bs_nf	1.012e+05	1.013e+05	1.013e+05	1.014e+05	1.015e+05
d_c	4.753e+03	2.538e+04	3.575e+04	4.559e+04	6.344e+04
d_f	-2.413e+04	-8.719e+02	1.066e+04	2.181e+04	4.168e+04
db_c	4.874e+03	2.510e+04	3.520e+04	4.477e+04	6.226e+04
db_f	-1.975e+04	2.563e+03	1.352e+04	2.422e+04	4.337e+04
deviance	5.006e+02	5.048e+02	5.077e+02	5.114e+02	5.204e+02
dm_c	-7.340e+03	-1.341e+03	1.007e+03	2.936e+03	5.762e+03
dm_f	-1.396e+04	-5.364e+03	-2.190e+03	3.369e+02	3.956e+03
lb_c	1.083e+02	5.577e+02	7.821e+02	9.949e+02	1.384e+03
lb_f	-4.388e+02	5.696e+01	3.005e+02	5.382e+02	9.639e+02
lm_c	-2.612e+02	-4.772e+01	3.584e+01	1.045e+02	2.050e+02
lm_f	-4.969e+02	-1.909e+02	-7.793e+01	1.199e+01	1.408e+02

```
ms_c      1.073e+05  1.075e+05  1.076e+05 1.077e+05 1.078e+05
ms_f      1.071e+05  1.074e+05  1.075e+05 1.076e+05 1.077e+05
ms_nc     1.075e+05  1.076e+05  1.076e+05 1.076e+05 1.076e+05
ms_nf     1.075e+05  1.076e+05  1.076e+05 1.076e+05 1.076e+05
orb_c     7.742e-01  8.368e-01  8.714e-01 9.081e-01 9.821e-01
orb_f     8.474e-01  9.144e-01  9.521e-01 9.909e-01 1.071e+00
orm_c     5.040e-01  7.454e-01  9.128e-01 1.116e+00 1.636e+00
orm_f     6.688e-01  9.717e-01  1.184e+00 1.452e+00 2.178e+00

quartz
     2

Cost-effectiveness analysis summary

Reference intervention: Community Intervention
Comparator intervention: do nothing

Optimal decision: choose do nothing for k<155 and Community Intervention for k>=155


Analysis for willingness to pay parameter k = 780

                       Expected utility
do nothing                   5923564111
Community Intervention       5945738130

                                          EIB    CEAC   ICER
Community Intervention vs do nothing 22174019 0.96623 151.59

Optimal intervention (max expected utility) for k=780: Community Intervention

EVPI 170677
quartz
     2

Cost-effectiveness analysis summary

Reference intervention: Facility Intervention
Comparator intervention: do nothing

Optimal decision: choose do nothing for k<550 and Facility Intervention for k>=550


Analysis for willingness to pay parameter k = 780

                      Expected utility
do nothing                  5914496043
Facility Intervention       5916869632

                                        EIB    CEAC   ICER
Facility Intervention vs do nothing 2373589 0.58166 547.58

Optimal intervention (max expected utility) for k=780: Facility Intervention

EVPI 4144723
quartz
     2

Cost-effectiveness analysis summary

Reference intervention: Community Intervention
Comparator intervention: Facility Intervention

Community Intervention dominates for all k in [0 - 2500]


Analysis for willingness to pay parameter k = 780

                       Expected utility
Community Intervention       5945738130
Facility Intervention        5916869632

                                                     EIB    CEAC    ICER
Community Intervention vs Facility Intervention 28868498 0.94837 -6.6329

Optimal intervention (max expected utility) for k=780: Community Intervention

EVPI 383986
Compiling model graph
   Resolving undeclared variables
   Allocating nodes
   Graph Size: 357

Initializing model
```

```
Inference for Bugs model at "/Users/timothycolbourn/Documents/Papers/MaiKhanda CEA paper/JAGS
revised/MKmodelFC_final_LLE.txt", fit using jags,
 2 chains, each with 510000 iterations (first 10000 discarded), n.thin = 10
 n.sims = 100000 iterations saved
           mu.vect   sd.vect       2.5%       50%     97.5%  Rhat  n.eff
bs_con   50862.710    54.501  50756.000 50863.000 50969.000 1.001 100000
bs_fc    51338.166   224.031  50873.106 51346.741 51748.058 1.001 100000
d_fc     19738.609 10538.349  -2049.754 20219.078 38975.523 1.001  41000
d_fc_    39477.217 21076.698  -4099.509 40438.156 77951.046 1.001  41000
db_fc    21395.535  9873.907    982.407 21794.699 39603.014 1.001 100000
db_fc_   42791.069 19747.813   1964.815 43589.398 79206.028 1.001 100000
dm_fc    -1656.926  3648.879 -10858.291  -914.776  3185.935 1.004   2500
dm_fc_   -3313.852  7297.758 -21716.583 -1829.552  6371.871 1.004   2500
lb_fc      475.456   219.420     21.831   484.327   880.067 1.001 100000
lb_fc_     950.913   438.840     43.663   968.653  1760.134 1.001 100000
lm_fc      -58.965   129.853   -386.416   -32.554   113.378 1.004   2500
lm_fc_    -117.931   259.707   -772.832   -65.109   226.757 1.004   2500
ms_con   53782.154    14.741  53753.000 53782.000 53810.000 1.001  63000
ms_fc    53723.189   131.158  53391.857 53749.976 53896.268 1.003   2600
orb_fc       0.841     0.073      0.708     0.838     0.993 1.001 100000
orm_fc       1.273     0.600      0.482     1.150     2.794 1.001   7000
deviance   258.836   118.929    251.181   257.482   269.122 1.001   5700

For each parameter, n.eff is a crude measure of effective sample size,
and Rhat is the potential scale reduction factor (at convergence, Rhat=1).

DIC info (using the rule, pD = var(deviance)/2)
pD = 7072.1 and DIC = 7330.9
DIC is an estimate of expected predictive error (lower deviance is better).
[[1]]

Quantile (q) = 0.025
Accuracy (r) = +/- 0.005
Probability (s) = 0.95

          Burn-in  Total   Lower bound  Dependence
          (M)      (N)     (Nmin)       factor (I)
 bs_con   20       38400   3746          10.3
 bs_fc    60       89460   3746          23.9
 d_fc     120      187840  3746          50.1
 d_fc_    120      187840  3746          50.1
 db_fc    80       90000   3746          24.0
 db_fc_   80       90000   3746          24.0
 deviance 180      242460  3746          64.7
 dm_fc    990      1044340 3746         279.0
 dm_fc_   990      1044340 3746         279.0
 lb_fc    80       90000   3746          24.0
 lb_fc_   80       90000   3746          24.0
 lm_fc    990      1044340 3746         279.0
 lm_fc_   990      1044340 3746         279.0
 ms_con   20       41690   3746          11.1
 ms_fc    990      1024760 3746         274.0
 orb_fc   60       87540   3746          23.4
 orm_fc   1080     1238220 3746         331.0


[[2]]

Quantile (q) = 0.025
Accuracy (r) = +/- 0.005
Probability (s) = 0.95

          Burn-in  Total   Lower bound  Dependence
          (M)      (N)     (Nmin)       factor (I)
 bs_con   20       37610   3746          10.0
 bs_fc    60       86400   3746          23.1
 d_fc     120      139470  3746          37.2
 d_fc_    120      139470  3746          37.2
 db_fc    60       87580   3746          23.4
 db_fc_   60       87580   3746          23.4
 deviance 160      195520  3746          52.2
 dm_fc    1200     1289520 3746         344.0
 dm_fc_   1200     1289520 3746         344.0
 lb_fc    60       87580   3746          23.4
 lb_fc_   60       87580   3746          23.4
 lm_fc    1200     1289520 3746         344.0
 lm_fc_   1200     1289520 3746         344.0
 ms_con   20       42020   3746          11.2
 ms_fc    1300     1450930 3746         387.0
 orb_fc   80       93480   3746          25.0
 orm_fc   990      1044340 3746         279.0
```

```
Iterations = 10001:509991
Thinning interval = 10
Number of chains = 2
Sample size per chain = 50000

1. Empirical mean and standard deviation for each variable,
   plus standard error of the mean:

               Mean         SD   Naive SE Time-series SE
bs_con   50862.7095    54.5009  0.1723471      0.1707505
bs_fc    51338.1658   224.0307  0.7084472      1.4103686
d_fc     19738.6087 10538.3488 33.3251851    122.0902297
d_fc_    39477.2174 21076.6977 66.6503703    244.1804594
db_fc    21395.5346  9873.9067 31.2240347     63.2247828
db_fc_   42791.0692 19747.8135 62.4480693    126.4495656
deviance   258.8364   118.9290  0.3760866      0.4633666
dm_fc    -1656.9259  3648.8792 11.5387691    120.3292760
dm_fc_   -3313.8517  7297.7584 23.0775382    240.6585520
lb_fc      475.4563   219.4201  0.6938674      1.4049952
lb_fc_     950.9126   438.8403  1.3877349      2.8099903
lm_fc      -58.9653   129.8534  0.4106324      4.2821806
lm_fc_    -117.9307   259.7067  0.8212647      8.5643613
ms_con   53782.1539    14.7411  0.0466154      0.0467904
ms_fc    53723.1886   131.1580  0.4147580      4.2651317
orb_fc       0.8409     0.0729  0.0002305      0.0004673
orm_fc       1.2734     0.6000  0.0018974      0.0196008

2. Quantiles for each variable:

                2.5%        25%        50%       75%      97.5%
bs_con    50756.0000 50826.0000 50863.0000 50899.000 50969.0000
bs_fc     50873.1057 51192.8322 51346.7408 51494.565 51748.0582
d_fc      -2049.7544 12937.2223 20219.0779 27065.169 38975.5228
d_fc_     -4099.5089 25874.4447 40438.1558 54130.338 77951.0456
db_fc       982.4074 14980.6830 21794.6989 28283.757 39603.0142
db_fc_     1964.8149 29961.3660 43589.3978 56567.513 79206.0285
deviance    251.1813   254.7882   257.4819   260.805   269.1220
dm_fc    -10858.2915 -3355.3934  -914.7762   876.682  3185.9354
dm_fc_   -21716.5830 -6710.7868 -1829.5525  1753.365  6371.8707
lb_fc        21.8313   332.9041   484.3266   628.528   880.0670
lb_fc_       43.6626   665.8081   968.6533  1257.056  1760.1340
lm_fc      -386.4161  -119.4090   -32.5543    31.199   113.3785
lm_fc_     -772.8321  -238.8180   -65.1086    62.397   226.7570
ms_con    53753.0000 53772.0000 53782.0000 53792.000 53810.0000
ms_fc     53391.8568 53661.7696 53749.9756 53814.902 53896.2684
orb_fc        0.7078     0.7900     0.8375     0.888     0.9925
orm_fc        0.4817     0.8554     1.1499     1.553     2.7940

quartz
     2

Cost-effectiveness analysis summary

Reference intervention: Facility & Community Interventions
Comparator intervention: do nothing

Optimal decision: choose do nothing for k<280 and Facility & Community Interventions for k>=280


Analysis for willingness to pay parameter k = 780

                                   Expected utility
do nothing                               2964078353
Facility & Community Interventions       2974003967

                                                     EIB    CEAC   ICER
Facility & Community Interventions vs do nothing 9925614 0.88326 277.15

Optimal intervention (max expected utility) for k=780: Facility & Community Interventions

EVPI 534291
quartz
     2

Cost-effectiveness analysis summary

Reference intervention: Community Intervention
Comparator intervention(s): Facility Intervention
                          : FICI

Optimal decision: choose Community Intervention for k<555 and FICI for k>=555
```

```
Analysis for willingness to pay parameter k = 780

                       Expected utility
Community Intervention       5945738130
Facility Intervention        5916869632
FICI                         5948007934

                                                     EIB    CEAC     ICER
Community Intervention vs Facility Intervention 28868498 0.94837  -6.6329
Community Intervention vs FICI                  -2269803 0.44973 554.8100

Optimal intervention (max expected utility) for k=780: FICI

EVPI 7212170
quartz
     2

Cost-effectiveness analysis summary

Reference intervention: Facility Intervention
Comparator intervention(s): Community Intervention
                          : FICI

Optimal decision: choose Community Intervention for k<555 and FICI for k>=555


Analysis for willingness to pay parameter k = 780

                       Expected utility
Community Intervention       5945738130
Facility Intervention        5916869632
FICI                         5948007934

                                                      EIB    CEAC     ICER
Facility Intervention vs Community Intervention -28868498 0.05163  -6.6329
Facility Intervention vs FICI                   -31138302 0.07416 114.3433

Optimal intervention (max expected utility) for k=780: FICI

EVPI 7212170
quartz
     2

Cost-effectiveness analysis summary

Reference intervention: FICI
Comparator intervention(s): Community Intervention
                          : Facility Intervention

Optimal decision: choose Community Intervention for k<555 and FICI for k>=555


Analysis for willingness to pay parameter k = 780

                       Expected utility
Community Intervention       5945738130
Facility Intervention        5916869632
FICI                         5948007934

                                    EIB    CEAC   ICER
FICI vs Community Intervention  2269803 0.55027 554.81
FICI vs Facility Intervention  31138302 0.92584 114.34

Optimal intervention (max expected utility) for k=780: FICI

EVPI 7212170
```

```
# MaiKhanda_future_final_log.txt  output from MaiKhanda_future_final.R   3rd Dec 2014 14:38

Initializing model

Inference for Bugs model at "/Users/timothycolbourn/Documents/Papers/MaiKhanda CEA paper/JAGS
revised/MKmodel_final.txt", fit using jags,
 2 chains, each with 21000 iterations (first 1000 discarded), n.thin = 2
 n.sims = 20000 iterations saved
            mu.vect   sd.vect       2.5%        50%      97.5%  Rhat n.eff
bs_c     102352.659   343.406 101661.403 102364.806 102974.464 1.002  1600
bs_f     101642.576   364.048 100913.382 101652.590 102322.593 1.001 20000
bs_nc    101580.385    77.117 101429.000 101581.000 101730.000 1.001 20000
bs_nf    101329.767    78.958 101174.000 101330.000 101485.000 1.001 20000
d_c       67298.347 29427.529   8538.188  68147.311 121191.979 1.014   120
d_f       20716.243 31641.196 -42734.637  21267.961  79527.764 1.001 20000
db_c      66415.528 28949.779   7855.385  67343.667 119294.148 1.002  1500
db_f      26901.607 30614.663 -34747.922  27731.431  84027.008 1.001 20000
dm_c        882.819  6334.383 -12815.524   2235.752  11123.963 1.206    12
dm_f      -6185.364  9067.853 -26875.173  -5220.987   9104.277 1.001  3400
lb_c        772.274   336.625     91.342    783.066   1387.141 1.002  1500
lb_f        312.809   355.984   -404.046    322.459    977.058 1.001 20000
lm_c         16.573   118.911   -240.577     41.970    208.822 1.206    12
lm_f       -116.113   170.224   -504.509    -98.010    170.908 1.001  3400
ms_c     107603.759   120.510 107340.560 107628.444 107796.684 1.200    12
ms_f     107457.681   172.146 107063.392 107477.079 107744.663 1.001  3800
ms_nc    107587.186    20.354 107547.000 107587.000 107626.000 1.001 20000
ms_nf    107573.795    20.677 107532.000 107574.000 107614.000 1.001 20000
orb_c         0.873     0.055      0.773      0.871      0.985 1.002  1500
orb_f         0.950     0.057      0.845      0.949      1.065 1.001 20000
orm_c         0.960     0.289      0.491      0.897      1.588 1.191    12
orm_f         1.274     0.401      0.596      1.231      2.195 1.006  1500
deviance    520.565   358.490    500.567    508.428    527.019 1.001  3800

For each parameter, n.eff is a crude measure of effective sample size,
and Rhat is the potential scale reduction factor (at convergence, Rhat=1).

DIC info (using the rule, pD = var(deviance)/2)
pD = 64259.6 and DIC = 64780.1
DIC is an estimate of expected predictive error (lower deviance is better).
[[1]]

Quantile (q) = 0.025
Accuracy (r) = +/- 0.005
Probability (s) = 0.95

          Burn-in  Total  Lower bound  Dependence
          (M)      (N)    (Nmin)       factor (I)
 bs_c     48       45760  3746          12.20
 bs_f     40       43320  3746          11.60
 bs_nc    4        7508   3746           2.00
 bs_nf    4        7622   3746           2.03
 d_c      48       47814  3746          12.80
 d_f      40       47800  3746          12.80
 db_c     44       43108  3746          11.50
 db_f     36       38520  3746          10.30
 deviance 40       50768  3746          13.60
 dm_c     216      248166 3746          66.20
 dm_f     224      249584 3746          66.60
 lb_c     44       43108  3746          11.50
 lb_f     36       38520  3746          10.30
 lm_c     216      248166 3746          66.20
 lm_f     224      249584 3746          66.60
 ms_c     216      241380 3746          64.40
 ms_f     176      180528 3746          48.20
 ms_nc    4        8052   3746           2.15
 ms_nf    4        7890   3746           2.11
 orb_c    50       53240  3746          14.20
 orb_f    54       65454  3746          17.50
 orm_c    272      271540 3746          72.50
 orm_f    328      374936 3746         100.00


[[2]]

Quantile (q) = 0.025
Accuracy (r) = +/- 0.005
Probability (s) = 0.95

          Burn-in  Total  Lower bound  Dependence
          (M)      (N)    (Nmin)       factor (I)
 bs_c     42       52080  3746          13.90
 bs_f     42       43542  3746          11.60
 bs_nc    4        7886   3746           2.11
 bs_nf    4        7950   3746           2.12
```

```
 d_c      42       43602  3746          11.60
 d_f      36       37626  3746          10.00
 db_c     48       49218  3746          13.10
 db_f     36       41876  3746          11.20
 deviance 48       61096  3746          16.30
 dm_c     300      369552 3746          98.70
 dm_f     228      225672 3746          60.20
 lb_c     48       49218  3746          13.10
 lb_f     36       41876  3746          11.20
 lm_c     300      369552 3746          98.70
 lm_f     228      225672 3746          60.20
 ms_c     140      145390 3746          38.80
 ms_f     408      380136 3746         101.00
 ms_nc    4        8248   3746           2.20
 ms_nf    4        8124   3746           2.17
 orb_c    42       49164  3746          13.10
 orb_f    42       40614  3746          10.80
 orm_c    402      430980 3746         115.00
 orm_f    160      167400 3746          44.70


Iterations = 1001:20999
Thinning interval = 2
Number of chains = 2
Sample size per chain = 10000

1. Empirical mean and standard deviation for each variable,
   plus standard error of the mean:

               Mean        SD  Naive SE Time-series SE
bs_c      1.024e+05 3.434e+02 2.428e+00      1.037e+01
bs_f      1.016e+05 3.640e+02 2.574e+00      1.114e+01
bs_nc     1.016e+05 7.712e+01 5.453e-01      5.327e-01
bs_nf     1.013e+05 7.896e+01 5.583e-01      5.583e-01
d_c       6.730e+04 2.943e+04 2.081e+02      9.264e+02
d_f       2.072e+04 3.164e+04 2.237e+02      1.022e+03
db_c      6.642e+04 2.895e+04 2.047e+02      8.986e+02
db_f      2.690e+04 3.061e+04 2.165e+02      9.489e+02
deviance  5.206e+02 3.585e+02 2.535e+00      6.547e+00
dm_c      8.828e+02 6.334e+03 4.479e+01      8.641e+02
dm_f     -6.185e+03 9.068e+03 6.412e+01      1.400e+03
lb_c      7.723e+02 3.366e+02 2.380e+00      1.045e+01
lb_f      3.128e+02 3.560e+02 2.517e+00      1.103e+01
lm_c      1.657e+01 1.189e+02 8.408e-01      1.622e+01
lm_f     -1.161e+02 1.702e+02 1.204e+00      2.628e+01
ms_c      1.076e+05 1.205e+02 8.521e-01      1.630e+01
ms_f      1.075e+05 1.721e+02 1.217e+00      2.543e+01
ms_nc     1.076e+05 2.035e+01 1.439e-01      1.439e-01
ms_nf     1.076e+05 2.068e+01 1.462e-01      1.505e-01
orb_c     8.732e-01 5.513e-02 3.898e-04      1.693e-03
orb_f     9.504e-01 5.653e-02 3.997e-04      1.756e-03
orm_c     9.600e-01 2.888e-01 2.042e-03      3.876e-02
orm_f     1.274e+00 4.014e-01 2.838e-03      6.203e-02

2. Quantiles for each variable:

               2.5%        25%        50%       75%     97.5%
bs_c      1.017e+05  1.021e+05  1.024e+05 1.026e+05 1.030e+05
bs_f      1.009e+05  1.014e+05  1.017e+05 1.019e+05 1.023e+05
bs_nc     1.014e+05  1.015e+05  1.016e+05 1.016e+05 1.017e+05
bs_nf     1.012e+05  1.013e+05  1.013e+05 1.014e+05 1.015e+05
d_c       8.538e+03  4.854e+04  6.815e+04 8.709e+04 1.212e+05
d_f      -4.273e+04  1.053e+02  2.127e+04 4.210e+04 7.953e+04
db_c      7.855e+03  4.836e+04  6.734e+04 8.554e+04 1.193e+05
db_f     -3.475e+04  6.991e+03  2.773e+04 4.768e+04 8.403e+04
deviance  5.006e+02  5.049e+02  5.084e+02 5.127e+02 5.270e+02
dm_c     -1.282e+04 -3.169e+03  2.236e+03 5.369e+03 1.112e+04
dm_f     -2.688e+04 -1.192e+04 -5.221e+03 6.142e+02 9.104e+03
lb_c      9.134e+01  5.623e+02  7.831e+02 9.946e+02 1.387e+03
lb_f     -4.040e+02  8.129e+01  3.225e+02 5.544e+02 9.771e+02
lm_c     -2.406e+02 -5.948e+01  4.197e+01 1.008e+02 2.088e+02
lm_f     -5.045e+02 -2.238e+02 -9.801e+01 1.153e+01 1.709e+02
ms_c      1.073e+05  1.075e+05  1.076e+05 1.077e+05 1.078e+05
ms_f      1.071e+05  1.073e+05  1.075e+05 1.076e+05 1.077e+05
ms_nc     1.075e+05  1.076e+05  1.076e+05 1.076e+05 1.076e+05
ms_nf     1.075e+05  1.076e+05  1.076e+05 1.076e+05 1.076e+05
orb_c     7.734e-01  8.367e-01  8.712e-01 9.074e-01 9.849e-01
orb_f     8.455e-01  9.119e-01  9.486e-01 9.870e-01 1.065e+00
orm_c     4.908e-01  7.555e-01  8.975e-01 1.144e+00 1.588e+00
orm_f     5.958e-01  9.728e-01  1.231e+00 1.531e+00 2.195e+00

quartz
     2
```

```
Cost-effectiveness analysis summary

Reference intervention: Community Intervention
Comparator intervention: do nothing

Optimal decision: choose do nothing for k<80 and Community Intervention for k>=80


Analysis for willingness to pay parameter k = 780

                       Expected utility
do nothing                  11284324399
Community Intervention      11331468318

                                          EIB    CEAC   ICER
Community Intervention vs do nothing 47143920 0.97835 79.479

Optimal intervention (max expected utility) for k=780: Community Intervention

EVPI 285672
quartz
     2

Cost-effectiveness analysis summary

Reference intervention: Facility Intervention
Comparator intervention: do nothing

Optimal decision: choose do nothing for k<270 and Facility Intervention for k>=270


Analysis for willingness to pay parameter k = 780

                      Expected utility
do nothing                 11266956465
Facility Intervention      11277522922

                                         EIB    CEAC   ICER
Facility Intervention vs do nothing 10566457 0.67495 269.94

Optimal intervention (max expected utility) for k=780: Facility Intervention

EVPI 5440834
quartz
     2

Cost-effectiveness analysis summary

Reference intervention: Community Intervention
Comparator intervention: Facility Intervention

Community Intervention dominates for all k in [0 - 2500]


Analysis for willingness to pay parameter k = 780

                       Expected utility
Community Intervention      11331468318
Facility Intervention       11277522922

                                                     EIB    CEAC    ICER
Community Intervention vs Facility Intervention 53945396 0.94415 -3.5356

Optimal intervention (max expected utility) for k=780: Community Intervention

EVPI 903141

Initializing model

Inference for Bugs model at "/Users/timothycolbourn/Documents/Papers/MaiKhanda CEA paper/JAGS
revised/MKmodelFC_final.txt", fit using jags,
 2 chains, each with 21000 iterations (first 1000 discarded), n.thin = 2
 n.sims = 20000 iterations saved
           mu.vect   sd.vect       2.5%       50%     97.5%  Rhat n.eff
bs_con   50862.986    54.260  50756.000 50863.000 50970.000 1.001 20000
bs_fc    51342.036   224.311  50894.180 51349.031 51754.583 1.001 20000
d_fc     38986.900 20133.229  -1761.312 39646.226 75428.855 1.008  5700
db_fc    41198.284 18868.364   3537.467 41636.420 75926.881 1.001 15000
dm_fc    -2211.385  6632.355 -19802.372  -792.271  6448.254 1.083   250
lb_fc      479.050   219.400     41.133   484.144   882.871 1.001 15000
lm_fc      -41.513   124.505   -371.736   -14.873   121.049 1.083   250
ms_con   53782.142    14.881  53753.000 53782.000 53811.000 1.001 20000
ms_fc    53740.629   125.852  53409.374 53767.722 53904.154 1.081   260
```

```
orb_fc       0.840     0.073      0.706     0.838     0.986 1.001 12000
orm_fc       1.192     0.575      0.447     1.069     2.739 1.032 20000
deviance   274.238   684.960    251.663   258.148   271.404 1.005 19000

For each parameter, n.eff is a crude measure of effective sample size,
and Rhat is the potential scale reduction factor (at convergence, Rhat=1).

DIC info (using the rule, pD = var(deviance)/2)
pD = 234593.1 and DIC = 234867.4
DIC is an estimate of expected predictive error (lower deviance is better).
[[1]]

Quantile (q) = 0.025
Accuracy (r) = +/- 0.005
Probability (s) = 0.95

          Burn-in  Total  Lower bound  Dependence
          (M)      (N)    (Nmin)       factor (I)
 bs_con   4        7604   3746          2.03
 bs_fc    40       49416  3746         13.20
 d_fc     48       50352  3746         13.40
 db_fc    36       41876  3746         11.20
 deviance 72       73504  3746         19.60
 dm_fc    260      237692 3746         63.50
 lb_fc    36       41876  3746         11.20
 lm_fc    260      237692 3746         63.50
 ms_con   4        8630   3746          2.30
 ms_fc    280      316940 3746         84.60
 orb_fc   56       59904  3746         16.00
 orm_fc   264      293670 3746         78.40


[[2]]

Quantile (q) = 0.025
Accuracy (r) = +/- 0.005
Probability (s) = 0.95

          Burn-in  Total  Lower bound  Dependence
          (M)      (N)    (Nmin)       factor (I)
 bs_con   2        7800   3746           2.08
 bs_fc    28       29976  3746           8.00
 d_fc     36       35130  3746           9.38
 db_fc    36       36876  3746           9.84
 deviance 48       65472  3746          17.50
 dm_fc    352      433598 3746         116.00
 lb_fc    36       36876  3746           9.84
 lm_fc    352      433598 3746         116.00
 ms_con   4        8078   3746           2.16
 ms_fc    308      380842 3746         102.00
 orb_fc   48       53016  3746          14.20
 orm_fc   224      242196 3746          64.70



Iterations = 1001:20999
Thinning interval = 2
Number of chains = 2
Sample size per chain = 10000

1. Empirical mean and standard deviation for each variable,
   plus standard error of the mean:

               Mean          SD    Naive SE Time-series SE
bs_con   50862.9863    54.25979   0.3836746       0.383677
bs_fc    51342.0361   224.31077   1.5861167       6.687838
d_fc     38986.8997 20133.22916 142.3634287     733.900825
db_fc    41198.2844 18868.36368 133.4194791     575.676426
deviance   274.2380   684.95976   4.8433969       9.588212
dm_fc    -2211.3847  6632.35549  46.8978354    1037.517544
lb_fc      479.0498   219.39958   1.5513893       6.693912
lm_fc      -41.5128   124.50451   0.8803799      19.476582
ms_con   53782.1420    14.88095   0.1052242       0.105225
ms_fc    53740.6292   125.85151   0.8899046      19.973687
orb_fc       0.8397     0.07318   0.0005175       0.002215
orm_fc       1.1925     0.57523   0.0040675       0.092813

2. Quantiles for each variable:

                2.5%        25%        50%        75%      97.5%
bs_con    50756.0000 50827.0000 50863.0000 50899.0000 50970.0000
bs_fc     50894.1796 51200.1761 51349.0309 51491.9897 51754.5831
d_fc      -1761.3122 26414.6354 39646.2265 52536.0328 75428.8549
db_fc      3537.4673 29204.5867 41636.4200 53689.1623 75926.8810
```

```
deviance    251.6627   255.3545   258.1476   261.4310   271.4043
dm_fc    -19802.3720 -5638.2032  -792.2708  2558.6247  6448.2542
lb_fc        41.1333   339.5882   484.1444   624.2926   882.8707
lm_fc      -371.7359  -105.8420   -14.8727    48.0313   121.0485
ms_con    53753.0000 53772.0000 53782.0000 53792.0000 53811.0000
ms_fc     53409.3744 53675.9711 53767.7223 53831.4781 53904.1537
orb_fc        0.7063     0.7910     0.8377     0.8857     0.9862
orm_fc        0.4472     0.7775     1.0689     1.4901     2.7391

quartz
     2

Cost-effectiveness analysis summary

Reference intervention: Facility & Community Interventions
Comparator intervention: do nothing

Optimal decision: choose do nothing for k<145 and Facility & Community Interventions for k>=145


Analysis for willingness to pay parameter k = 780

                                   Expected utility
do nothing                               5646569387
Facility & Community Interventions       5671508668

                                                        EIB    CEAC   ICER
Facility & Community Interventions vs do nothing 24939281 0.94225 140.32

Optimal intervention (max expected utility) for k=780: Facility & Community Interventions

EVPI 488886
quartz
     2

Cost-effectiveness analysis summary

Reference intervention: Community Intervention
Comparator intervention(s): Facility Intervention
                          : FICI

Optimal decision: choose Community Intervention for k<255 and FICI for k>=255


Analysis for willingness to pay parameter k = 780

                       Expected utility
Community Intervention      11331468318
Facility Intervention       11277522922
FICI                        11343017336

                                                        EIB    CEAC     ICER
Community Intervention vs Facility Intervention  53945396 0.94415  -3.5356
Community Intervention vs FICI                  -11549017 0.37850 254.4698

Optimal intervention (max expected utility) for k=780: FICI

EVPI 10716765
quartz
     2
[1] "time horizon in years"
[1] 5
[1] "cost discount rate"
[1] 0.03
[1] "effects discount rate"
[1] 0

Cost-effectiveness analysis summary

Reference intervention: Community Intervention
Comparator intervention(s): Facility Intervention
                          : FICI

Optimal decision: choose Community Intervention for k<230 and FICI for k>=230


Analysis for willingness to pay parameter k = 780

                       Expected utility
Community Intervention     315067557790
Facility Intervention      313568285502
FICI                       315406426918

                                                         EIB    CEAC     ICER
```

```
Community Intervention vs Facility Intervention 1499272288 0.99875  -3.2331
Community Intervention vs FICI                 -338869127 0.24650 225.3860

Optimal intervention (max expected utility) for k=780: FICI

EVPI 72244693
quartz
     2
[1] "time horizon in years"
[1] 5
[1] "cost discount rate"
[1] 0
[1] "effects discount rate"
[1] 0

Cost-effectiveness analysis summary

Reference intervention: Community Intervention
Comparator intervention(s): Facility Intervention
                          : FICI

Optimal decision: choose Community Intervention for k<250 and FICI for k>=250


Analysis for willingness to pay parameter k = 780

                       Expected utility
Community Intervention     315055903400
Facility Intervention      313556064029
FICI                       315382551054

                                                       EIB    CEAC     ICER
Community Intervention vs Facility Intervention 1499839371 0.99875  -3.5294
Community Intervention vs FICI                 -326647654 0.25400 245.3884

Optimal intervention (max expected utility) for k=780: FICI

EVPI 75301091
quartz
     2
[1] "time horizon in years"
[1] 5
[1] "cost discount rate"
[1] 0.1
[1] "effects discount rate"
[1] 0

Cost-effectiveness analysis summary

Reference intervention: Community Intervention
Comparator intervention(s): Facility Intervention
                          : FICI

Optimal decision: choose Community Intervention for k<190 and FICI for k>=190


Analysis for willingness to pay parameter k = 780

                       Expected utility
Community Intervention     315089433936
Facility Intervention      313591226103
FICI                       315451243664

                                                       EIB    CEAC     ICER
Community Intervention vs Facility Intervention 1498207833 0.99875  -2.6771
Community Intervention vs FICI                 -361809728 0.23225 187.8400

Optimal intervention (max expected utility) for k=780: FICI

EVPI 66762106
quartz
     2
[1] "time horizon in years"
[1] 5
[1] "cost discount rate"
[1] 0.03
[1] "effects discount rate"
[1] 0.02

Cost-effectiveness analysis summary

Reference intervention: Community Intervention
Comparator intervention(s): Facility Intervention
                          : FICI
```

```
Optimal decision: choose Community Intervention for k<240 and FICI for k>=240


Analysis for willingness to pay parameter k = 780

                       Expected utility
Community Intervention     297004097950
Facility Intervention      295590391430
FICI                       297315655188

                                                       EIB    CEAC     ICER
Community Intervention vs Facility Intervention 1413706520 0.99875  -3.4297
Community Intervention vs FICI                  -311557239 0.25155 239.0876

Optimal intervention (max expected utility) for k=780: FICI

EVPI 70073716
quartz
     2
[1] "time horizon in years"
[1] 5
[1] "cost discount rate"
[1] 0
[1] "effects discount rate"
[1] 0.02

Cost-effectiveness analysis summary

Reference intervention: Community Intervention
Comparator intervention(s): Facility Intervention
                          : FICI

Optimal decision: choose Community Intervention for k<265 and FICI for k>=265


Analysis for willingness to pay parameter k = 780

                       Expected utility
Community Intervention     296992443559
Facility Intervention      295578169957
FICI                       297291779325

                                                       EIB    CEAC    ICER
Community Intervention vs Facility Intervention 1414273602 0.99875  -3.744
Community Intervention vs FICI                  -299335766 0.25865 260.306

Optimal intervention (max expected utility) for k=780: FICI

EVPI 73193910
quartz
     2
[1] "time horizon in years"
[1] 5
[1] "cost discount rate"
[1] 0.1
[1] "effects discount rate"
[1] 0.02

Cost-effectiveness analysis summary

Reference intervention: Community Intervention
Comparator intervention(s): Facility Intervention
                          : FICI

Optimal decision: choose Community Intervention for k<200 and FICI for k>=200


Analysis for willingness to pay parameter k = 780

                       Expected utility
Community Intervention     297025974095
Facility Intervention      295613332031
FICI                       297360471934

                                                       EIB    CEAC     ICER
Community Intervention vs Facility Intervention 1412642064 0.99875  -2.8398
Community Intervention vs FICI                  -334497839 0.23610 199.2591

Optimal intervention (max expected utility) for k=780: FICI

EVPI 64479001
quartz
     2
```

```
[1] "time horizon in years"
[1] 5
[1] "cost discount rate"
[1] 0.03
[1] "effects discount rate"
[1] 0.03

Cost-effectiveness analysis summary

Reference intervention: Community Intervention
Comparator intervention(s): Facility Intervention
                          : FICI

Optimal decision: choose Community Intervention for k<250 and FICI for k>=250


Analysis for willingness to pay parameter k = 780

                       Expected utility
Community Intervention     288572376234
Facility Intervention      287198610388
FICI                       288871184738

                                                       EIB    CEAC     ICER
Community Intervention vs Facility Intervention 1373765846 0.99875  -3.5299
Community Intervention vs FICI                  -298808504 0.25405 246.0703

Optimal intervention (max expected utility) for k=780: FICI

EVPI 69079152
quartz
     2
[1] "time horizon in years"
[1] 5
[1] "cost discount rate"
[1] 0
[1] "effects discount rate"
[1] 0.03

Cost-effectiveness analysis summary

Reference intervention: Community Intervention
Comparator intervention(s): Facility Intervention
                          : FICI

Optimal decision: choose Community Intervention for k<270 and FICI for k>=270


Analysis for willingness to pay parameter k = 780

                       Expected utility
Community Intervention     288560721843
Facility Intervention      287186388915
FICI                       288847308875

                                                       EIB    CEAC     ICER
Community Intervention vs Facility Intervention 1374332929 0.99875  -3.8533
Community Intervention vs FICI                  -286587031 0.26200 267.9084

Optimal intervention (max expected utility) for k=780: FICI

EVPI 72231344
quartz
     2
[1] "time horizon in years"
[1] 5
[1] "cost discount rate"
[1] 0.1
[1] "effects discount rate"
[1] 0.03

Cost-effectiveness analysis summary

Reference intervention: Community Intervention
Comparator intervention(s): Facility Intervention
                          : FICI

Optimal decision: choose Community Intervention for k<210 and FICI for k>=210


Analysis for willingness to pay parameter k = 780

                       Expected utility
Community Intervention     288594252379
```

```
Facility Intervention      287221550988
FICI                       288916001484

                                                       EIB    CEAC     ICER
Community Intervention vs Facility Intervention 1372701391 0.99875  -2.9227
Community Intervention vs FICI                  -321749105 0.23855 205.0786

Optimal intervention (max expected utility) for k=780: FICI

EVPI 63426113
quartz
     2
[1] "time horizon in years"
[1] 10
[1] "cost discount rate"
[1] 0.03
[1] "effects discount rate"
[1] 0

Cost-effectiveness analysis summary

Reference intervention: Community Intervention
Comparator intervention(s): Facility Intervention
                          : FICI

Optimal decision: choose Community Intervention for k<210 and FICI for k>=210


Analysis for willingness to pay parameter k = 780

                       Expected utility
Community Intervention     630157093293
Facility Intervention      627159407405
FICI                       630857667962

                                                       EIB   CEAC     ICER
Community Intervention vs Facility Intervention 2997685888 0.9990  -3.0089
Community Intervention vs FICI                  -700574669 0.1505 206.6982

Optimal intervention (max expected utility) for k=780: FICI

EVPI 59381827
quartz
     2
[1] "time horizon in years"
[1] 10
[1] "cost discount rate"
[1] 0
[1] "effects discount rate"
[1] 0

Cost-effectiveness analysis summary

Reference intervention: Community Intervention
Comparator intervention(s): Facility Intervention
                          : FICI

Optimal decision: choose Community Intervention for k<245 and FICI for k>=245


Analysis for willingness to pay parameter k = 780

                       Expected utility
Community Intervention     630116336969
Facility Intervention      627116667949
FICI                       630774172181

                                                       EIB  CEAC     ICER
Community Intervention vs Facility Intervention 2999669020 0.999  -3.5269
Community Intervention vs FICI                  -657835212 0.166 241.6732

Optimal intervention (max expected utility) for k=780: FICI

EVPI 66144334
quartz
     2
[1] "time horizon in years"
[1] 10
[1] "cost discount rate"
[1] 0.1
[1] "effects discount rate"
[1] 0

Cost-effectiveness analysis summary
```

```
Reference intervention: Community Intervention
Comparator intervention(s): Facility Intervention
                          : FICI

Optimal decision: choose Community Intervention for k<150 and FICI for k>=150


Analysis for willingness to pay parameter k = 780

                       Expected utility
Community Intervention     630223245104
Facility Intervention      627228778048
FICI                       630993190415

                                                      EIB  CEAC     ICER
Community Intervention vs Facility Intervention 2994467055 0.999  -2.1681
Community Intervention vs FICI                  -769945312 0.129 149.9301

Optimal intervention (max expected utility) for k=780: FICI

EVPI 49814291
quartz
     2
[1] "time horizon in years"
[1] 10
[1] "cost discount rate"
[1] 0.03
[1] "effects discount rate"
[1] 0.02

Cost-effectiveness analysis summary

Reference intervention: Community Intervention
Comparator intervention(s): Facility Intervention
                          : FICI

Optimal decision: choose Community Intervention for k<235 and FICI for k>=235


Analysis for willingness to pay parameter k = 780

                       Expected utility
Community Intervention     566019439361
Facility Intervention      563325570555
FICI                       566623038119

                                                      EIB   CEAC     ICER
Community Intervention vs Facility Intervention 2693868806 0.9990  -3.3497
Community Intervention vs FICI                  -603598757 0.1613 230.1099

Optimal intervention (max expected utility) for k=780: FICI

EVPI 57375378
quartz
     2
[1] "time horizon in years"
[1] 10
[1] "cost discount rate"
[1] 0
[1] "effects discount rate"
[1] 0.02

Cost-effectiveness analysis summary

Reference intervention: Community Intervention
Comparator intervention(s): Facility Intervention
                          : FICI

Optimal decision: choose Community Intervention for k<270 and FICI for k>=270


Analysis for willingness to pay parameter k = 780

                       Expected utility
Community Intervention     565978683037
Facility Intervention      563282831099
FICI                       566539542338

                                                      EIB   CEAC     ICER
Community Intervention vs Facility Intervention 2695851938 0.9990  -3.9263
Community Intervention vs FICI                  -560859301 0.1781 269.0464

Optimal intervention (max expected utility) for k=780: FICI
```

```
EVPI 64640722
quartz
     2
[1] "time horizon in years"
[1] 10
[1] "cost discount rate"
[1] 0.1
[1] "effects discount rate"
[1] 0.02

Cost-effectiveness analysis summary

Reference intervention: Community Intervention
Comparator intervention(s): Facility Intervention
                          : FICI

Optimal decision: choose Community Intervention for k<170 and FICI for k>=170


Analysis for willingness to pay parameter k = 780

                       Expected utility
Community Intervention     566085591172
Facility Intervention      563394941198
FICI                       566758560572

                                                      EIB    CEAC     ICER
Community Intervention vs Facility Intervention 2690649974 0.99900  -2.4136
Community Intervention vs FICI                  -672969400 0.13335 166.9120

Optimal intervention (max expected utility) for k=780: FICI

EVPI 47200900
quartz
     2
[1] "time horizon in years"
[1] 10
[1] "cost discount rate"
[1] 0.03
[1] "effects discount rate"
[1] 0.03

Cost-effectiveness analysis summary

Reference intervention: Community Intervention
Comparator intervention(s): Facility Intervention
                          : FICI

Optimal decision: choose Community Intervention for k<245 and FICI for k>=245


Analysis for willingness to pay parameter k = 780

                       Expected utility
Community Intervention     537501350668
Facility Intervention      534942570721
FICI                       538061830174

                                                      EIB    CEAC     ICER
Community Intervention vs Facility Intervention 2558779947 0.99900  -3.5273
Community Intervention vs FICI                  -560479506 0.16695 242.3134

Optimal intervention (max expected utility) for k=780: FICI

EVPI 56627976
quartz
     2
[1] "time horizon in years"
[1] 10
[1] "cost discount rate"
[1] 0
[1] "effects discount rate"
[1] 0.03

Cost-effectiveness analysis summary

Reference intervention: Community Intervention
Comparator intervention(s): Facility Intervention
                          : FICI

Optimal decision: choose Community Intervention for k<285 and FICI for k>=285
```

```
Analysis for willingness to pay parameter k = 780

                       Expected utility
Community Intervention     537460594344
Facility Intervention      534899831264
FICI                       537978334393

                                                       EIB   CEAC     ICER
Community Intervention vs Facility Intervention 2560763079 0.9990  -4.1345
Community Intervention vs FICI                  -517740049 0.1855 283.3147

Optimal intervention (max expected utility) for k=780: FICI

EVPI 64158023
quartz
     2
[1] "time horizon in years"
[1] 10
[1] "cost discount rate"
[1] 0.1
[1] "effects discount rate"
[1] 0.03

Cost-effectiveness analysis summary

Reference intervention: Community Intervention
Comparator intervention(s): Facility Intervention
                          : FICI

Optimal decision: choose Community Intervention for k<180 and FICI for k>=180


Analysis for willingness to pay parameter k = 780

                       Expected utility
Community Intervention     537567502478
Facility Intervention      535011941364
FICI                       538197352627

                                                       EIB    CEAC     ICER
Community Intervention vs Facility Intervention 2555561115 0.99900  -2.5416
Community Intervention vs FICI                  -629850149 0.13725 175.7638

Optimal intervention (max expected utility) for k=780: FICI

EVPI 46091319
quartz
     2
[1] "time horizon in years"
[1] 20
[1] "cost discount rate"
[1] 0.03
[1] "effects discount rate"
[1] 0

Cost-effectiveness analysis summary

Reference intervention: Community Intervention
Comparator intervention(s): Facility Intervention
                          : FICI

Optimal decision: choose Community Intervention for k<180 and FICI for k>=180


Analysis for willingness to pay parameter k = 780

                       Expected utility
Community Intervention    1260379247717
Facility Intervention     1254386830995
FICI                      1261848413252

                                                        EIB CEAC     ICER
Community Intervention vs Facility Intervention  5992416723 1.00  -2.6229
Community Intervention vs FICI                  -1469165534 0.07 178.8683

Optimal intervention (max expected utility) for k=780: FICI

EVPI 39332414
quartz
     2
[1] "time horizon in years"
[1] 20
[1] "cost discount rate"
[1] 0
```

```
[1] "effects discount rate"
[1] 0

Cost−effectiveness analysis summary

Reference intervention: Community Intervention
Comparator intervention(s): Facility Intervention
                          : FICI

Optimal decision: choose Community Intervention for k<240 and FICI for k>=240


Analysis for willingness to pay parameter k = 780

                       Expected utility
Community Intervention    1260237204107
Facility Intervention     1254237875788
FICI                      1261557414435

                                                        EIB  CEAC     ICER
Community Intervention vs Facility Intervention  5999328319 1.000  −3.5256
Community Intervention vs FICI                  −1320210328 0.093 239.8157

Optimal intervention (max expected utility) for k=780: FICI

EVPI 51683524
quartz
     2
[1] "time horizon in years"
[1] 20
[1] "cost discount rate"
[1] 0.1
[1] "effects discount rate"
[1] 0

Cost−effectiveness analysis summary

Reference intervention: Community Intervention
Comparator intervention(s): Facility Intervention
                          : FICI

Optimal decision: choose Community Intervention for k<105 and FICI for k>=105


Analysis for willingness to pay parameter k = 780

                       Expected utility
Community Intervention    1260555713989
Facility Intervention     1254571883810
FICI                      1262209932339

                                                        EIB  CEAC     ICER
Community Intervention vs Facility Intervention  5983830180 1.000  −1.5015
Community Intervention vs FICI                  −1654218349 0.051 103.1511

Optimal intervention (max expected utility) for k=780: FICI

EVPI 28034329
quartz
     2
[1] "time horizon in years"
[1] 20
[1] "cost discount rate"
[1] 0.03
[1] "effects discount rate"
[1] 0.02

Cost−effectiveness analysis summary

Reference intervention: Community Intervention
Comparator intervention(s): Facility Intervention
                          : FICI

Optimal decision: choose Community Intervention for k<220 and FICI for k>=220


Analysis for willingness to pay parameter k = 780

                       Expected utility
Community Intervention    1030374277350
Facility Intervention     1025471383468
FICI                      1031495676163

                                                        EIB    CEAC     ICER
```

```
Community Intervention vs Facility Intervention  4902893883 1.00000  -3.2082
Community Intervention vs FICI                  -1121398813 0.08695 218.7800

Optimal intervention (max expected utility) for k=780: FICI

EVPI 38677596
quartz
     2
[1] "time horizon in years"
[1] 20
[1] "cost discount rate"
[1] 0
[1] "effects discount rate"
[1] 0.02

Cost-effectiveness analysis summary

Reference intervention: Community Intervention
Comparator intervention(s): Facility Intervention
                          : FICI

Optimal decision: choose Community Intervention for k<295 and FICI for k>=295


Analysis for willingness to pay parameter k = 780

                       Expected utility
Community Intervention    1030232233740
Facility Intervention     1025322428261
FICI                      1031204677347

                                                       EIB  CEAC     ICER
Community Intervention vs Facility Intervention 4909805479 1.000  -4.3123
Community Intervention vs FICI                  -972443607 0.124 293.3268

Optimal intervention (max expected utility) for k=780: FICI

EVPI 54215061
quartz
     2
[1] "time horizon in years"
[1] 20
[1] "cost discount rate"
[1] 0.1
[1] "effects discount rate"
[1] 0.02

Cost-effectiveness analysis summary

Reference intervention: Community Intervention
Comparator intervention(s): Facility Intervention
                          : FICI

Optimal decision: choose Community Intervention for k<130 and FICI for k>=130


Analysis for willingness to pay parameter k = 780

                       Expected utility
Community Intervention    1030550743622
Facility Intervention     1025656436283
FICI                      1031857195250

                                                        EIB    CEAC     ICER
Community Intervention vs Facility Intervention  4894307339 1.00000  -1.8365
Community Intervention vs FICI                  -1306451628 0.05745 126.1677

Optimal intervention (max expected utility) for k=780: FICI

EVPI 25450120
quartz
     2
[1] "time horizon in years"
[1] 20
[1] "cost discount rate"
[1] 0.03
[1] "effects discount rate"
[1] 0.03

Cost-effectiveness analysis summary

Reference intervention: Community Intervention
Comparator intervention(s): Facility Intervention
                          : FICI
```

```
Optimal decision: choose Community Intervention for k<245 and FICI for k>=245


Analysis for willingness to pay parameter k = 780

                       Expected utility
Community Intervention     937456205820
Facility Intervention      932993460518
FICI                       938437112830

                                                  EIB    CEAC    ICER
Community Intervention vs Facility Intervention 4462745302 1.00000  -3.526
Community Intervention vs FICI                  -980907010 0.09985 240.455

Optimal intervention (max expected utility) for k=780: FICI

EVPI 39170730
quartz
     2
[1] "time horizon in years"
[1] 20
[1] "cost discount rate"
[1] 0
[1] "effects discount rate"
[1] 0.03

Cost-effectiveness analysis summary

Reference intervention: Community Intervention
Comparator intervention(s): Facility Intervention
                          : FICI

Optimal decision: choose Community Intervention for k<325 and FICI for k>=325


Analysis for willingness to pay parameter k = 780

                       Expected utility
Community Intervention     937314162210
Facility Intervention      932844505312
FICI                       938146114013

                                                  EIB    CEAC     ICER
Community Intervention vs Facility Intervention 4469656898 1.00000  -4.7395
Community Intervention vs FICI                  -831951803 0.14235 322.3876

Optimal intervention (max expected utility) for k=780: FICI

EVPI 57149945
quartz
     2
[1] "time horizon in years"
[1] 20
[1] "cost discount rate"
[1] 0.1
[1] "effects discount rate"
[1] 0.03

Cost-effectiveness analysis summary

Reference intervention: Community Intervention
Comparator intervention(s): Facility Intervention
                          : FICI

Optimal decision: choose Community Intervention for k<140 and FICI for k>=140


Analysis for willingness to pay parameter k = 780

                       Expected utility
Community Intervention     937632672092
Facility Intervention      933178513333
FICI                       938798631917

                                                   EIB   CEAC     ICER
Community Intervention vs Facility Intervention  4454158759 1.0000  -2.0185
Community Intervention vs FICI                  -1165959825 0.0615 138.6675

Optimal intervention (max expected utility) for k=780: FICI

EVPI 24582113
```
