# Supplementary material for: Cost-effectiveness and affordability of community mobilisation through women’s groups and quality improvement in health facilities (MaiKhanda trial) in Malawi
Source: Cost Eff Resour Alloc. 2015 Jan 10;13:1. doi: 10.1186/s12962-014-0028-2 (PMC4299571; doi:10.1186/s12962-014-0028-2)
Supplement: Additional file 2: — Calculation of MNH DALYs averted in Malawi in 2010 with ‘current practice’ interventions, cost-effectiveness of ‘current practice’ interventions, and proportion of total remaining DALYs the MaiKhanda interventions could avert. [1,2,14,23,24,38,53-56]. [file 12962_2014_28_MOESM2_ESM.pdf]

Additional file 2: Calculation of MNH DALYs averted in Malawi in 2010 with 'current practice' interventions, cost-effectiveness of 'current practice' interventions, and proportion of total remaining DALYs the MaiKhanda interventions could avert.

	Parameter	Estimate	Source[a]
A	Population (mothers or babies)	600,548[b]	[14] [2]
B	Natural MMR with no interventions	0.02000[c]	[1] [53]
C	MMR in 2010	0.00484	[1]
D	MMR averted by 2010-current interventions	0.01516	*B - C*
E	Maternal deaths averted by 2010-current interventions	9,104	*AD*
F	DALYs per maternal death	53.27[d]	[2] [24]
G	Maternal DALYs averted by 2010-current interventions	484,986	*EF*
H	Natural NMR with no interventions	0.100[e]	[54]
I	NMR in 2010	0.029	[55]
J	NMR averted by 2010-current interventions	0.071	*H - I*
K	Neonatal deaths averted by 2010-current interventions	42,639	*AJ*
L	DALYs per neonatal death	86.0[f]	[23]
M	Neonatal DALYs averted by 2010-current interventions	3,666,945	*KL*
N	Natural SBR with no interventions	0.100[g]	[56]
O	SBR in 2010	0.024[h]	[56]
P	SBR averted by 2010-current interventions	0.076	*N - O*
Q	Stillbirths averted by 2010-current interventions	45,642	*AP*
R	DALYs per stillbirth	86.0[f]	[23]
S	Stillbirth DALYs averted by 2010-current interventions	3,925,180	*QR*
T	Total stillbirth, neonatal and maternal DALYs averted by 2010-current interventions	8,077,111	*G + M + S*
U	Total MNH expenditure ($)	410,354,347[i]	[14] [38]
V	ICER of 'current practice' ($ per DALY averted)	50.80	*U / T*
W	Maternal DALYs remaining	154,837	*ACF*
X	Neonatal DALYs remaining	1,497,766	*AIL*
Y	Stillbirth DALYs remaining	1,239,531	*AOR*
Z	Total MNH DALYs remaining	2,892,134	*W + X + Y*
Aa	DALYs averted by CI if in whole of Malawi in 2010	374,569[j]	This paper
Ab	DALYs averted by FI if in whole of Malawi in 2010	110,662[k]	This paper
Ac	DALYs averted by FICI if in whole of Malawi in 2010	418,048[l]	This paper
Ad	% of total remaining MNH DALYs averted CI	13.0%	*Aa / Z*
Ae	% of total remaining MNH DALYs averted FI	3.8%	*Ab / Z*
Af	% of total remaining MNH DALYs averted FICI	14.5%	*Ac / Z*

MNH = Maternal and Neonatal Health; DALY = Disability Adjusted Life Year; MMR = Maternal Mortality Rate – deaths of mothers as a proportion of total population of women giving birth (note this is a rough approximation of the maternal mortality ratio, for the purposes of these calculations); NMR = Neonatal Mortality Rate – deaths of babies within 28 days of birth as a proportion of all births (note this is a rough approximation of the neonatal mortality rate, for the purposes of these calculations); SBR = Stillbirth Rate – stillborn babies as a proportion of all babies (note this is also a rough approximation for the purposes of these calculations); $ constant 2013 international dollars; ICER = Incremental Cost-Effectiveness Ratio; CI = Community Intervention; FI = Facility Intervention; FICI = Facility and Community Interventions combined

[a] Reference list in main paper (note references 53-56 are only found in Additional file 2). See footnotes for further details. Calculations with italic capital letters refer to named rows of this table.

[b] This is the estimate of the total population in Malawi in 2010: 15,013,694 [14] multiplied by an estimated Crude Birth Rate (CBR) of 0.04 per person per year [2]. As we assume one baby per mother per year on average, this is an estimate of the total mothers delivering, and also of the total babies delivered in Malawi in 2010.

[c] Inflated from the estimate of 1500 per 100,000 livebirths in [53], bearing in mind MMR was estimated to be as high as 970 maternal deaths per 100,000 livebirths in 2000 even with some medical intervention available [1].

[d] The number of maternal deaths was multiplied by 53.27, the remaining standard life expectancy of females aged 30 [24], the median age of maternal death in Malawi in 2010 [2][Table 16.3, page 222] DALYS were calculated with no age weights and no discounting, consistent with the Global Burden of Disease 2010 study [23].

[e] We could not find an estimate of the NMR without interventions so have extrapolated upwards from 78 per 1000, the upper 95%CI of the estimated NMR in Afghanistan in 1990, the highest estimate of NMR provided by Oestergaard *et al* [54][Web Appendix] on the assumption that there was still some medical care available in Afghanistan in 1990 even despite this high NMR.

[f] The number of stillbirths and neonatal deaths averted was multiplied by 86.0, the standard life expectancy at birth used in the Global Burden of Disease 2010 study [23].

[g] We could not find an estimate of the SBR without interventions so have extrapolated upwards from 78 per 1000, the upper 95%CI of the estimated SBR in Pakistan in 2009 and the highest estimate of SBR provided by Cousens *et al* [56][Web Appendix] on the assumption that there was still some medical care available in Pakistan in 2009 even despite this high SBR.

[h] Estimate for Malawi in 2009 [56].

[i] World Bank (WB) data suggests the per capital expenditure on health in Malawi in 2010 was $72.7 per year in constant 2005 international dollars, which using WB PPP conversion factors of local currency units per international dollar for 2005 and 2013 translates to $228.7 constant 2013 international dollars and the total population of Malawi in 2010 was 15,013,694 [14]. The proportion of total health expenditure spent on MNH in 2010 was estimated to be 0.1195 [38]. Multiplying these figures yields an estimate of $410,354,347 constant 2013 international dollars for the total annual expenditure on MNH in Malawi in 2010.

[j] DALYs averted by the trial in one year (estimate of 67,361 for 27-month trial period (see Table 1 of main paper) divided by 2.25) multiplied by 12.51, which is the ratio of the trial population to the total population (see Table 2 of main paper).

[k] DALYs averted by the trial in one year (estimate of 19,901 for 27-month trial period (see Table 1 of main paper) divided by 2.25) multiplied by 12.51, which is the ratio of the trial population to the total population (see Table 2 of main paper).

[l] DALYs averted by the trial in one year (estimate of 37,590 for 27-month trial period (see Table 1 of main paper) divided by 2.25) multiplied by 12.51, which is the ratio of the trial population to the total population (see Table 2 of main paper), multiplied by two to reflect what the effects of FICI would have been if in the same area (two arms) of the trial as CI or FI (so that the trial to scale-up population ratio of 12.51 is still valid).
