# Supplementary material for: Cost-effectiveness and affordability of community mobilisation through women’s groups and quality improvement in health facilities (MaiKhanda trial) in Malawi
Source: Cost Eff Resour Alloc. 2015 Jan 10;13:1. doi: 10.1186/s12962-014-0028-2 (PMC4299571; doi:10.1186/s12962-014-0028-2)
Supplement: Additional file 3: — Comparison of results with standard and local life expectancies. The number of deaths averted (the effects of the interventions) were converted to Disability Adjusted Life Years (DALYs) averted. As the base-case, ‘standard’ life expectancy was used to calculate DALYs. The number of stillbirths and neonatal deaths averted was multiplied by 86.0, the standard life expectancy at birth used in the Global Burden of Disease 2010 study [23]. The number of maternal deaths was multiplied by 53.27, the remaining standard life expectancy of females aged 30 [24], the median age of maternal death in Malawi in 2010 [2]Table 16.3, page 222. As a sensitivity analysis, and as recommended by Polinder et al [25], we also calculated ‘DALYs’ using ‘local’ life expectancy: remaining healthy life expectancies specific to Malawi: 45.0 years, the healthy life expectancy at birth in Malawi in 2010 [26] for stillbirths and neonatal deaths averted, and 28.1 years, the healthy life expectancy at the median age of maternal death (30 years) in Malawi in 2010 [2]Table 16.3, page 222 [26,27] for maternal deaths averted. The results of both analyses are shown side-by-side in the table to aid comparison. [file 12962_2014_28_MOESM3_ESM.pdf]

Additional file 3: Comparison of results with standard and local life expectancies

The number of deaths averted (the effects of the interventions) were converted to Disability Adjusted Life Years (DALYs) averted.

As the base-case, 'standard' life expectancy was used to calculate DALYs. The number of stillbirths and neonatal deaths averted was multiplied by 86.0, the standard life expectancy at birth used in the Global Burden of Disease 2010 study [23]. The number of maternal deaths was multiplied by 53.27, the remaining standard life expectancy of females aged 30 [24], the median age of maternal death in Malawi in 2010 [2][Table 16.3, page 222].

As a sensitivity analysis, and as recommended by Polinder *et al* [25], we also calculated 'DALYs' using 'local' life expectancy: remaining healthy life expectancies specific to Malawi: 45.0 years, the healthy life expectancy at birth in Malawi in 2010 [26] for stillbirths and neonatal deaths averted, and 28.1 years, the healthy life expectancy at the median age of maternal death (30 years) in Malawi in 2010 [2][Table 16.3, page 222] [26, 27] for maternal deaths averted.

The results of both analyses are shown side-by-side in the table below to aid comparison.

Comparison	Life Expectancy	ICER	Probability cost-effective	EIB [a] ($) at λ	EVPI [b] at λ
CI vs. 0	Standard	$79	98%	47,192,509	210,423
	Local	$152	97%	22,174,019	170,677
FI vs. 0	Standard	$281	66%	9,930,509	5,851,010
	Local	$548	58%	2,373,589	4,144,723
FICI vs. 0	Standard	$146	93%	23,849,857	598,177
	Local	$277	88%	9,925,614	534,291
FICI vs. CI	Standard	$292	60%	9,334,580	11,708,726
	Local	$555	55%	2,269,803	7,212,170

$ = constant 2013 international dollars; CI = MaiKhanda Community Intervention; 0 = current practice (do nothing); FI = MaiKhanda Facility Intervention; FICI = MaiKhanda combined Facility and Community Intervention; ICER = Incremental Cost-Effectiveness Ratio; λ = $780 per DALY averted, the Malawian 2013 per capita GDP threshold of 'highly cost-effective' interventions

[a] Expected Incremental Benefit – the $ value of the additional DALYs averted.

[b] Expected Value of Information- the monetary ($) value of reducing uncertainty in the model parameters through additional research. It is calculated by comparing the EIB of the current decision with the probable EIB given additional information on the model parameters. EVI can be compared with the EIB (both at specified values of λ) to determine if spending additional money on research to reduce parameter uncertainty might be worthwhile.
