# Supplementary material for: Cost-effectiveness and affordability of community mobilisation through women’s groups and quality improvement in health facilities (MaiKhanda trial) in Malawi
Source: Cost Eff Resour Alloc. 2015 Jan 10;13:1. doi: 10.1186/s12962-014-0028-2 (PMC4299571; doi:10.1186/s12962-014-0028-2)
Supplement: Additional file 4: — Future Scale-up scenarios. Scale-up to the whole population of Malawi, using the trial population to total population ratio explained in Table 2 of the paper. Annual maintenance, implementation and external costs were used from Table 1 of the paper, and were discounted using the costs discount rate for the scenario and added to the scaled-up beginning start-up costs. Effects were annualised by dividing the estimates from the 27-month trial by 2.25, and discounted according to the effects discount rate of the scenario. Note that all models were run with only 20000 saved Monte Carlo simulations from 2 chains of 21000 simulations with a burn-in of 1000 and thinning of 2 (as compared to the 100,000 saved simulations from the main analysis), but adequate convergence of these models was achieved (Additional file 1). [file 12962_2014_28_MOESM4_ESM.pdf]

Additional file 4: Future Scale-up scenarios

Scale-up to the whole population of Malawi, using the trial population to total population ratio explained in Table 2 of the paper. Annual maintenance, implementation and external costs were used from Table 1 of the paper, and were discounted using the costs discount rate for the scenario and added to the scaled-up beginning start-up costs. Effects were annualised by dividing the estimates from the 27-month trial by 2.25, and discounted according to the effects discount rate of the scenario. Note that all models were run with only 20000 saved Monte Carlo simulations from 2 chains of 21000 simulations with a burn-in of 1000 and thinning of 2 (as compared to the 100,000 saved simulations from the main analysis), but adequate convergence of these models was achieved (Additional file 1).

Scenario	Time Horizon	Cost discount rate (per year)	Effects discount rate (per year)	EIB[a]($) for CI vs FI at λ	Probability CI cost-effective compared to FI at λ	$ per DALY threshold at which FICI becomes more cost-effective than CI[b]	EIB [a]($) for FICI vs CI at λ	Probability FICI cost-effective compared to CI at λ
1.	5 years	0%	0%	1,499,839,371	99.9%	$245	326,647,654	74.6%
2.	5 years	3%	0%	1,499,272,288	99.9%	$225	338,869,127	75.4%
3.	5 years	10%	0%	1,498,207,833	99.9%	$188	361,809,728	76.8%
4.	5 years	0%	2%	1,414,273,602	99.9%	$260	299,335,766	74.1%
5.	5 years	3%	2%	1,413,706,520	99.9%	$239	311,557,239	74.8%
6.	5 years	10%	2%	1,412,642,064	99.9%	$199	334,497,839	76.4%
7.	5 years	0%	3%	1,374,332,929	99.9%	$268	286,587,031	73.8%
8.	5 years	3%	3%	1,373,765,846	99.9%	$246	298,808,504	74.6%
9.	5 years	10%	3%	1,372,701,391	99.9%	$205	321,749,105	76.1%
10.	10 years	0%	0%	2,999,669,020	99.9%	$242	657,835,212	83.4%
11.	10 years	3%	0%	2,997,685,888	99.9%	$207	700,574,669	85.0%
12.	10 years	10%	0%	2,994,467,055	99.9%	$150	769,945,312	87.1%
13.	10 years	0%	2%	2,695,851,938	99.9%	$269	560,859,301	82.2%
14.	10 years	3%	2%	2,693,868,806	99.9%	$230	603,598,757	83.9%
15.	10 years	10%	2%	2,690,649,974	99.9%	$167	672,969,400	86.7%
16.	10 years	0%	3%	2,560,763,079	99.9%	$283	517,740,049	81.5%
17.	10 years	3%	3%	2,558,779,947	99.9%	$242	560,479,506	83.3%
18.	10 years	10%	3%	2,555,561,115	99.9%	$176	629,850,149	86.3%
19.	20 years	0%	0%	5,999,328,319	100.0%	$240	1,320,210,328	90.7%
20.	20 years	3%	0%	5,992,416,723	100.0%	$179	1,469,165,534	93.0%
21.	20 years	10%	0%	5,983,830,180	100.0%	$103	1,654,218,349	94.9%
22.	20 years	0%	2%	4,909,805,479	100.0%	$293	972,443,607	87.6%
23.	20 years	3%	2%	4,902,893,883	100.0%	$219	1,121,398,813	91.3%
24.	20 years	10%	2%	4,894,307,339	100.0%	$126	1,306,451,628	94.3%
25.	20 years	0%	3%	4,469,656,898	100.0%	$322	831,951,803	85.8%
26.	20 years	3%	3%	4,462,745,302	100.0%	$240	980,907,010	90.0%
27.	20 years	10%	3%	4,454,158,759	100.0%	$139	1,165,959,825	93.9%

$ = constant 2013 international dollars; CI = MaiKhanda Community Intervention; FI = MaiKhanda Facility Intervention; FICI = MaiKhanda combined Facility and Community Intervention; λ = $780 per DALY averted, the Malawian 2013 per capita GDP threshold of 'highly cost-effective' interventions

[a] Expected Incremental Benefit – the $ value of the additional DALYs averted. Note that more DALYs are averted the longer the time horizon is.

[b] FI was always dominated by CI (and FICI) in all scenarios and at all thresholds. As the time horizon and cost discount rate increases, and the effects discount rate decreases, the $ per DALY threshold at which FICI becomes the optimal decision (the ICER of CI vs FICI) decreases. $ per DALY threshold rounded to the nearest dollar.
