# Supplementary material for: Cost-effectiveness and affordability of community mobilisation through women’s groups and quality improvement in health facilities (MaiKhanda trial) in Malawi
Source: Cost Eff Resour Alloc. 2015 Jan 10;13:1. doi: 10.1186/s12962-014-0028-2 (PMC4299571; doi:10.1186/s12962-014-0028-2)
Supplement: Additional file 5: — How the cost Effectiveness of the MaiKhanda interventions compares to the Essential Health Package currently used in Malawi. [file 12962_2014_28_MOESM5_ESM.pdf]

Additional file 5: How the cost-effectiveness of the MaiKhanda interventions compares to the Essential Health Package currently used in Malawi

This is Figure 14 of the Health Sector Strategic Plan of the Government of Malawi Ministry of Health[1] reproduced in full with the MaiKhanda interventions added as coloured stars.

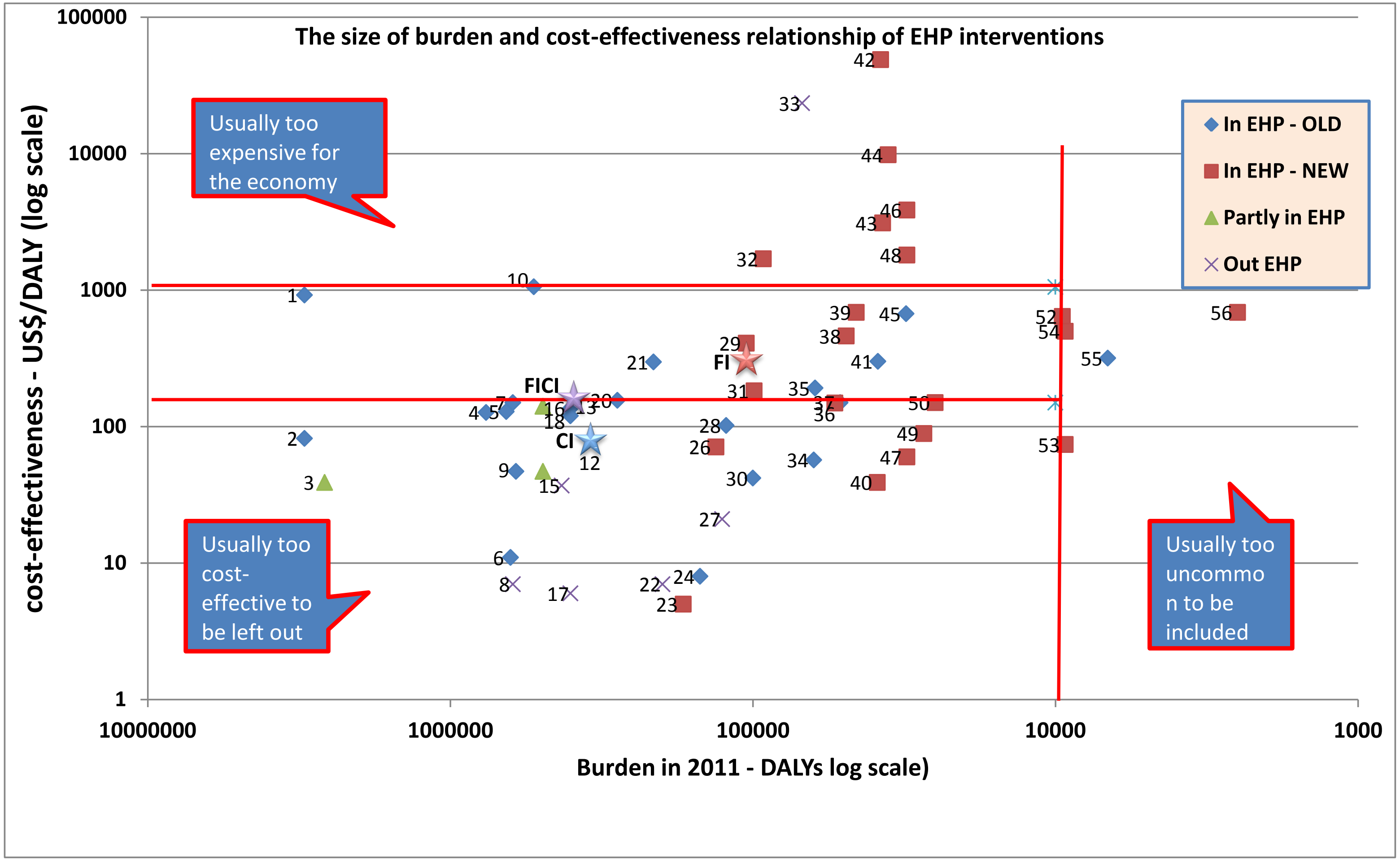

1-ARV; 2-HIV prevention; 3-IMCI; 4-Maternal care; 5-ARI in under-5s; 6-Malaria – bednets; 7-Malaria in under 5s using ACT; 8-IPT child; 9-HCT; 10-Dehydration Thanzi; 11-Home made ORS; 12-Water supply; 13-Improved sanitation; 14-Family planning; 15-School health; 16-Wounds, fractures; 17-First aid training of volunteers; 18-Emergency ambulance service; 19-IRS; 20-Management of OIs; 21- Penta vaccine; 22-DPT Polio; 23-NTD mass treatment; 24-Measles vaccine; 25-Supplementary Feeding; 26-Rotavirus vaccine; 27-Prevention of RTAs; 28-Treatment - smear pos. TB; 29-BP - polypill; 30-Growth monitoring; 31-Cataract extraction; 32-Depression; 33-Cancer; 34-STIs; 35-PMTCT; 36-Aspirin for stroke; 37-Malaria in 4-year-olds + ACT; 38-Anxiety disorders; 39-IHD - drugs; 40- Trachoma surgery; 41-Treatment - smear neg. TB; 42-Drug misuse; 43-Bipolar disorders; 44-Schizophrenia; 45-CBHBC; 46-Diabetes - screening; 47-Diabetes - lifestyle change; 48-Diabetes - drugs; 49-Epilepsy; 50-CCF drugs; 51-IPT Preg; 52-Alcohol misuse - PC advice; 53-Cervical screening - cryotherapy; 54-HPV; 55-TB relapsed; 56-Rheumatic h d[2].

CI = MaiKhanda Community Intervention; **FI** = MaiKhanda Facility Intervention; **FICI** = MaiKhanda Facility and Community Interventions combined

[1]Government of Malawi Ministry of Health: Health Sector Strategic Plan 2011-2016. Moving towards equity and quality Lilongwe: Ministry of Health; 2011.

[2]BoD 2011 College of Medicine Cost-effectiveness ratios from BDDC 2nd Edition 2006.
